# Evidence of health inequities across the rare disease patient care pathway: development of a toolkit using a conceptual framework

**DOI:** 10.1186/s13023-026-04389-0

**Published:** 2026-06-02

**Authors:** Simon Briscoe, Clara Martin Pintado, Ruth Garside, Jo Thompson Coon, Hassanat M. Lawal, Noreen Orr, Liz Shaw, G. J. Melendez-Torres

**Affiliations:** Exeter PRP Evidence Review Facility, University of Exeter Medical School, St Luke’s Campus, University of Exeter, Exeter, Devon, EX1 2LU UK

## Abstract

People with a rare disease (PwRD) often face more challenges than the wider population in obtaining a diagnosis and accessing services. These challenges can be framed as health inequities, i.e. differences in health opportunities and outcomes which are avoidable, systemic and unfair. We previously undertook a scoping review on the extent and nature of evidence on health inequities experienced by PwRD in relation to diagnosis and service access. In this follow up paper, we describe how we used a conceptual framework to develop a toolkit which maps the identified health inequities across the patient care pathway. The toolkit aims to show how health inequities are structurally interrelated, and where targeted interventions are needed to address inequity. Using Diderichsen and Hallqvist’s framework for describing pathways from social contexts to health outcomes, we categorised the identified health inequities as mechanisms and outcomes which were mapped onto contexts in the patient care pathway. The toolkit is presented as an interactive slide deck which facilitates the exploration of different types of inequity across the patient care pathway, using hyperlinks which take the user to more information on each of the inequities. This is structured in two main sections: (1) receipt of a diagnosis and (2) access to services after diagnosis. The focus is on UK settings. We anticipate that the toolkit can be used to raise awareness of health inequities for PwRD, and to inform the development of interventions for addressing health inequities. The toolkit is accessible in an accompanying file to this paper.

**Supplementary Information:**

The online version contains supplementary material available at 10.1186/s13023-026-04389-0.

## Background

Health inequities are differences in health opportunities and outcomes which are avoidable, systemic and unfair [1]. We were commissioned by the United Kingdom (UK) National Institute for Health and Care Research (NIHR) Policy Research Programme to undertake a scoping review of health inequities experienced by the rare disease community in the UK with respect to receipt of a diagnosis and access to health and social care services. This followed the commitment of the England Rare Disease Action Plan 2023 to work towards addressing inequities experienced by the rare disease community in these areas [2]. The resulting scoping review was conducted in adherence to established methodological guidance, including with patient and public involvement (PPI), [3] and published in this journal [4]. The review identified 136 relevant studies, including 96 UK primary studies and 40 international systematic reviews, within which seventeen types of health inequity experienced by the rare disease community were identified. [4] Eleven of the identified inequities related to disparities between the rare disease community and the general population (see Table 1). The remaining six related to sub-groups within the rare disease community, which served to exacerbate the eleven inequities experienced across the community as a whole. These sub-group characteristics were identified with reference to the PROGRESS+ health inequities framework and comprised place of residence, race/ethnicity, gender/sex, socioeconomic status, age and disability [5].

**Table 1:** Types of inequity experienced between the rare disease community and general population

Type of inequity	Description
Delayed diagnosis	Delays to receiving a diagnosis were experienced by PwRD.
Lack of knowledge	A lack of knowledge amongst clinicians could sometimes impact on receipt of a diagnosis of a rare disease and ability of PwRD to access appropriate services.
Lack of information	Clinicians would sometimes not provide or signpost to PwRD to sufficient information about a rare disease.
Limited services provision (comprising six types of inequity)	Challenges with accessing appropriate services, often related to limited services provision but also more specific challenges within different types of services. These included mental health services, emergency services, dentistry services, specialist services, social care services and services in general where these were not specified.
Limited services for undiagnosed conditions	Specific challenges amongst those with symptoms of a rare disease around accessing services when they did not have a diagnosis of a rare condition.
Lack of care co-ordination	Lack of care co-ordination between different health and social care services could adversely affect the receipt of a diagnosis and the ability to access accessing services after a diagnosis was received.

Abbreviations: PwRD=people with a rare disease

The scoping review narratively summarised the identified health inequities as they related to receipt of a diagnosis and access to health and social care services, drawing out where these data were derived from UK-based studies [4]. This established an understanding of the available evidence on the extent and nature of health inequities experienced by people with a rare disease (PwRD) in the UK. However, it did not show how the identified health inequities were structurally interrelated across the patient care pathway – for example, how inequities such as lack of knowledge or lack of information were experienced across primary, secondary and specialist services when seeking a diagnosis or accessing services, and how this affected outcomes such as delayed diagnosis. This relatively narrow focus reflects how scoping reviews aim to map the extent and nature of available evidence rather than interpret it in depth [3].

An understanding of the structural interrelation of inequities experienced by the rare disease community—showing where and how PwRD experience health inequities across the patient care pathway, and the implications for diagnosis and service access—could be used to inform the development of targeted interventions aimed at addressing these inequities, thus further supporting the commitment of the England Rare Diseases Action Plan 2023 [2]. To bridge the gap between the scoping review and the understanding required to inform practical action, we undertook further work to develop a toolkit which allows users to visualise and explore the structural interrelation of inequities experienced by PwRD across the patient care pathway. The toolkit was developed through further analysis of the data from the scoping review, [4] with reference to Diderichsen and Hallqvist’s framework for describing pathways from social contexts to health outcomes [6]. Specifically, the toolkit aims to support the identification of *contexts* along the patient care pathway that are associated with *mechanisms* leading to differential *outcomes*. In this respect, the toolkit also makes use of Pawson’s realist Context-Mechanism-Outcome heuristic as an interpretive lens, but does not comprise a full realist evaluation [7]. Additionally, the toolkit highlights where evidence indicates that the identified mechanisms and outcomes are further exacerbated for sub-groups within the rare disease community.

In this paper, we describe how the toolkit was developed and how to access and use it.

## Development of the toolkit

Diderichsen and Hallqvist’s framework aims to show how societies generate and maintain health inequities [6]. Specifically, Diderichsen and Hallqvist’s framework sets out an overarching context (society), within which there are mechanisms (causal explanations) which lead to differences in health outcomes for individuals and groups (health inequities) [6]. Furthermore, they intend that their framework is used to identify policy entry points for targeted interventions to address health inequities, by drawing attention to which mechanisms are producing which outcomes [6].

**Fig. 1:**
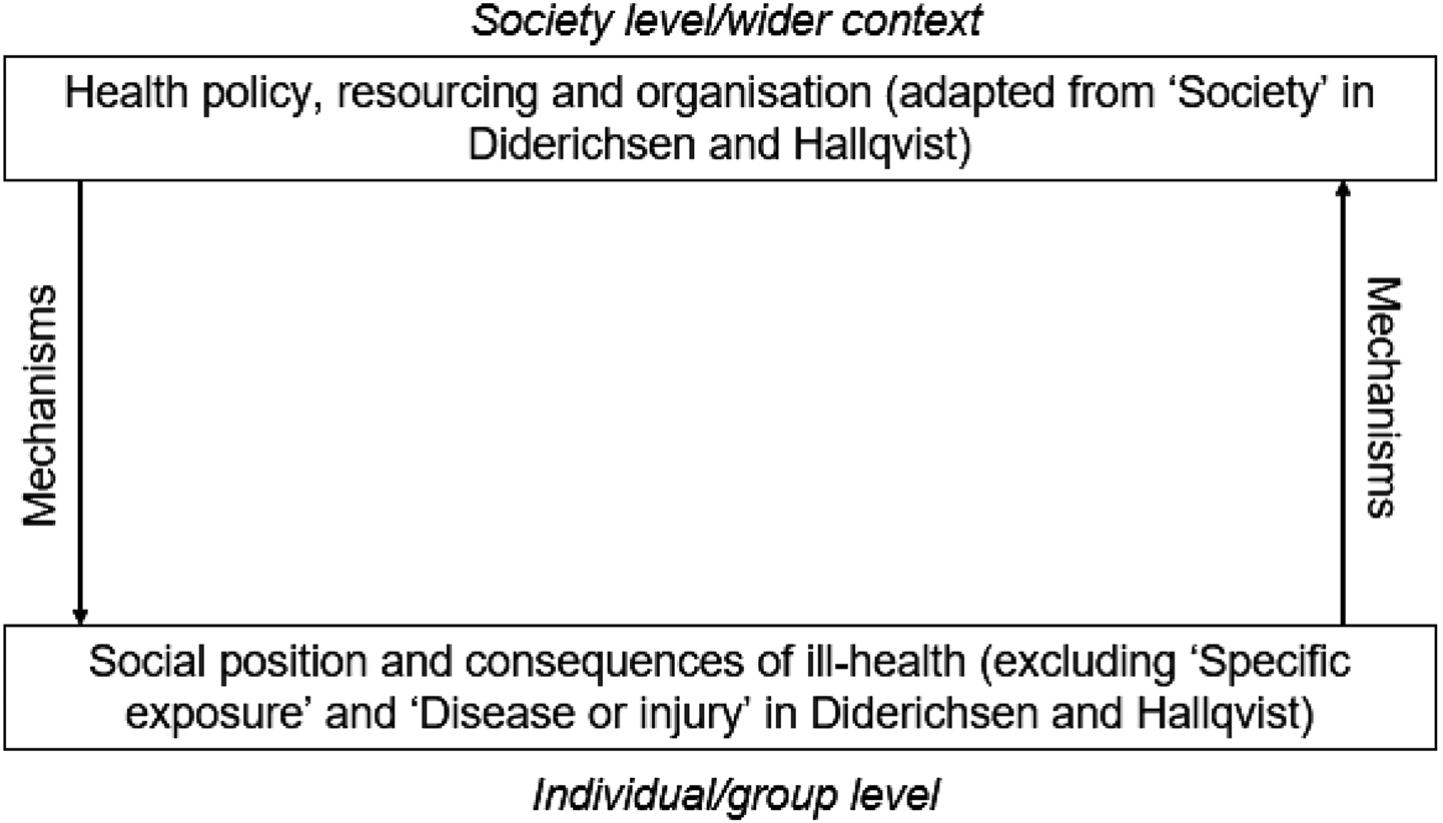
Adaptations to Diderichsen and Hallqvist’s framework

For the purpose of our toolkit, we adapted the overarching societal context in Diderichsen and Hallqvist’s framework to focus on *health policy*,* resourcing and organisation*, which reflects our aim to facilitate understanding of health inequities in the relatively narrow context of health and social care services delivery (see Fig. 1) [6]. At the individual/group level (i.e. the overall impact that the mechanisms within the framework have on individuals and groups within society) we focused our toolkit on *social position and consequences of ill-health*; thus, we removed Diderichsen and Hallqvist’s references to *specific exposure* or *disease/injury* at the individual/group level. We removed specific exposure because the majority of rare diseases are genetic, thus exposure is less relevant from the point of view of health inequity; and we removed disease or injury because, relatedly, we are solely interested in people who are already presenting with symptoms of disease, rather than where disease is a possible outcome relating to health inequity. Within this there are mechanisms which lead to disparities at the individual/group level.

Within this revised version of the framework, we structured the health and social care settings which the data for our scoping review showed that PwRD engage with into a patient care pathway, differentiating between receipt of a diagnosis and subsequent access to services [4]. Some of these contexts were explicitly reported in the scoping review (see services listed under *Limited services provision* in Table 1) [4]. However, the process of identifying relevant contexts also involved re-visiting the raw data in the data-extraction forms for data relating to the setting of health inequities which were not always reported in Briscoe et al., particularly where inequities which spanned several different contexts were not differentiated – for example, contexts relating to lack of knowledge and lack of information, which spanned primary, secondary and specialist services as well as social care [4]. In some studies the specific contexts were not clear, thus we were not able to use all 96 UK primary studies included in the scoping review [4]. We focused on data derived from UK-based studies, but also included international evidence from systematic reviews relating to one context where there was no UK evidence (maternity services).

Once we had mapped out the relevant health and social care contexts into a pathway, we linked each context to the various types of inequity identified in Briscoe et al. that PwRD experience within them [4]. For the purpose of the toolkit, these were described in terms of mechanisms (e.g. lack of knowledge amongst clinicians, lack of information provision, or lack of care co-ordination) which lead to differences in outcomes (e.g. delayed diagnosis). We also sought to identify within the data where these mechanisms and outcomes are experienced differently for specific groups within the rare disease community, with reference to the PROGRESS+ framework [5]. As with the identification of contexts, this process involved re-visiting the raw data in the data extraction forms. This sometimes involved additional interpretation of data which led to identifying new mechanisms and outcomes which were not described, or given distinct labels, in the scoping review. The full set of mechanisms and outcomes described in the toolkit are presented in Table 2, including those initially identified from Table 1.

**Table 2:** Revised set of types of inequity experienced by people with a rare disease, re-framed as mechanisms and outcomes

Type of inequity	Description
*Mechanisms*	
Accessibility	Accessibility issues included long wait times to access health and social care services and lack of availability of services.
Dismissive attitudes	Clinicians were sometimes dismissive of the symptoms of a rare disease, including with respect to the possibility of securing a diagnosis, and with respect to the impact of symptoms on daily living.
Lack of care co-ordination	Lack of care co-ordination between different health and social care services could adversely affect the receipt of a diagnosis and the ability to access services after a diagnosis was received.
Lack of information	Clinicians would sometimes not provide or signpost PwRD to sufficient information about a rare disease.
Lack of knowledge	A lack of knowledge amongst clinicians and social care staff could sometimes impact on receipt of a diagnosis (e.g. timeliness) and the ability for PwRD to access appropriate services.
Limited data sets	Limited data sets were identified as a limitation for people seeking a diagnosis of a genetic rare disease, e.g. limited representation of ethnic minorities in data sets.
Wrong specialist	PwRD were sometimes referred to the wrong specialist for further tests, which impacted on the timeliness of diagnosis and the ability to access appropriate services.
*Outcomes*	
Delayed diagnosis	Delayed diagnosis was a commonly reported outcome of the inequities experienced when seeking a diagnosis and accessing services.
No- or mis-diagnosis	PwRD were sometimes given the wrong diagnosis or had their symptoms dismissed.
Not listened to	Not feeling listened to was reported by PwRD as a consequence of having their symptoms dismissed.
Patient satisfaction	Patient satisfaction was sometimes compromised due to the inequities experienced when seeking a diagnosis and accessing services.
Service avoidance	As a consequence of not receiving appropriate care when accessing services, PwRD reported avoiding health care services.
Variable care standards	Variable standards of care were experienced within the rare disease community in ways which were not just limited to the PROGRESS+ criteria. This included different standards of care experienced by people with different rare diseases.

Abbreviations: PwRD=people with a rare disease

The specific studies from Briscoe et al. from which data are derived for the mechanisms and outcomes described in Table 2 are presented in Table S1 and Table S2 in Additional File 1 – Tables of studies for toolkit [4]. Data from 77 of 96 UK based primary studies in the scoping review are represented in the toolkit; in addition, there are data from two systematic reviews of international evidence relating to maternity services.

Importantly, the patient care pathway depicted in the toolkit represents a dynamic interaction between the overarching context (health policy, resourcing and organisation), the health and social care contexts which are represented, and the social position and consequences of ill-health which are impacted by the mechanisms and outcomes. This is indicated by arrows which join the various contexts, and which move both backwards and forwards across the patient care pathway. We do not make recommendations in the toolkit for where interventions should be targeted to address the identified inequities, rather the toolkit is intended to facilitate this.

## Presentation of the toolkit

The toolkit is presented in a slide deck in Additional File 2: Toolkit for understanding where and how people with a rare disease experience health inequity across the patient care pathway in the UK. The slide deck format was chosen as a user-accessible medium which facilitated the use of hyperlinks, allowing users to interactively explore the content. The structure of the slide deck is presented in Table 3.

**Table 3:** Structure of toolkit in the MS PowerPoint file

Slide(s)	Content	Notes
1	Title slide	Authors’ institutional title slide with author list.
2	Instructions	Brief instructions on using the toolkit.
3	Patient pathway: receipt of a diagnosis	Depicts the contexts, mechanisms and outcomes associated with receipt of a diagnosis as a patient pathway, including detail about PROGRESS + sub-group characteristics.
4	Patient pathway: access to services	Depicts the contexts, mechanisms and outcomes associated with accessing services as patient pathway, including detail about PROGRESS + sub-group characteristics.
5–53	Elaboration on mechanisms and outcomes	Detailed descriptions of mechanisms and outcomes associated with the contexts depicted in the patient pathways on slides 3 and 4, including detail regarding PROGRESS+ characteristics of affected sub-groups. Slides 5–53 are accessed via hyperlinks in slides 3 and 4.

The toolkit is structured around a series of boxes which map onto health and social care services in the patient care pathway. The boxes representing service contexts are clear (i.e. not filled with colour) and labelled with the name of each service. Contexts relating to receipt of a diagnosis are presented in the first part of the toolkit (slide 3), and contexts representing access to services are presented in the second part of the toolkit (slide 4). *Health policy*,* resourcing and organisation* is represented as the overarching context in both parts of the toolkit. Mechanisms which lead to inequity within each context are presented in light shaded boxes, and outcomes arising from the mechanisms are presented in darker shaded boxes. Orange shading is used for the receipt of a diagnosis part of the toolkit, and blue shading is used for the access to services part of the toolkit. Each of the contexts, mechanisms and outcomes is assigned a number for ease of reference; however, the numbers do not necessarily indicate the order that someone moves through the pathway.

At the bottom of the toolkit is the exit point from the pathway (the individual/group level), which is labelled *social position and consequences of ill-health*. This feeds back into the service contexts.

Purple circular labels show which sub-groups are identified in the data as experiencing a particular type of mechanism or outcome. These labels correspond with the PROGRESS+ framework, including place of residence (P), race/ethnicity (R), gender/sex (G), socioeconomic status (S), age (A) and disability (D).

Solid lines with arrows show links between different contexts, and dotted lines with arrows show links between mechanisms and outcomes.

Hyperlinks within each mechanism and outcome label are linked to text on slides 5–53 which provide a brief descriptive summary of mechanisms and outcomes respectively, and more detail relating to specific sub-groups affected. Each of slides 5–53 includes a hyperlink which takes the user back to the relevant pathway, i.e. diagnosis or service access. Further instructions on how to use the toolkit are presented on the second slide of the slide deck.

## Future use of the toolkit

We anticipate that the toolkit can be used to raise awareness among clinicians, service providers, and policymakers of health inequities experienced by PwRD across the patient care pathway, and to guide policy entry points where targeted interventions can address these inequities. The toolkit has already been cited in the England Rare Disease Action Plan 2026, which mentions both the scoping review [4] and a pre-print version of the toolkit as contributing to the decision to include rare diseases in NHS England’s Core20PLUS5 framework. [8] As noted in Briscoe et al., the different types of identified health inequity are likely to be experienced with varying degrees of intensity for different conditions, which we were unable to draw out from the limited data available [4]. More research is needed to develop an understanding of how the experience of health inequities varies across different conditions, according to factors such as aetiology, prevalence and affected subgroups, across different stages of the pathway.

There are also some areas of service provision where access is limited more widely across the general population, such as mental health services; [9] and other disease areas, such as complex diseases more generally, which experience similar issues such as lack of care co-ordination [10]. Service providers and policymakers will need to exercise some judgement when thinking about where to target interventions which are specific to the rare disease community, and where there are experiences which need addressing more broadly. However, even within this larger picture of limited service provision, PwRD still experience unique challenges in accessing services which need to be acknowledged and addressed. There is, for example, a documented link between the issues which lead to misdiagnosis and diagnostic delay, and poor mental health [11]. As in many areas of health inequity, there is a complex picture of how health inequities intersect for PwRD which can only be addressed through sustained attention and a commitment to understanding. The extension of the UK Rare Disease Framework for another year until 2027 is a welcome development to this end [8].

## Electronic Supplementary Material

Below is the link to the electronic supplementary material.

Supplementary Material 1

Supplementary Material 2

## Data Availability

The datasets used and analysed during the current study are available from the corresponding author on reasonable request.

## Abbreviations

NIHR: National Institute for Health and Social Care Research
PROGRESS+: Place of residence, race/ethnicity, gender/sex, religion, education, socioeconomic status, social capital (+ age, disability)
PPI: Patient and public involvement
PwRD: People with a rare disease
UK: United Kingdom
