# Supplementary material for: Evidence of health inequities across the rare disease patient care pathway: development of a toolkit using a conceptual framework
Source: Orphanet J Rare Dis. 2026 Jun 2;21:213. doi: 10.1186/s13023-026-04389-0 (PMC13231698; doi:10.1186/s13023-026-04389-0)
Supplement: Supplementary file 1 — Supplementary Material 1 [file 13023_2026_4389_MOESM1_ESM.docx]

# Additional File 1: Tables of studies for toolkit

Table S1. Studies relating to contexts, mechanisms and outcomes for diagnosis of a rare disease

| **Label** (C=Context; M=Mechanism; O=Outcome) | **Mechanism/Outcome** | **Evidence shared across RD community** | **Evidence within specific groups in RD community (PROGRESS+)** | | | | | |
| --- | --- | --- | --- | --- | --- | --- | --- | --- |
|  |  |  | **Place of residence** | **Race/ethnicity** | **Gender** | **SES** | **Age** | **Disability** |
| *C1: Health policy, resourcing and organisation (wider context)* | | | | | | | | |
| M1 | Lack of care co-ordination | Franklish 2022^1^; Hagena 2024^2^ | - | - | - | - | Bell 2021^3^ | - |
| *C2: Primary care* | | | | | | | | |
| M2 | Lack of knowledge | Bennett 2021^4^; Daker-White 2013^5^; Flaherty 2024^6^; Limb 2010^7^; Muir 2016^8^; Pavey 2013^9^ | - | - | - | - | Simpson 2018^10^ | Shah 2014^11^ |
| M3 | Dismissive attitudes | Franklish 2022^1^; Limb 2010^7^; O’Brien 2011^12^; Pavey 2013^9^; Muir 2016^8^ | - | Franklish 2022^1^ | Bell 2021^3^; Franklish 2022^1^; Gilfillan 2024^13^ | - | Bell 2021^3^; Franklish 2022^1^; Simpson 2018^10^ | - |
| O1 | No/misdiagnosis | Bell 2021^3^; Dake-White 2013^5^; Franklish 2022^1^; Husson 2019^14^; McDonald 2019^15^; Morgan 2021^16^; Muir 2016^8^; Sharma 2020^17^ | - | - | - | - | Bell 2021^3^; McDonald 2019^15^ | - |
| O2 | Delayed diagnosis | Combs 2013^18^; Franklish 2022^1^; Genetic Alliance UK^19^; Hay 2021^20^; Husson 2019^14^; O’Brien 2011^12^ | - | - | - | - | Genetic Alliance Uk^19^ | Shah 2014^11^ |
| *C3: Secondary care (general hospital)* | | | | | | | | |
| M4 | Lack of knowledge | Bennett 2021^4^; Daker-White 2013^5^; Franklish 2022^1^; Muir 2016^8^; Twigg 2021^21^ | - | - | - | - | - | - |
| M5 | Dismissive attitudes | Bell 2021^3^ | - | - | - | - | Franklish 2022^1^; Bell 2021^3^ | Griffith 2011^23^ |
| M6 | Wrong specialist | Husson 2019^14^; Morgan 2021^16^ | - | - | - | - | - | - |
| M7 | Accessibility | Muir 2016^8^ | - | - | - | - | - | - |
| O3 | No/misdiagnosis | Muir 2016^8^; Twigg 2021^21^ | - | - | - | - | - | - |
| O4 | Delayed diagnosis | Husson 2019^14^ | - | - | - | - | - | - |
| *C4: Mental health services* | | | | | | | | |
| M8 | Accessibility | Franklish 2022^1^ | - | - | - | - | - | - |
| *C5: Genetic services* | | | | | | | | |
| M9 | Accessibility | Genetic Alliance 2023^19^ | Peter 2024^24^ | - | - | Peter 2024^24^ | - | - |
| M10 | Limited data sets | Church Smith n.d.^25^; Oerton 2011^26^; Wright 2022^27^ | - | Church Smith n.d.^25^; Oerton 2011^26^; Wright 2022^27^ | - | - | - | - |
| O5 | No diagnosis | Church Smith n.d.^25^; Oerton 2011^26^; Wright 2022^27^ | - | Church Smith n.d.^25^; Oerton 2011^26^; Wright 2022^27^ | - | - | - | - |
| *C6: Specialist services* | | | | | | | | |
| M11 | Wrong specialist | Muir 2016^8^ | - | - | - | - | - | - |
| M12 | Accessibility | Franklish 2022^1^; O'Brien 2023^28^ | Franklish 2022^1^ Peter 2024^24^ | - | - | Bell 2021^3^; Franklish 2022^1^; O’Brien 2011^12^; Pavey 2013^9^; Peter 2024^24^ | - | - |
| O6 | Variable care standards (within RD community and between RD/general population) | Franklish 2022^1^; O'Brien 2023^28^ | Franklish 2022^1^ Peter 2024^24^ | - | - | Bell 2021^3^; Franklish 2022^1^; O’Brien 2011^12^; Pavey 2013^9^; Peter 2024^24^ | - | - |
| *C7: RD diagnosis* | | | | | | | | |
| M13 | Lack of information | Costa 2022^29^; Crowe 2019 ^30^; Hassall 2022^31^; O’Brien 2023^28^ | - | - | - | - | - | - |
| O7 | Patient satisfaction | O’Brien 2023^28^ | - | - | - | - | - | - |
| O8 | Delayed diagnosis | Akanuwe 2020^32^; Al-Attar 2018^33^; Bell 2021^3^; Combs 2013^18^; Flaherty 2013^6^; Franklish 2022^1^; Genetic Alliance 2023^19^; Limb 2010^7^; Muir 2016^8^; O’Brien 2023^28^ | - | - | Song 2024^34^; Sanigorska 2022^22^* | Bell 2021^3^; Franlish 2022^1^; O’Brien 2011^12^; Pavey 2013^9^; Peter 2024^24^ | Genetic Alliance 2023^19^; Song 2024^34^ | - |
| *C8: SWAN diagnosis (see Table S2)* | | | | | | | | |

Abbreviations: RD=rare disease; SES=socioeconomic status; SWAN=syndrome without a name

Table S2. Studies relating to contexts, mechanisms and outcomes for accessing health services with a rare disease

| **Label** (C=Context; M=Mechanism; O=Outcome) | **Type of inequity** | **Evidence shared across RD community** | **Evidence within specific groups in RD community (PROGRESS)** | | | | | |
| --- | --- | --- | --- | --- | --- | --- | --- | --- |
|  |  |  | **Place of residence** | **Race/ethnicity** | **Gender** | **SES** | **Age** | **Disability** |
| *C7: Rare disease diagnosis (see Table S1)* | | | | | | | | |
| *C8: SWAN diagnosis* | | | | | | | | |
| M14 | Accessibility | Aldiss 2021^35^ | - | Church Smith n.d.^25^; Oerton 2011^26^; Wright 2022^27^ | - | - | Aldiss 2021^35^ | - |
| *C9: Health policy, resourcing and organisation* | | | | | | | | |
| M15 | Lack of care co-ordination | Aldiss 2021^35^; Aljuburi 2012^36^; Bell 2021^3^; Cassidy 2023^37^; Franklish 2022^1^; Genetic Alliance UK 2023^19^; Grose 2014^38^; Harris 2018^39^; Husson 2019^14^; Limb 2010^7^; Morris 2022^40^; Multiple System Atrophy 2022^41^; Muir 2016^8^; Pinto 2021^42^; Spencer-Tansley 2018^43^; Watson 2023^44^ | - | - | - | - | Aldiss 2021^35^; Bell 2021^3^; Genetic Alliance UK 2023^19^; Haig-Ferguson^45^; Limb 2010^7^; Muir 2016^8^; Spencer Tansley 2018^43^ | - |
| *C10: Social care* | | | | | | | | |
| M16 | Lack of knowledge | Griffith 2011^23^; O’Brien 2012^46^; O’Brien 2011^47^; Skirton 2010^48^ | - | - | - | - | - | - |
| M17 | Dismissive attitudes | Franklish 2022^1^ | - | - | - | - | - | - |
| M18 | Accessibility | Cohen 2017^49^; Crowe 2019^30^; McDonald 2019^15^ | - | - | - | - | - | - |
| *C11: Emergency services* | | | | | | | | |
| M19 | Lack of knowledge | Chakravorty 2018^50^ | - | - | - | - | - | - |
| M20 | Dismissive attitudes | Renedo 2019^51^ | - | Renedo 2019^51^ | - | - | Renedo 2019^51^ | - |
| O9 | Service avoidance | Chakravorty 2018^50^; Renedo 2019^51^ | - | - | - | - | - | - |
| *C12: Primary dental care* | | | | | | | | |
| M21 | Lack of knowledge | Kalsi 2012^52^ | - | - | - | - | - | - |
| M22 | Accessibility | Booth 2023^53^; Kalsi 2012^52^ | - | - | - | - | - | - |
| *C13: Primary care (GP surgery)* | | | | | | | | |
| M23 | Lack of knowledge | Cassidy 2023^37^; Daker-White 2013^5^; Franklish 2022^1^; Vallortigara 2023^54^; Wray 2021^55^ | - | - | - | - | Wray 2021^55^ | - |
| M24 | Dismissive attitudes | Dures 2011^56^; Franklish 2022^1^ | - | - | Whitaker 2021^57^ | - | - | - |
| M25 | Lack of information | Aldiss 2021^35^ Cassidy^37^; Neelamekam 2017^58^; O’Brien 2012^46^ | - | - | Taylor 2014^59^ | - | Aldiss 2021^35^ | - |
| O10 | Not listened to | Franklish 2022^1^ | - | - | - | - | - | - |
| *C14: Secondary care (hospital)* | | | | | | | | |
| M26 | Lack of knowledge | Aubeeluck 2012^60^; Bell 2021^3^; Bennett 2021^4^; Berghs 2022^61^; Cassidy 2023^37^; Cunniff 2015^62^; Griffith 2011^23^; Husson 2019^14^; Khair 2019^63^; McMullan 2022^64^; Morgan 2021 ^16^; Neelamekam 2017^58^; O’Brien 2015^65^; Simpson 2018^10^; Skirton 2010^48^; Spencer-Tansley 2018^43^ | - | - | - | - | - | - |
| M27 | Lack of information | Chakravorty 2018^50^; Fixter 2017^66^; O’Brien 2012^46^; Pavey 2013^9^ | - | - | - | - | Aldiss 2021^35^ | - |
| M28 | Dismissive attitudes | Dures 2011^56^; Miles 2019^67^; Vasilica 2021^68^ | - | - | - | - | - | - |
| M29 | Accessibility | Aldiss 2021^35^; Daker-White 2013^5^ | - | - | - | Bell 2021^3^; Morris 2022^40^; Khair 2019^63^; Skirton 2010^48^ | - | O’Brien 2015^65^; Multiple System Atrophy Trust 2022^41^ |
| O11 | Variable care standards (within RD community and between RD/general population) | Aldiss 2021^35^; Daker-White 2013^5^; Muir 2016^8^ | - | - | - | Bell 2021^3^; Morris 2022^40^; Khair 2019^63^; Skirton 2010^48^ | - | O’Brien 2015^65^; Multiple System Atrophy Trust, 2022^41^ |
| O12 | Not listened to | Berghs 2024^69^; Miles 2019^67^; Morgan 2021^16^; Neelamekam 2017^58^; Vasilica 2021^68^ | - | - | - | - | - | - |
| *C15: Mental health services* | | | | | | | | |
| M30 | Accessibility | Cassidy 2023^37^; Chakravorty 2018^50^; Multiple System Atrophy Trust 2019^70^; Multiple System Atrophy Trust^41^; O’Brien 2012^71^; Trimmer 2024^72^; Limb 2010^7^ | - | - | - | - | - | - |
| M31 | Dismissive attitudes | Bennett 2021^4^; Berghs 2024^69^; Berghs 2022^61^; Dures 2011^56^; Franklish 2022^1^; Whitehead 2012^73^; Multiple System Atrophy Trust 2022^41^ | - | - | - | - | - | - |
| *C16: Maternity care* | | | | | | | | |
| M32 | Lack of knowledge | Anderson 2021^74^* | - | - | Anderson 2021^74^*; Rance 2019^75^ | - | - | - |
| *C17: Specialist services* | | | | | | | | |
| M33 | Accessibility | Franklish 2022^1^; Morris 2022^40^; Morris 2023^76^; Muir 2016^8^; Vallortigara 2023^54^ | Aldiss 2021^35^; Grose 2014^38^; O’Brien 2011^47^; Simpson 2018^10^; Specialist Healthcare Alliance 2023^77^; Spencer-Tansley 2018^43^ | - | - | Bell 2021^3^; Morris 2022^40^; Khair 2019^63^; Skirton 2010^48^ | - | - |
| M34 | Lack of knowledge | Cammidge 2016^78^ | - | - | Cammidge 2016^78^ | - | - | - |
| M35 | Lack of information | Rodger 2015^79^** | - | - | Cammidge 2016^78^; Kahir 2019^63^ | - | Kahir 2019^63^ | - |
| O13 | Variable care standards (focus is within RD community) | Cammidge 2016^78^; Rodger 2015 ^79^ | - | - | Cammidge 2016^78^; Kahir 2019^63^ | - | - | - |
| C18: End of life care | | | | | | | | |
| M36 | Lack of information | Whitehead 2012^73^ | - | - | - | - | - | - |

Abbreviations: RD=rare disease; SES=socioeconomic status; SWAN=syndrome without a name
*Evidence from non-UK source (systematic review with international evidence)
**People who can’t access specialist centres are less satisfied with information provision than those who can access specialist centres.

# References

1. Franklish N, Genetic Alliance UK. Good diagnosis: improving the experiences of a diagnosis for people with rare conditions. London; 2022.

2. Hagena A, Stananought N, Greene M, et al. Motor neurone disease: What are the support needs of patients and carers? European Journal of Palliative Care. 2014;21:232-5.

3. Bell L, Pearce G. Parents’ experiences of children’s health care for hypermobile Ehlers–Danlos syndrome and hypermobility spectrum disorders. Children's Health Care. 2021;51(1):37-61.

4. Bennett SE, Walsh N, Moss T, Palmer S. Understanding the psychosocial impact of joint hypermobility syndrome and Ehlers-Danlos syndrome hypermobility type: a qualitative interview study. Disabil Rehabil. 2021;43(6):795-804.

5. Daker-White G, Ealing J, Greenfield J, et al. Trouble with ataxia: A longitudinal qualitative study of the diagnosis and medical management of a group of rare, progressive neurological conditions. SAGE Open Med. 2013;1:2050312113505560.

6. Flaherty CC, Phillips IR, Janmohamed A, Shephard EA. Living with trimethylaminuria and body and breath malodour: personal perspectives. BMC Public Health. 2024;24(1):222.

7. Limb L, Nutt S, Sen A. Experiences of rare diseases: an insight from parents and families. Rare Disease Uk: London; 2010.

8. Muir E. The Rare Reality - an insight into the patient and family experience of rare disease. London: Rare Disease UK; 2016.

9. Pavey A, Allen-Collinson J, Pavey T. The Lived Experience of Diagnosis Delivery in Motor Neurone Disease: A Sociological-Phenomenological Study. Sociological Research Online. 2013;18(2):36-47.

10. Simpson A, Ross R, Porter J, et al. Adrenal Insufficiency in Young Children: a Mixed Methods Study of Parents' Experiences. J Genet Couns. 2018;27(6):1447-58.

11. Shah S. Barriers and facilitators to health and social care for BME service users and carers with rare neurodegenerative conditions 2014.

12. O'Brien MR, Whitehead B, Jack BA, Mitchell JD. From symptom onset to a diagnosis of amyotrophic lateral sclerosis/motor neuron disease (ALS/MND): experiences of people with ALS/MND and family carers - a qualitative study. Amyotroph Lateral Scler. 2011;12(2):97-104.

13. Gilfillan R, Carter P. Issues of identity, perceptions and isolation: An interpretative phenomenological analysis of women's experience of Mayer-Rokitansky-Kuster-Hauser (MRKH) syndrome. J Health Psychol. 2024;29(3):200-12.

14. Husson O, Younger E, Dunlop A, et al. Desmoid fibromatosis through the patients' eyes: time to change the focus and organisation of care? Support Care Cancer. 2019;27(3):965-80.

15. McDonald A, Goodwin J, Roberts S, et al. 'We've made the best of it. But we do not have a normal life': families' experiences of tuberous sclerosis complex and seizure management. J Intellect Disabil Res. 2019;63(8):947-56.

16. Morgan A, Eccles FJR, Greasley P. Experiences of living with dystonia. Disabil Rehabil. 2021;43(7):944-52.

17. Sharma N. Living with cystic fibrosis: Patients' experiences of diagnosis in adulthood. Bristol: University of the West of England; 2020.

18. Combs R, Hall G, Payne K, et al. Understanding the expectations of patients with inherited retinal dystrophies. Br J Ophthalmol. 2013;97(8):1057-61.

19. Genetic Alliance UK. Coordinating care: learning from the experiences of people living with rare conditions. Genetic Alliance UK; 2023.

20. Hay E, Elmslie F, Lanyon P, Cole T. The Diagnostic Odyssey in rare diseases; a Task and Finish Group report for the Department of Health and Social Care. Department of Health and Social Care; 2021.

21. Twigg J, Methley A, Lavin T, et al. Living with Polyneuropathy Organomegaly Endocrinopathy Monoclonal gammopathy Skin changes (POEMS) syndrome: a case study of healthcare experiences and quality of life. Disabil Rehabil. 2021;43(17):2502-10.

22. Sanigorska A, Chaplin S, Holland M, et al. The lived experience of women with a bleeding disorder: A systematic review. Res Pract Thromb Haemost. 2022;6(1):e12652.

23. Griffith GM, Hastings RP, Nash S, et al. "You have to sit and explain it all, and explain yourself." Mothers' experiences of support services for their offspring with a rare genetic intellectual disability syndrome. J Genet Couns. 2011;20(2):165-77.

24. Peter M, Hill M, Fisher J, et al. Equity and timeliness as factors in the effectiveness of an ethical prenatal sequencing service: reflections from parents and professionals. Eur J Hum Genet. 2024.

25. Church Smith CL, Roy A, Steeds S, et al. Underdiagnosis of Fabry Disease in minority ethnic groups: the role of health inequalities. [Manuscript submitted for publication]. n.d.

26. Oerton J, Khalid JM, Besley G, et al. Newborn screening for medium chain acyl-CoA dehydrogenase deficiency in England: prevalence, predictive value and test validity based on 1.5 million screened babies. J Med Screen. 2011;18(4):173-81.

27. Wright CF, Campbell P, Eberhardt RY, et al. Optimising diagnostic yield in highly penetrant genomic disease. medRxiv. 2022:2022.07.25.22278008.

28. O'Brien MR, McDermott C, Aoun S, et al. The diagnostic experience for people with MND and their caregivers in the U.K. J Neurol Sci. 2023;444:120483.

29. Costa A, Frankova V, Robert G, et al. Co-designing models for the communication of genomic results for rare diseases: a comparative study in the Czech Republic and the United Kingdom. J Community Genet. 2022;13(3):313-27.

30. Crowe AL, McKnight AJ, McAneney H. Communication Needs for Individuals With Rare Diseases Within and Around the Healthcare System of Northern Ireland. Front Public Health. 2019;7:236.

31. Hassall S, Smith DM, Rust S, et al. "Why them, why me, why us?" The experiences of parents of children with lysosomal acid lipase deficiency: an interpretative phenomenological analysis study. Orphanet J Rare Dis. 2022;17(1):193.

32. Akanuwe JNA, Laparidou D, Curtis F, et al. Exploring the experiences of having Guillain-Barre Syndrome: A qualitative interview study. Health Expect. 2020;23(5):1338-49.

33. Al-Attar M. TRAPPED - an insight into two sisters' struggle to access treatment for a rare genetic disease. Orphanet J Rare Dis. 2018;13(1):37.

34. Song K, Shrestha R, Delaney H, et al. Diagnostic journey for individuals with fibrous dysplasia / McCune albright syndrome (FD/MAS). Orphanet J Rare Dis. 2024;19(1):50.

35. Aldiss S, Gibson F, Geoghegan S, et al. 'We don't know what tomorrow will bring': Parents' experiences of caring for a child with an undiagnosed genetic condition. Child Care Health Dev. 2021;47(5):588-96.

36. Aljuburi G, Phekoo KJ, Okoye NO, et al. Patients' views on improving sickle cell disease management in primary care: focus group discussion. JRSM Short Rep. 2012;3(12):84.

37. Cassidy S, Evans S, Pinto A, et al. Parent's Perception of the Types of Support Given to Families with an Infant with Phenylketonuria. Nutrients. 2023;15(10).

38. Grose J, Freeman J, Marsden J. Service delivery for people with hereditary spastic paraparesis living in the South West of England. Disabil Rehabil. 2014;36(11):907-13.

39. Harris DA, Jack K, Wibberley C. The meaning of living with uncertainty for people with motor neurone disease. J Clin Nurs. 2018;27(9-10):2062-71.

40. Morris S, Hudson E, Bloom L, et al. Co-ordinated care for people affected by rare diseases: the CONCORD mixed-methods study. Health and Social Care Delivery Research. 2022;10(5):1-220.

41. Multiple System Atrophy Trust. 2022 MSA Trust Needs Survey. Chesterfield, Derbyshire; 2022.

42. Pinto C, Geraghty AWA, Yardley L, Dennison L. Emotional distress and well-being among people with motor neurone disease (MND) and their family caregivers: a qualitative interview study. BMJ Open. 2021;11(8):e044724.

43. Spencer-Tansley R. Understanding children and young people’s experience. London: Rare Disease UK; 2018.

44. Walton H, Ng PL, Simpson A, et al. Experiences of coordinated care for people in the UK affected by rare diseases: cross-sectional survey of patients, carers, and healthcare professionals. Orphanet J Rare Dis. 2023;18(1):364.

45. Haig-Ferguson A, Wallace V, Davis C. The lived experience of adults and parents: Transitioning from paediatric to adult health care with oesophageal atresia and tracheo-oesophageal fistula. J Clin Nurs. 2023;32(7-8):1433-42.

46. O'Brien MR, Whitehead B, Murphy PN, et al. Social services homecare for people with motor neurone disease/amyotrophic lateral sclerosis: why are such services used or refused? Palliat Med. 2012;26(2):123-31.

47. O’Brien M, Whitehead B, Jack B, Mitchell JD. Multidisciplinary team working in motor neurone disease: patient and family carer views. British Journal of Neuroscience Nursing. 2011;7(4):580-5.

48. Skirton H, Williams JK, Jackson Barnette J, Paulsen JS. Huntington disease: families' experiences of healthcare services. J Adv Nurs. 2010;66(3):500-10.

49. Cohen W, McCartney E, Crampin L. 22q11 deletion syndrome: Parents' and children's experiences of educational and healthcare provision in the United Kingdom. J Child Health Care. 2017;21(2):142-52.

50. Chakravorty S, Tallett A, Witwicki C, et al. Patient-reported experience measure in sickle cell disease. Arch Dis Child. 2018;103(12):1104-9.

51. Renedo A, Miles S, Chakravorty S, et al. Not being heard: barriers to high quality unplanned hospital care during young people's transition to adult services - evidence from 'this sickle cell life' research. BMC Health Serv Res. 2019;19(1):876.

52. Kalsi H, Nanayakkara L, Pasi KJ, et al. Access to primary dental care for patients with inherited bleeding disorders. Haemophilia. 2012;18(4):510-5.

53. Booth AJ, Cavell T, Gill T, et al. A cohort study and matched pair analysis evaluating the effects of the COVID-19 pandemic on access to dental care for people with inherited bleeding disorders. Haemophilia. 2023;29(5):1276-82.

54. Vallortigara J, Greenfield J, Hunt B, et al. Patient pathways for rare diseases in Europe: ataxia as an example. Orphanet J Rare Dis. 2023;18(1):328.

55. Wray J, Sugarman H, Davis L, et al. Improving community-based care for children with a rare condition: The example of long-segment congenital tracheal stenosis and perceptions of health professionals, parents and teachers. Int J Pediatr Otorhinolaryngol. 2021;143:110651.

56. Dures E, Morris M, Gleeson K, Rumsey N. The psychosocial impact of epidermolysis bullosa. Qual Health Res. 2011;21(6):771-82.

57. Whitaker S, Aiston H, Hung WT, et al. Haemophilia Carriers Experience Study (CARES): A mixed method exploration into the experience of women who are carriers of Haemophilia. Haemophilia. 2021;27(5):848-53.

58. Neelamekam S, Kwok S, Malone R, et al. The impact of lipoprotein lipase deficiency on health-related quality of life: a detailed, structured, qualitative study. Orphanet J Rare Dis. 2017;12(1):156.

59. Taylor B. Sexuality, intimacy and motor neurone disease: matters of concern. British Journal of Neuroscience Nursing. 2014;10(5):242-51.

60. Aubeeluck AV, Buchanan H, Stupple EJ. 'All the burden on all the carers': exploring quality of life with family caregivers of Huntington's disease patients. Qual Life Res. 2012;21(8):1425-35.

61. Berghs MJ, Horne F, Yates S, et al. Black sickle cell patients' lives matter: healthcare, long-term shielding and psychological distress during a racialised pandemic in England - a mixed-methods study. BMJ Open. 2022;12(9):e057141.

62. Cunniff A, Chisholm V, Chouliara Z. Listening to fathers of sons with duchenne muscular dystrophy. New Male Studies. 2015;4(2):5-23.

63. Khair K, Pelentsov L. Assessing the supportive care needs of parents with a child with a bleeding disorder using the Parental Needs Scale for Rare Diseases (PNS-RD): A single-centre pilot study. Haemophilia. 2019;25(5):831-7.

64. McMullan J, Crowe AL, Downes K, et al. Carer reported experiences: Supporting someone with a rare disease. Health Soc Care Community. 2022;30(3):1097-108.

65. O'Brien MR, Preston H. Family carer perspectives of acute hospital care following a diagnosis of motor neuron disease: a qualitative secondary analysis. BMJ Support Palliat Care. 2015;5(5):503-9.

66. Fixter V, Butler C, Daniels J, Phillips S. A Qualitative Analysis of the Information Needs of Parents of Children with Cystic Fibrosis prior to First Admission. J Pediatr Nurs. 2017;34:e29-e33.

67. Miles S, Renedo A, Augustine C, et al. Obstacles to use of patient expertise to improve care: a co-produced longitudinal study of the experiences of young people with sickle cell disease in non-specialist hospital settings. Critical Public Health. 2019;30(5):544-54.

68. Vasilica C, Oates T, Clausner C, et al. Identifying Information Needs of Patients With IgA Nephropathy Using an Innovative Social Media-stepped Analytical Approach. Kidney Int Rep. 2021;6(5):1317-25.

69. Berghs M, Horne F, Yates S, et al. The indignities of shielding during the COVID-19 pandemic for people with sickle cell disorders: an interpretative phenomenological analysis. Front Sociol. 2024;9:1334633.

70. Multiple System Atrophy Trust. People with MSA Needs Survey 2019: Technical report. Chesterfield, Derbyshire; 2019.

71. O'Brien MR, Whitehead B, Jack BA, Mitchell JD. The need for support services for family carers of people with motor neurone disease (MND): views of current and former family caregivers a qualitative study. Disabil Rehabil. 2012;34(3):247-56.

72. Trimmer RE, Mandy WPL, Muntoni F, Maresh KE. Understanding anxiety experienced by young males with Duchenne muscular dystrophy: a qualitative focus group study. Neuromuscul Disord. 2024;34:95-104.

73. Whitehead B, O'Brien MR, Jack BA, Mitchell D. Experiences of dying, death and bereavement in motor neurone disease: a qualitative study. Palliat Med. 2012;26(4):368-78.

74. Anderson LK, Lane KR. The diagnostic journey in adults with hypermobile Ehlers-Danlos syndrome and hypermobility spectrum disorders. J Am Assoc Nurse Pract. 2021;34(4):639-48.

75. Rance JC, Skirton H. An integrative review of factors that influence reproductive decisions in women with sickle cell disease. J Community Genet. 2019;10(2):161-9.

76. Morris S, Vallortigara J, Greenfield J, et al. Impact of specialist ataxia centres on health service resource utilisation and costs across Europe: cross-sectional survey. Orphanet J Rare Dis. 2023;18(1):382.

77. Specialised Healthcare Alliance. Rare diseases, common inequalities: Briding rare disease into the health inequalities agenda. 2023.

78. Cammidge SL, Duff AJ, Latchford GJ, Etherington C. When Women with Cystic Fibrosis Become Mothers: Psychosocial Impact and Adjustments. Pulm Med. 2016;2016:9458980.

79. Rodger S, Woods KL, Bladen CL, et al. Adult care for Duchenne muscular dystrophy in the UK. J Neurol. 2015;262(3):629-41.
