# Supplementary material for: Evidence of health inequities across the rare disease patient care pathway: development of a toolkit using a conceptual framework
Source: Orphanet J Rare Dis. 2026 Jun 2;21:213. doi: 10.1186/s13023-026-04389-0 (PMC13231698; doi:10.1186/s13023-026-04389-0)
Supplement: Supplementary file 2 — Supplementary Material 2 [file 13023_2026_4389_MOESM2_ESM.pptx]

## Slide 1
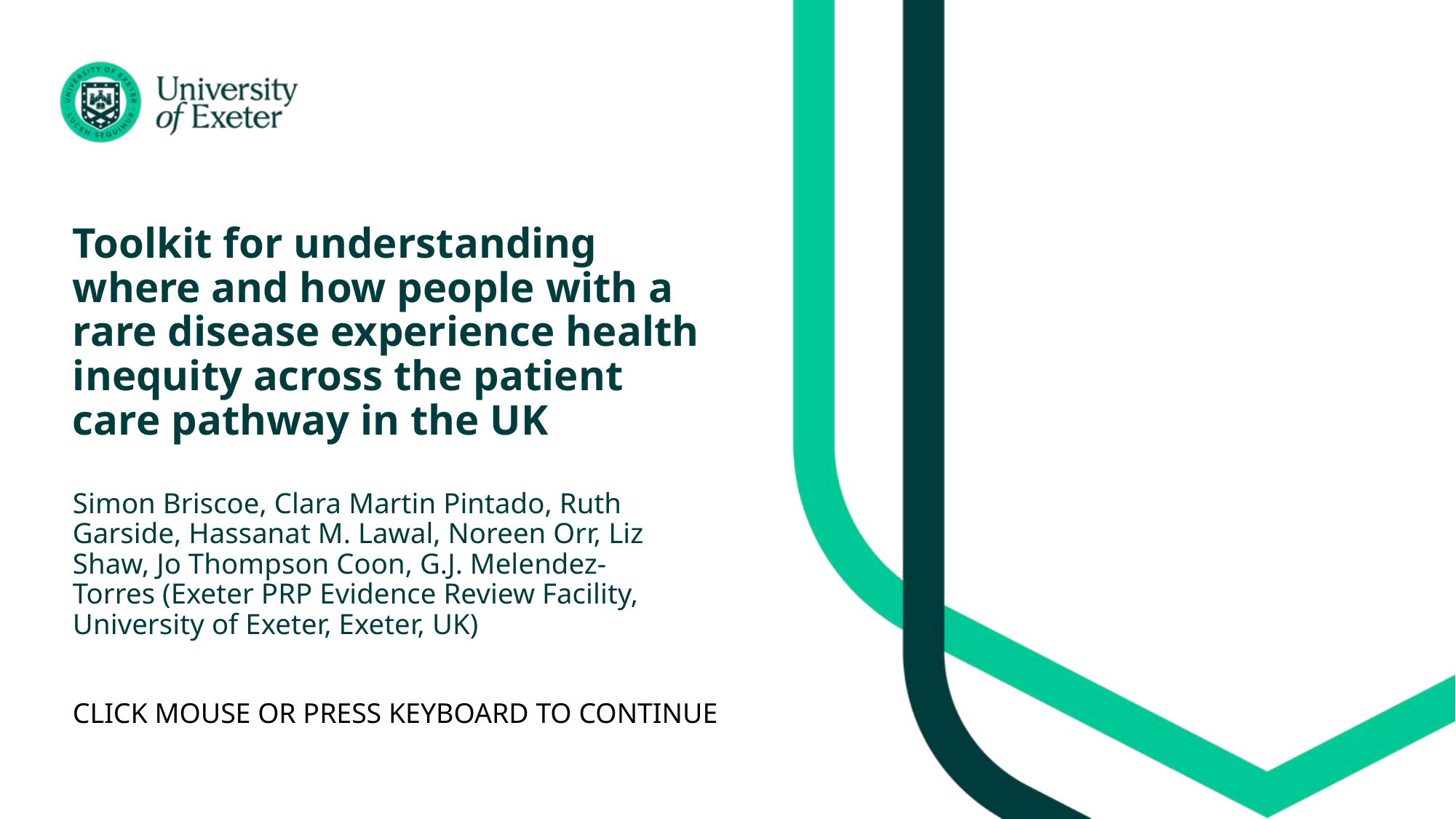

# Toolkit for understanding where and how people with a rare disease experience health inequity across the patient care pathway in the UK
Simon Briscoe, Clara Martin Pintado, Ruth Garside, Hassanat M. Lawal, Noreen Orr, Liz Shaw, Jo Thompson Coon, G.J. Melendez-Torres (Exeter PRP Evidence Review Facility, University of Exeter, Exeter, UK)
CLICK MOUSE OR PRESS KEYBOARD TO CONTINUE

## Slide 2
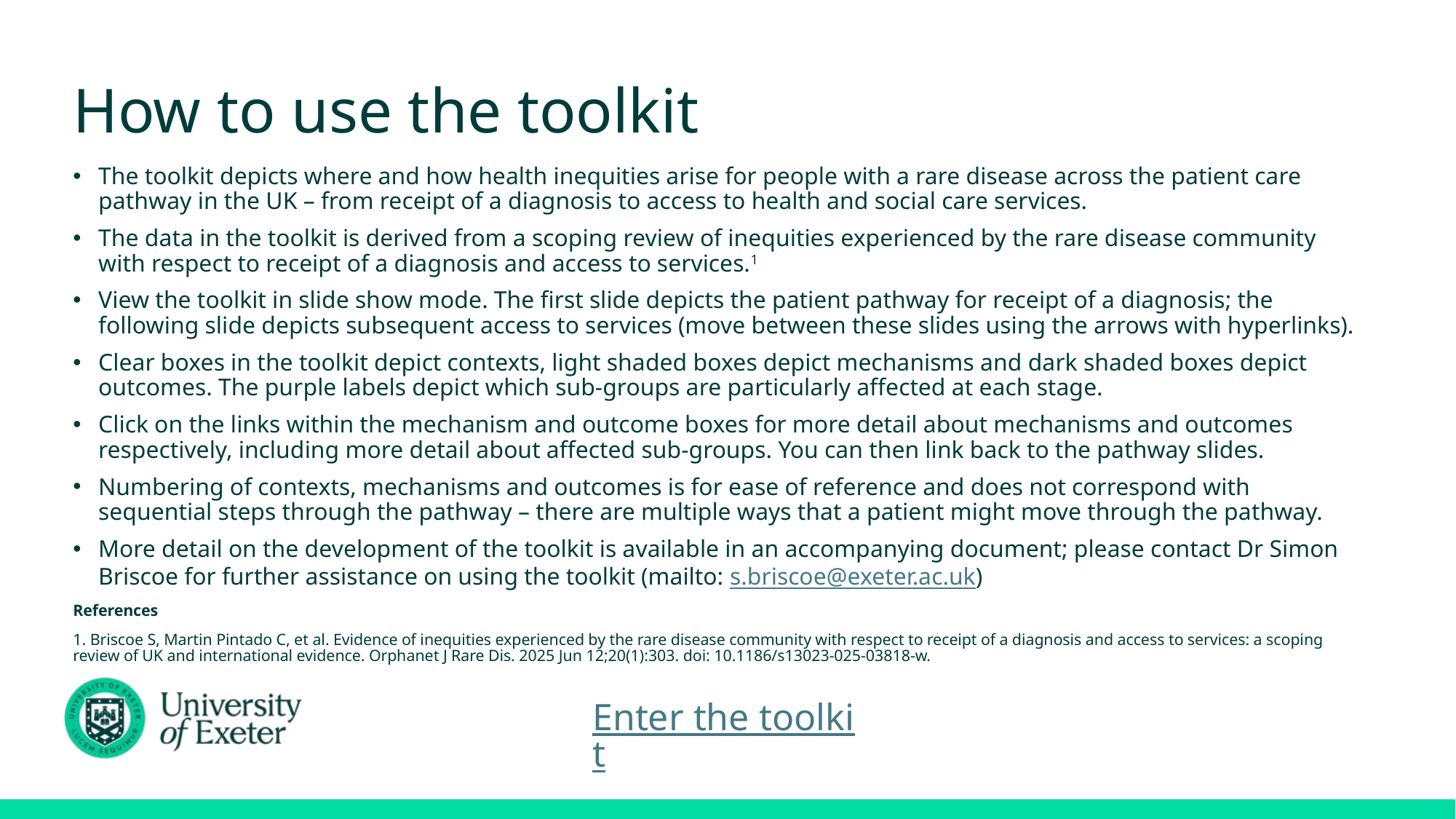

# How to use the toolkit
The toolkit depicts where and how health inequities arise for people with a rare disease across the patient care pathway in the UK – from receipt of a diagnosis to access to health and social care services.
The data in the toolkit is derived from a scoping review of inequities experienced by the rare disease community with respect to receipt of a diagnosis and access to services.1
View the toolkit in slide show mode. The first slide depicts the patient pathway for receipt of a diagnosis; the following slide depicts subsequent access to services (move between these slides using the arrows with hyperlinks).
Clear boxes in the toolkit depict contexts, light shaded boxes depict mechanisms and dark shaded boxes depict outcomes. The purple labels depict which sub-groups are particularly affected at each stage.
Click on the links within the mechanism and outcome boxes for more detail about mechanisms and outcomes respectively, including more detail about affected sub-groups. You can then link back to the pathway slides.
Numbering of contexts, mechanisms and outcomes is for ease of reference and does not correspond with sequential steps through the pathway – there are multiple ways that a patient might move through the pathway.
More detail on the development of the toolkit is available in an accompanying document; please contact Dr Simon Briscoe for further assistance on using the toolkit (mailto: s.briscoe@exeter.ac.uk)
References
1. Briscoe S, Martin Pintado C, et al. Evidence of inequities experienced by the rare disease community with respect to receipt of a diagnosis and access to services: a scoping review of UK and international evidence. Orphanet J Rare Dis. 2025 Jun 12;20(1):303. doi: 10.1186/s13023-025-03818-w.
Enter the toolkit

## Slide 3
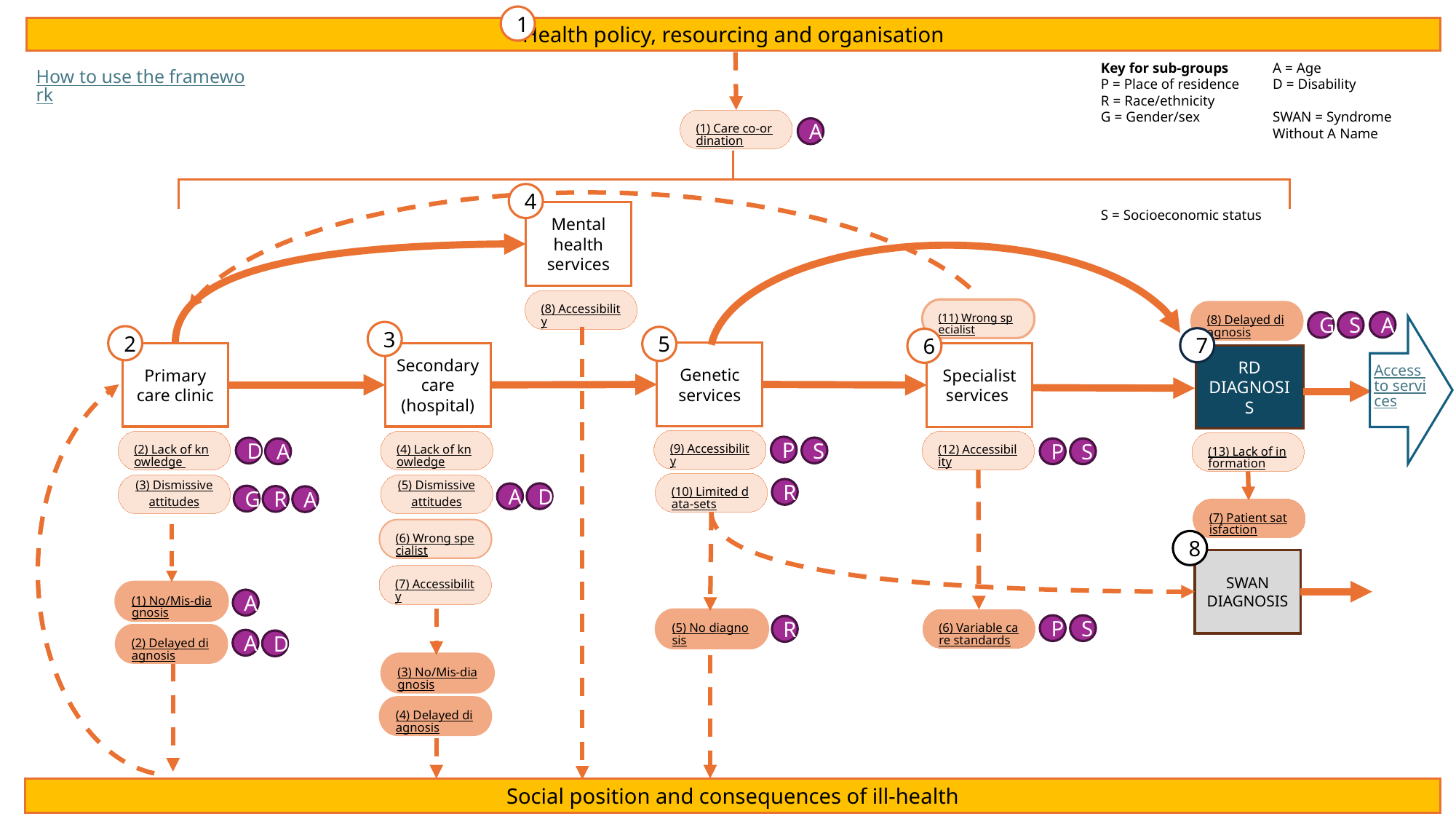

1
Health policy, resourcing and organisation
Key for sub-groups
P = Place of residence
R = Race/ethnicity
G = Gender/sex
S = Socioeconomic status
A = Age
D = Disability
SWAN = Syndrome Without A Name
How to use the framework
(1) Care co-ordination
A
4
Mental health services
(8) Accessibility
(11) Wrong specialist
(8) Delayed diagnosis
A
G
S
3
2
5
7
6
Genetic services
Primary care clinic
Secondary care (hospital)
Specialist services
RD DIAGNOSIS
Access to services
(9) Accessibility
(2) Lack of knowledge
(4) Lack of knowledge
(12) Accessibility
(13) Lack of information
P
S
D
A
P
S
(10) Limited data-sets
(5) Dismissive
attitudes
(3) Dismissive
attitudes
R
D
A
G
R
A
(7) Patient satisfaction
(6) Wrong specialist
8
SWAN DIAGNOSIS
(7) Accessibility
(1) No/Mis-diagnosis
A
(6) Variable care standards
(5) No diagnosis
P
S
R
(2) Delayed diagnosis
A
D
(3) No/Mis-diagnosis
(4) Delayed diagnosis
Social position and consequences of ill-health

## Slide 4
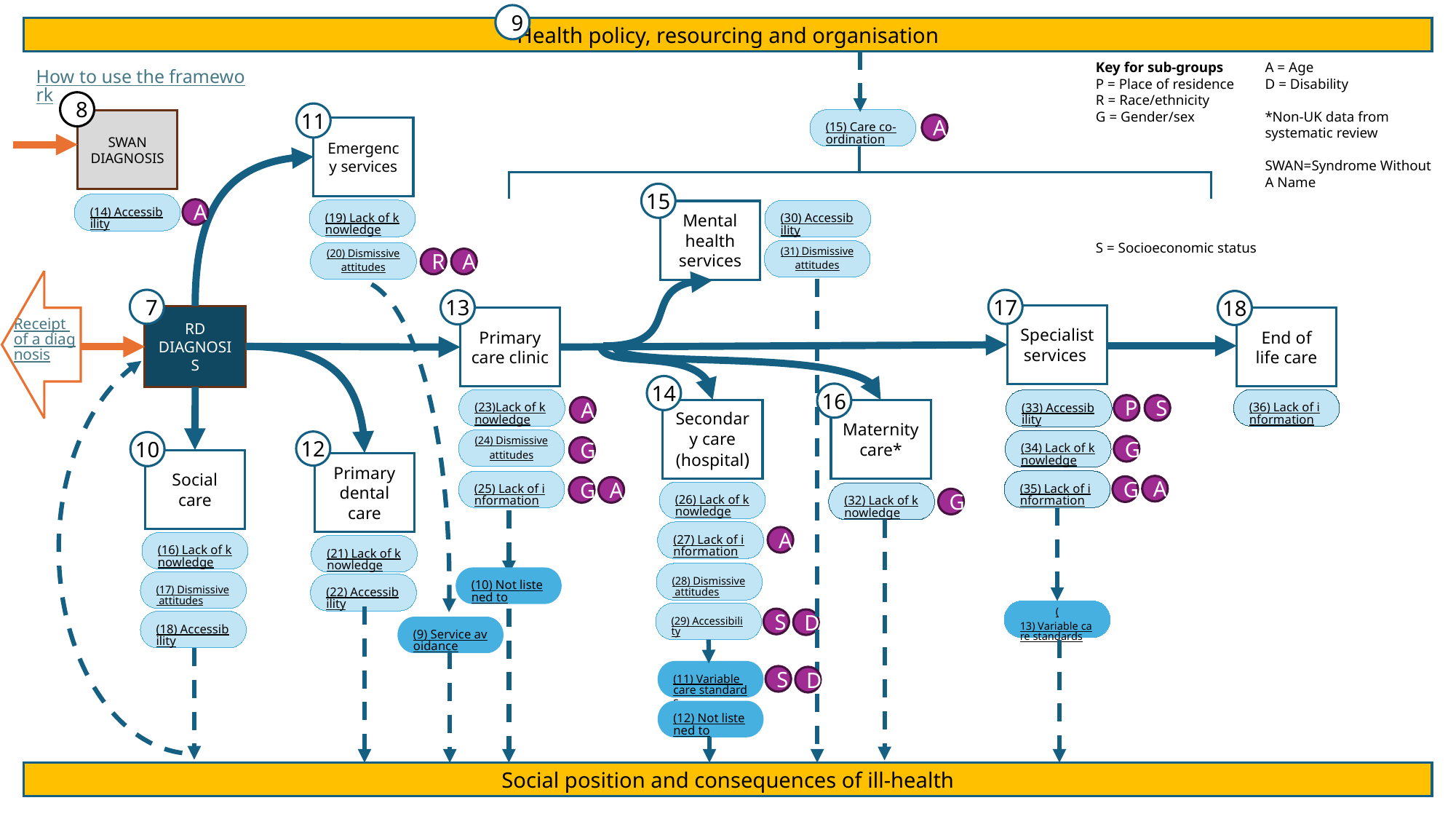

9
Health policy, resourcing and organisation
Key for sub-groups
P = Place of residence
R = Race/ethnicity
G = Gender/sex
S = Socioeconomic status
A = Age
D = Disability
*Non-UK data from systematic review
SWAN=Syndrome Without A Name
How to use the framework
8
11
(15) Care co-ordination
SWAN DIAGNOSIS
A
Emergency services
15
(14) Accessibility
A
(19) Lack of knowledge
(30) Accessibility
Mental health services
(31) Dismissive
attitudes
(20) Dismissive
attitudes
A
R
7
17
13
18
Specialist services
RD DIAGNOSIS
End of life care
Primary care clinic
Receipt of a diagnosis
14
16
(23)Lack of knowledge
(36) Lack of information
(33) Accessibility
S
P
A
Secondary care (hospital)
Maternity care*
(24) Dismissive
attitudes
(34) Lack of knowledge
12
10
G
G
Social care
Primary dental care
(35) Lack of information
(25) Lack of information
A
G
G
A
(26) Lack of knowledge
(32) Lack of knowledge
G
(27) Lack of information
A
(16) Lack of knowledge
(21) Lack of knowledge
(28) Dismissive attitudes
(10) Not listened to
(17) Dismissive attitudes
(22) Accessibility
(13) Variable care standards
(29) Accessibility
S
D
(18) Accessibility
(9) Service avoidance
(11) Variable care standards
S
D
(12) Not listened to
Social position and consequences of ill-health

## Slide 5
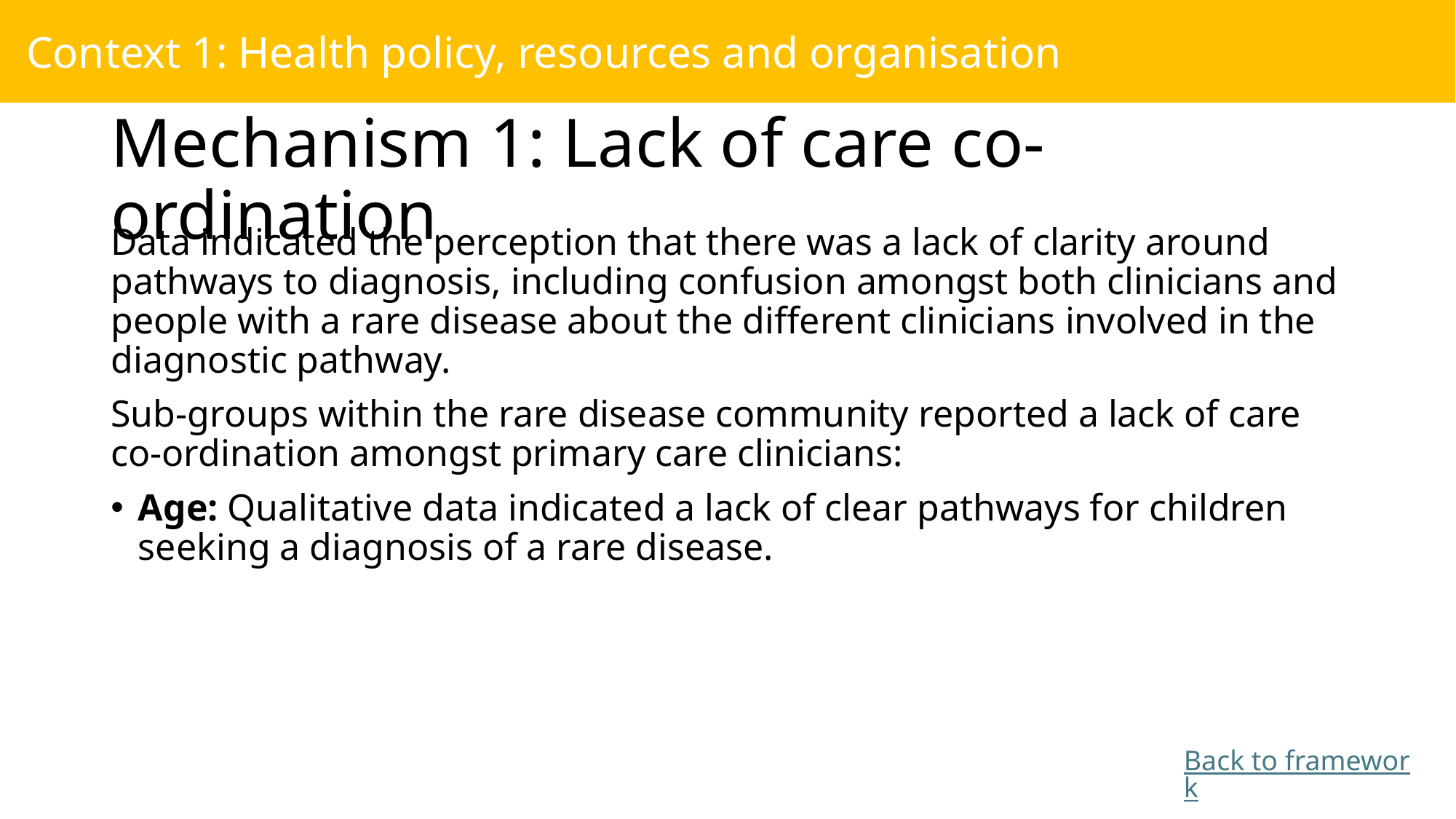

Context 1: Health policy, resources and organisation
# Mechanism 1: Lack of care co-ordination
Data indicated the perception that there was a lack of clarity around pathways to diagnosis, including confusion amongst both clinicians and people with a rare disease about the different clinicians involved in the diagnostic pathway.
Sub-groups within the rare disease community reported a lack of care co-ordination amongst primary care clinicians:
Age: Qualitative data indicated a lack of clear pathways for children seeking a diagnosis of a rare disease.
Back to framework

## Slide 6
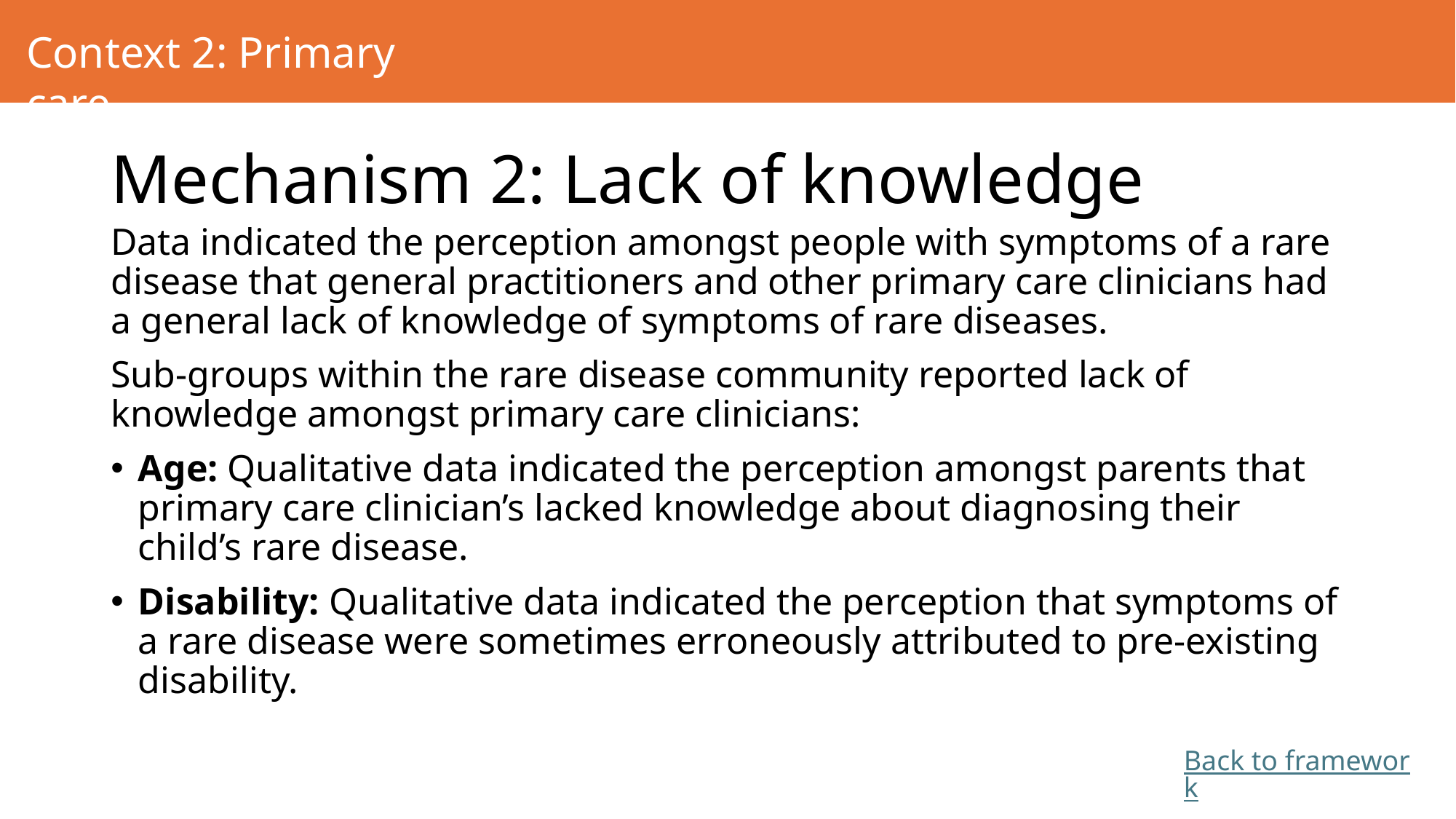

Context 2: Primary care
# Mechanism 2: Lack of knowledge
Data indicated the perception amongst people with symptoms of a rare disease that general practitioners and other primary care clinicians had a general lack of knowledge of symptoms of rare diseases.
Sub-groups within the rare disease community reported lack of knowledge amongst primary care clinicians:
Age: Qualitative data indicated the perception amongst parents that primary care clinician’s lacked knowledge about diagnosing their child’s rare disease.
Disability: Qualitative data indicated the perception that symptoms of a rare disease were sometimes erroneously attributed to pre-existing disability.
Back to framework

## Slide 7
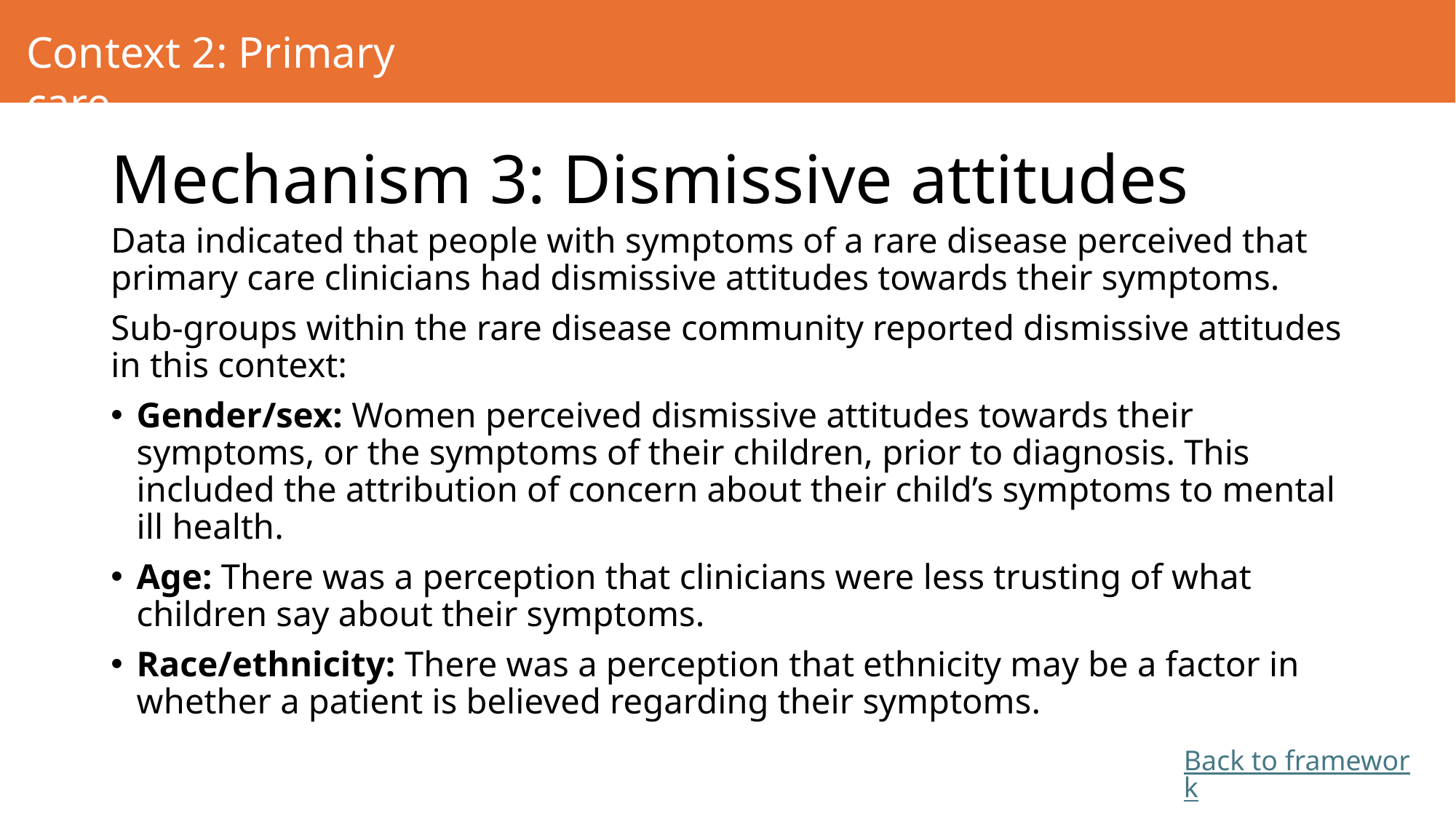

Context 2: Primary care
# Mechanism 3: Dismissive attitudes
Data indicated that people with symptoms of a rare disease perceived that primary care clinicians had dismissive attitudes towards their symptoms.
Sub-groups within the rare disease community reported dismissive attitudes in this context:
Gender/sex: Women perceived dismissive attitudes towards their symptoms, or the symptoms of their children, prior to diagnosis. This included the attribution of concern about their child’s symptoms to mental ill health.
Age: There was a perception that clinicians were less trusting of what children say about their symptoms.
Race/ethnicity: There was a perception that ethnicity may be a factor in whether a patient is believed regarding their symptoms.
Back to framework

## Slide 8
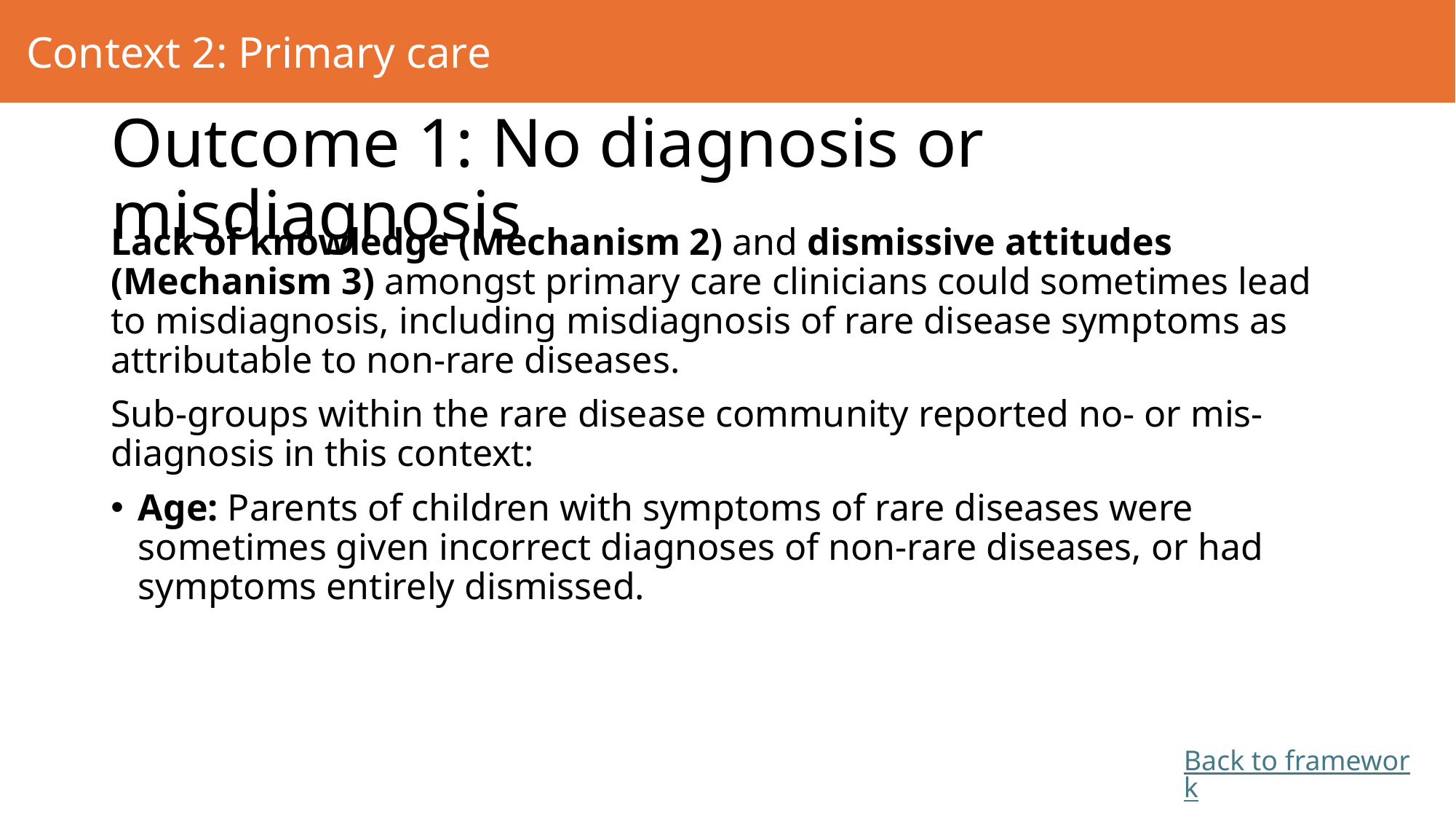

Context 2: Primary care
# Outcome 1: No diagnosis or misdiagnosis
Lack of knowledge (Mechanism 2) and dismissive attitudes (Mechanism 3) amongst primary care clinicians could sometimes lead to misdiagnosis, including misdiagnosis of rare disease symptoms as attributable to non-rare diseases.
Sub-groups within the rare disease community reported no- or mis-diagnosis in this context:
Age: Parents of children with symptoms of rare diseases were sometimes given incorrect diagnoses of non-rare diseases, or had symptoms entirely dismissed.
Back to framework

## Slide 9
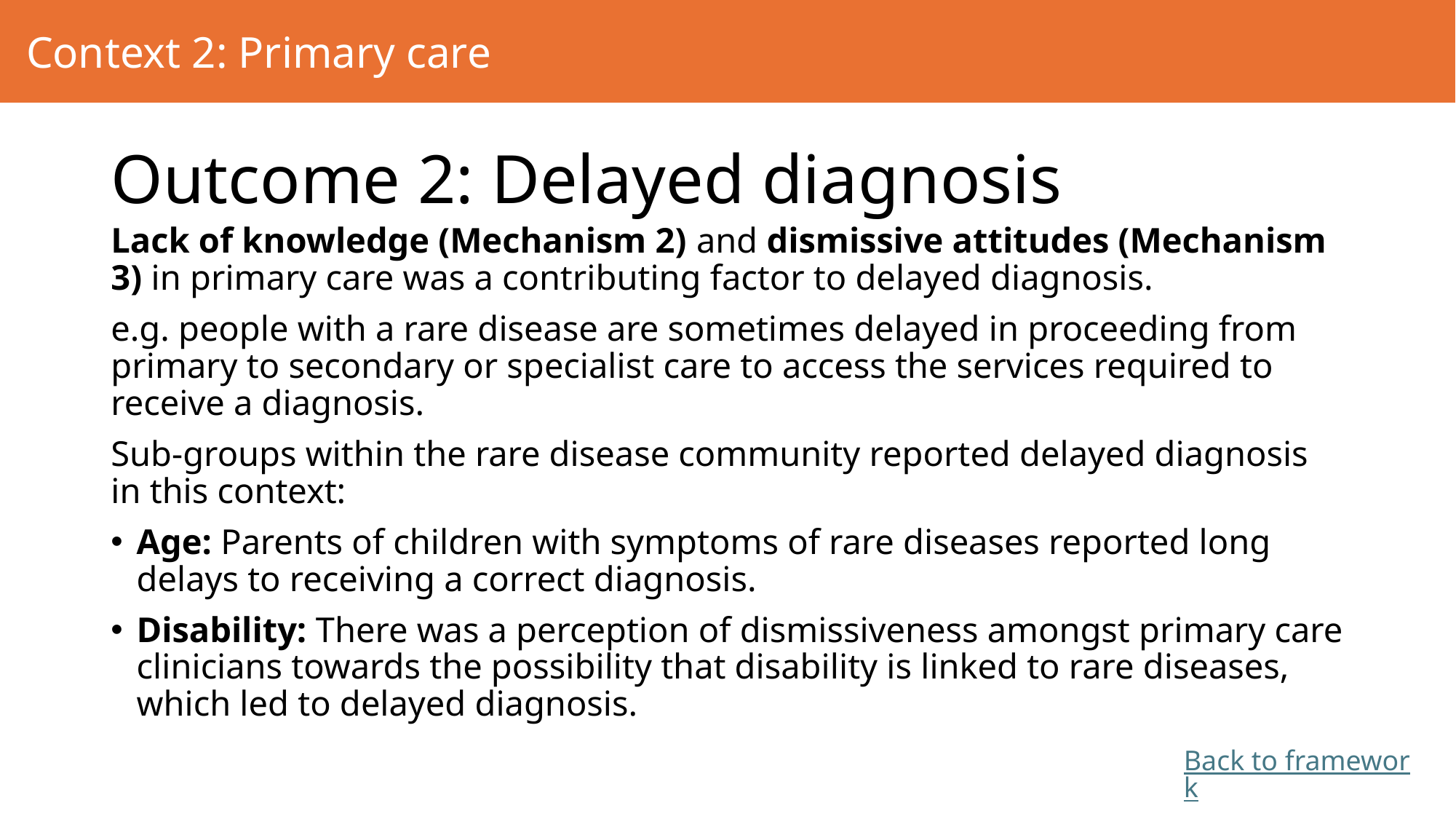

Context 2: Primary care
# Outcome 2: Delayed diagnosis
Lack of knowledge (Mechanism 2) and dismissive attitudes (Mechanism 3) in primary care was a contributing factor to delayed diagnosis.
e.g. people with a rare disease are sometimes delayed in proceeding from primary to secondary or specialist care to access the services required to receive a diagnosis.
Sub-groups within the rare disease community reported delayed diagnosis in this context:
Age: Parents of children with symptoms of rare diseases reported long delays to receiving a correct diagnosis.
Disability: There was a perception of dismissiveness amongst primary care clinicians towards the possibility that disability is linked to rare diseases, which led to delayed diagnosis.
Back to framework

## Slide 10
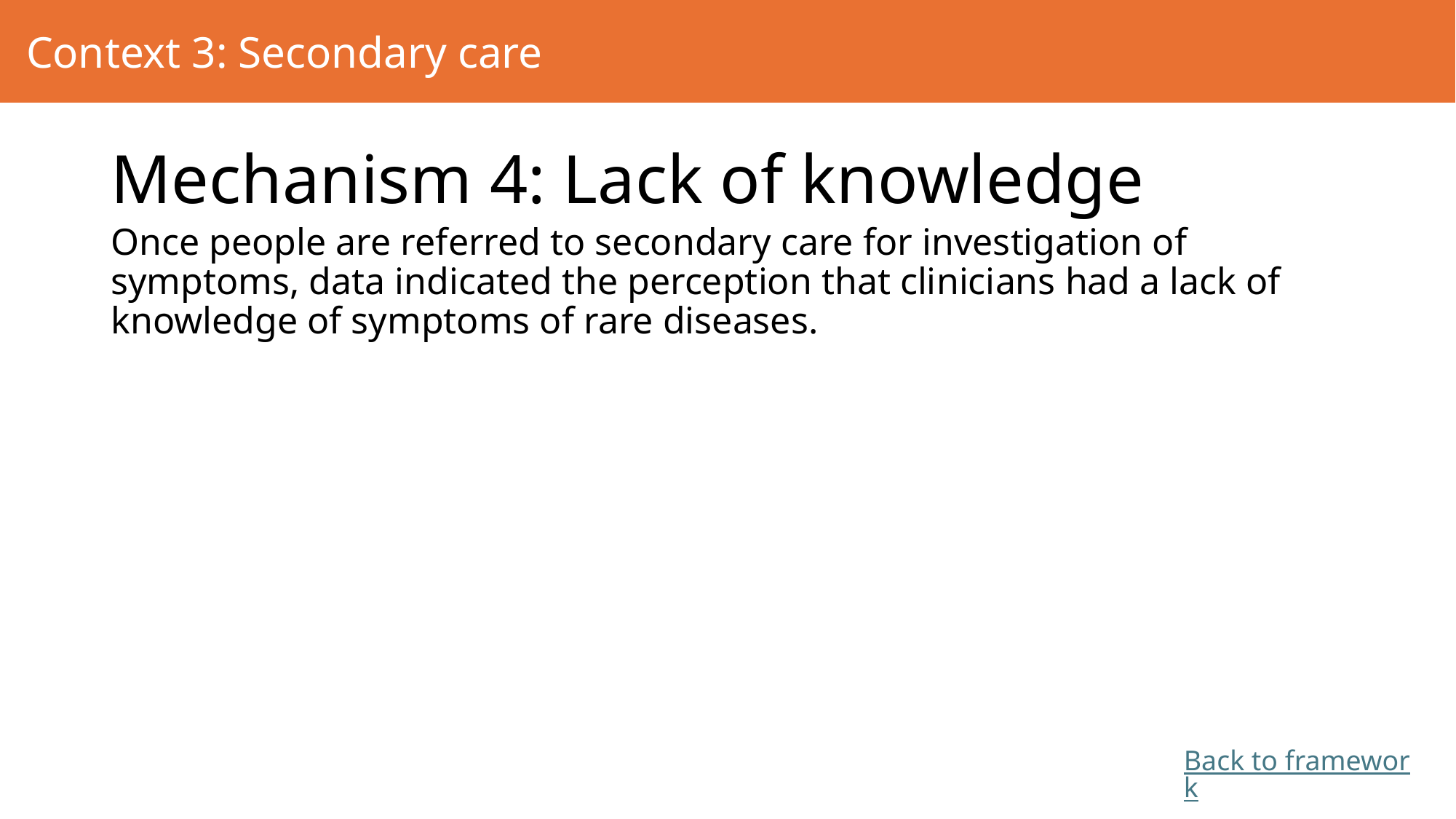

Context 3: Secondary care
# Mechanism 4: Lack of knowledge
Once people are referred to secondary care for investigation of symptoms, data indicated the perception that clinicians had a lack of knowledge of symptoms of rare diseases.
Back to framework

## Slide 11
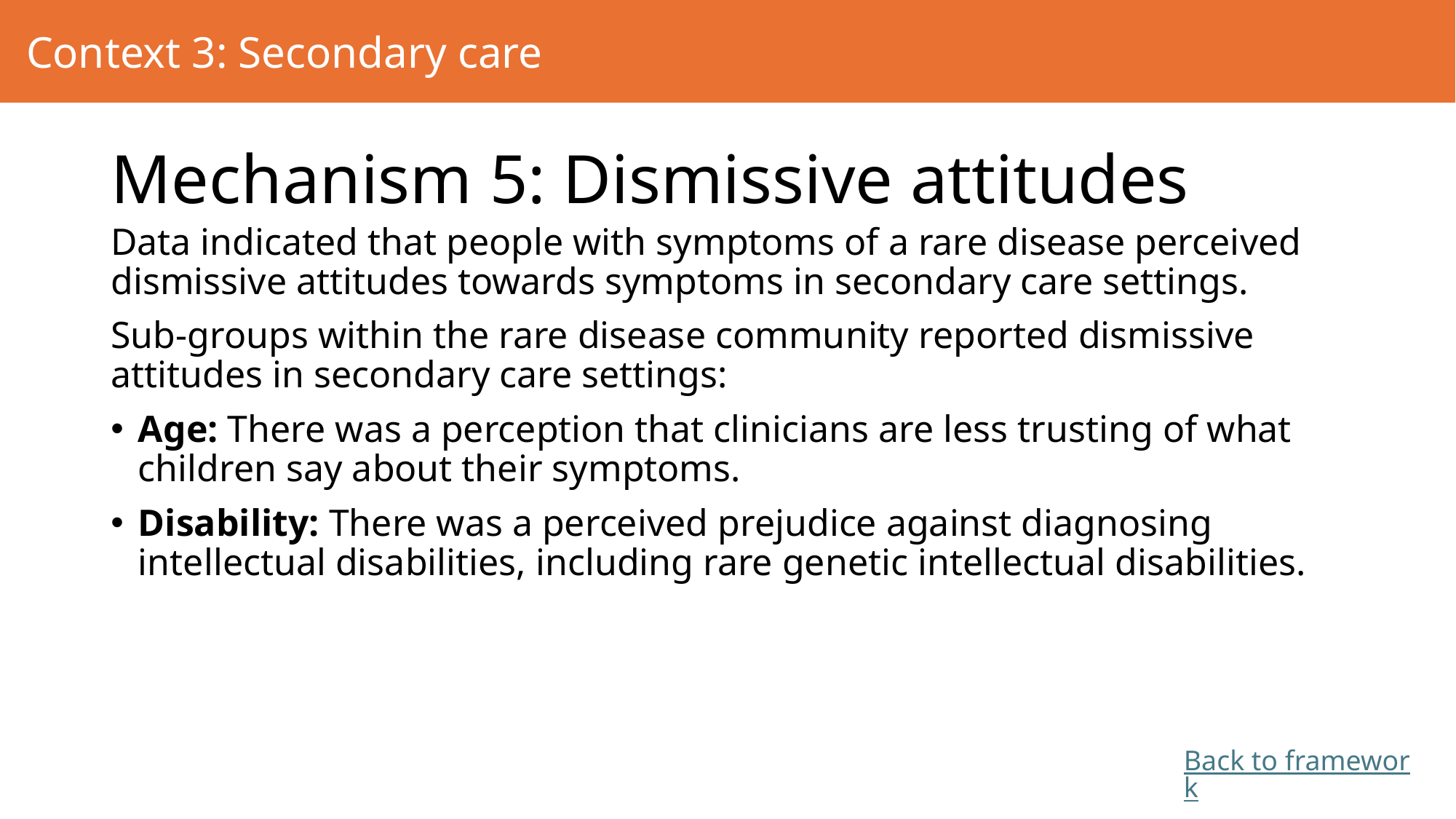

Context 3: Secondary care
# Mechanism 5: Dismissive attitudes
Data indicated that people with symptoms of a rare disease perceived dismissive attitudes towards symptoms in secondary care settings.
Sub-groups within the rare disease community reported dismissive attitudes in secondary care settings:
Age: There was a perception that clinicians are less trusting of what children say about their symptoms.
Disability: There was a perceived prejudice against diagnosing intellectual disabilities, including rare genetic intellectual disabilities.
Back to framework

## Slide 12
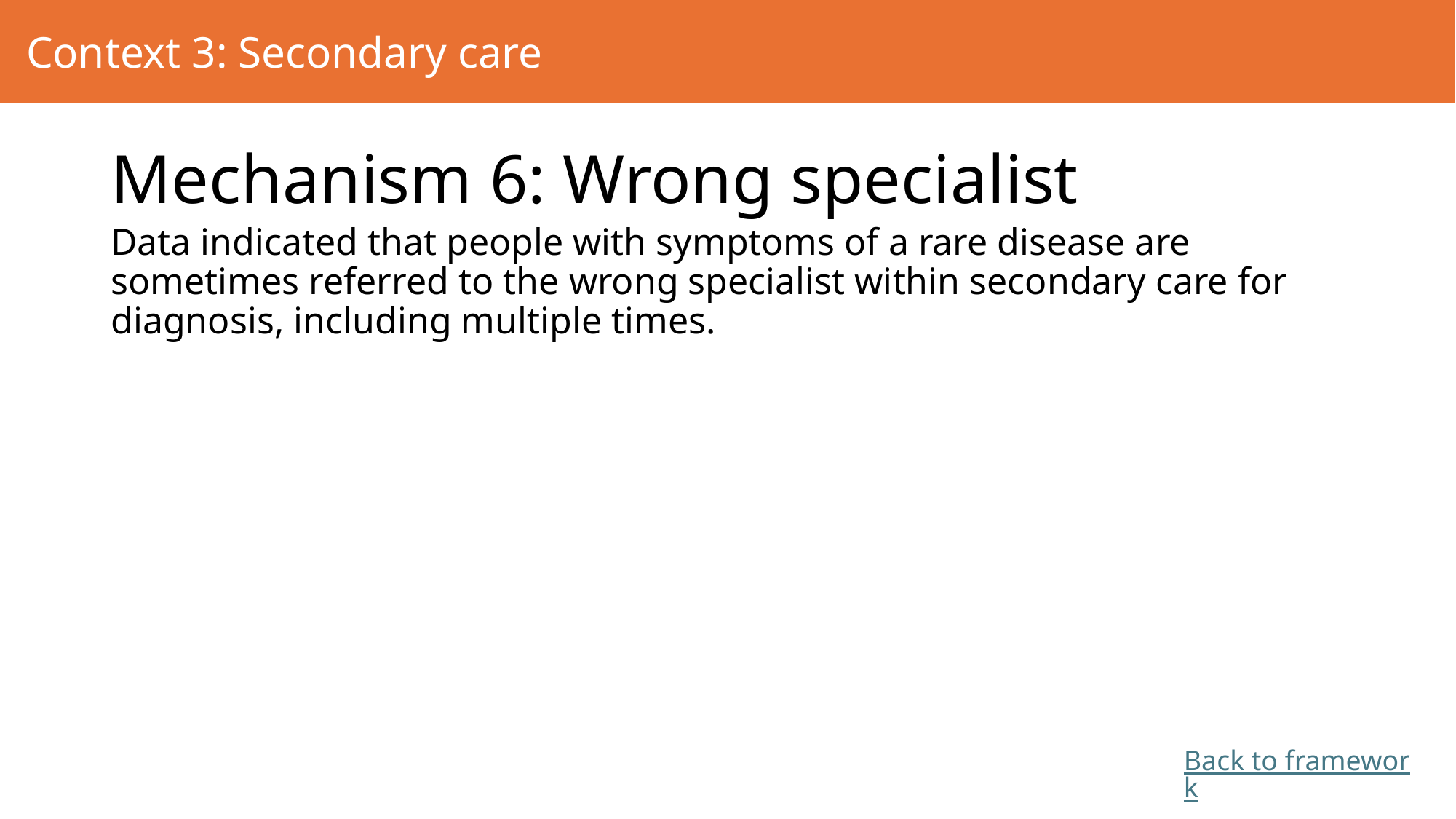

Context 3: Secondary care
# Mechanism 6: Wrong specialist
Data indicated that people with symptoms of a rare disease are sometimes referred to the wrong specialist within secondary care for diagnosis, including multiple times.
Back to framework

## Slide 13
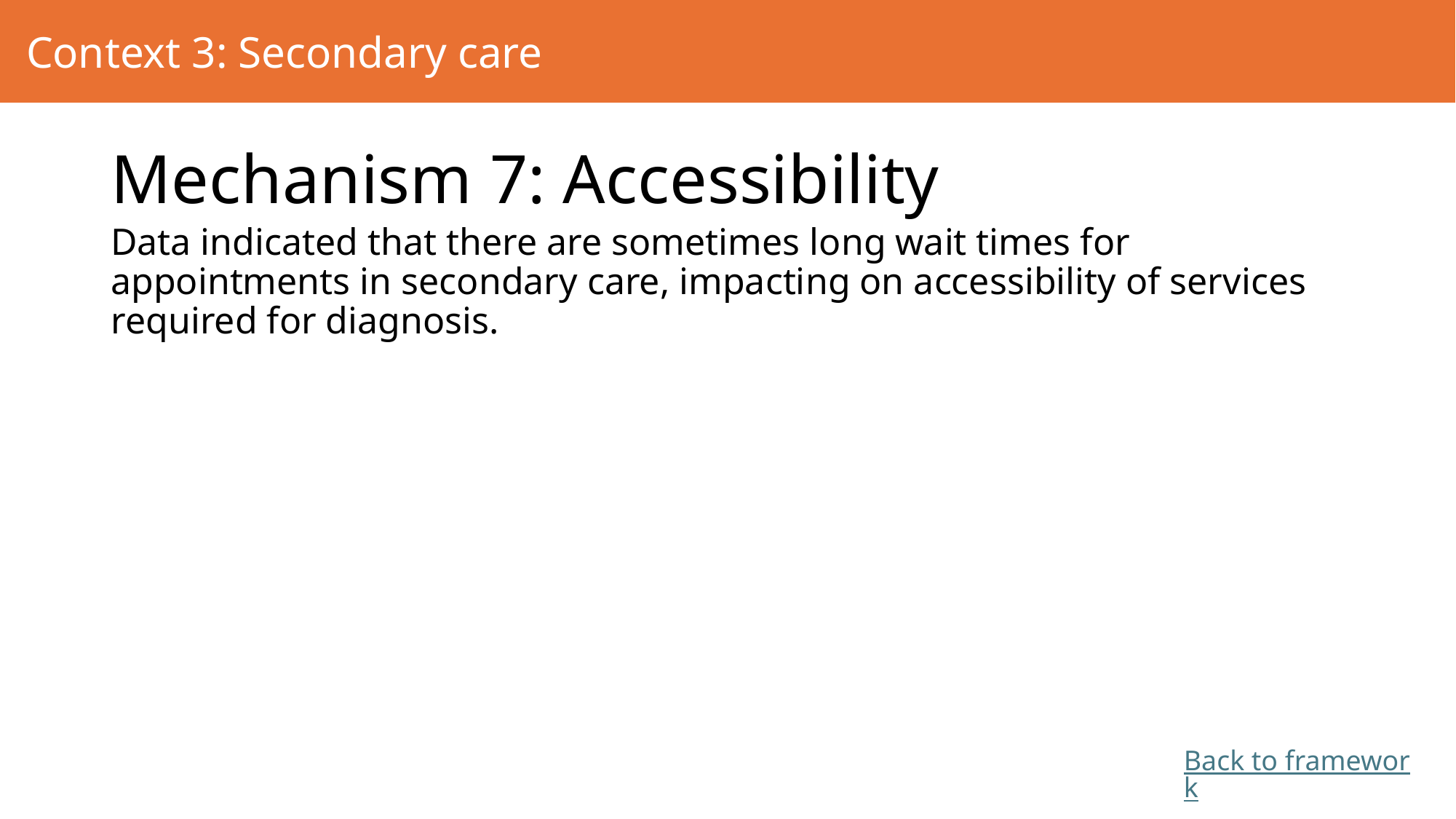

Context 3: Secondary care
# Mechanism 7: Accessibility
Data indicated that there are sometimes long wait times for appointments in secondary care, impacting on accessibility of services required for diagnosis.
Back to framework

## Slide 14
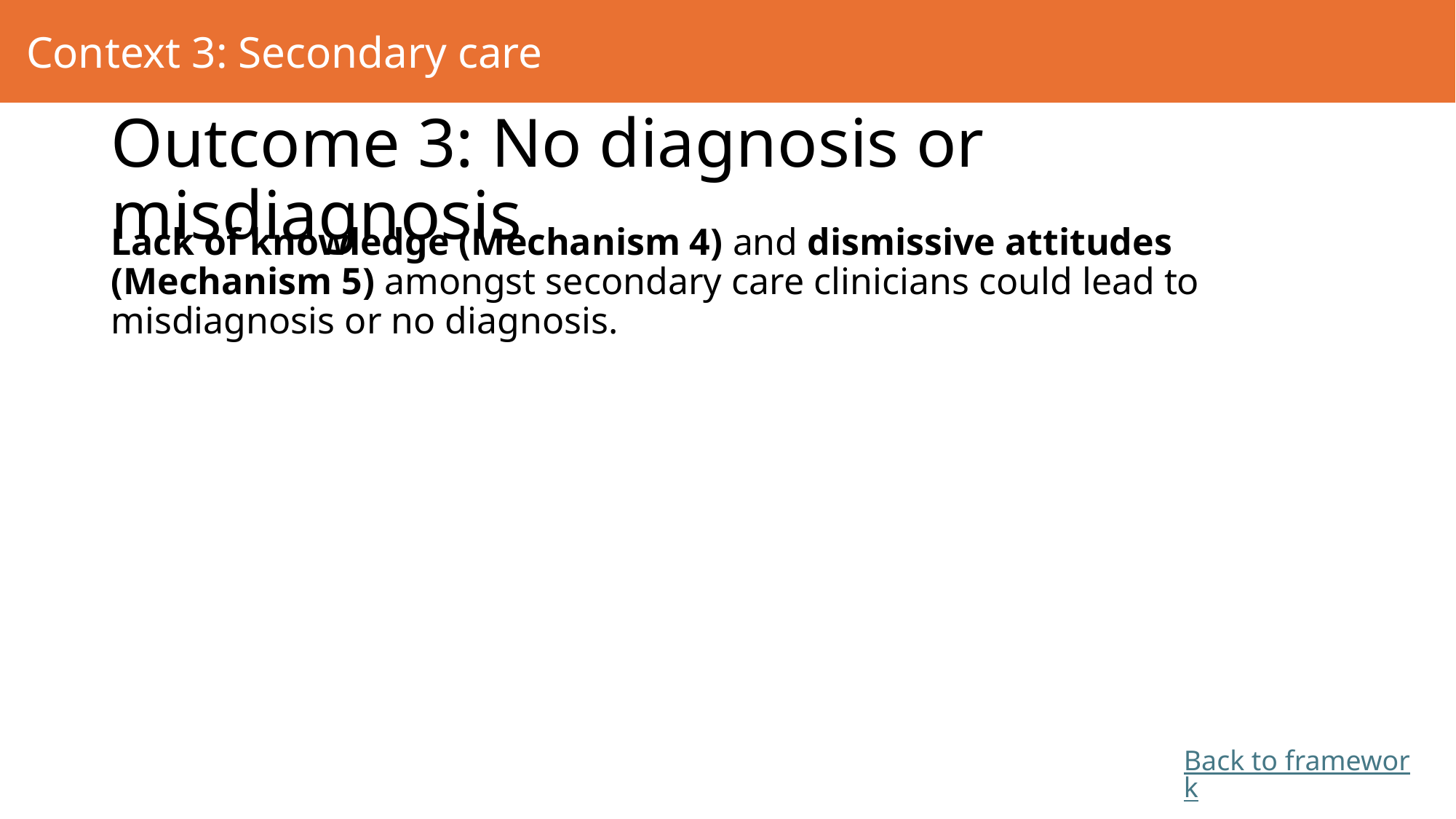

Context 3: Secondary care
# Outcome 3: No diagnosis or misdiagnosis
Lack of knowledge (Mechanism 4) and dismissive attitudes (Mechanism 5) amongst secondary care clinicians could lead to misdiagnosis or no diagnosis.
Back to framework

## Slide 15
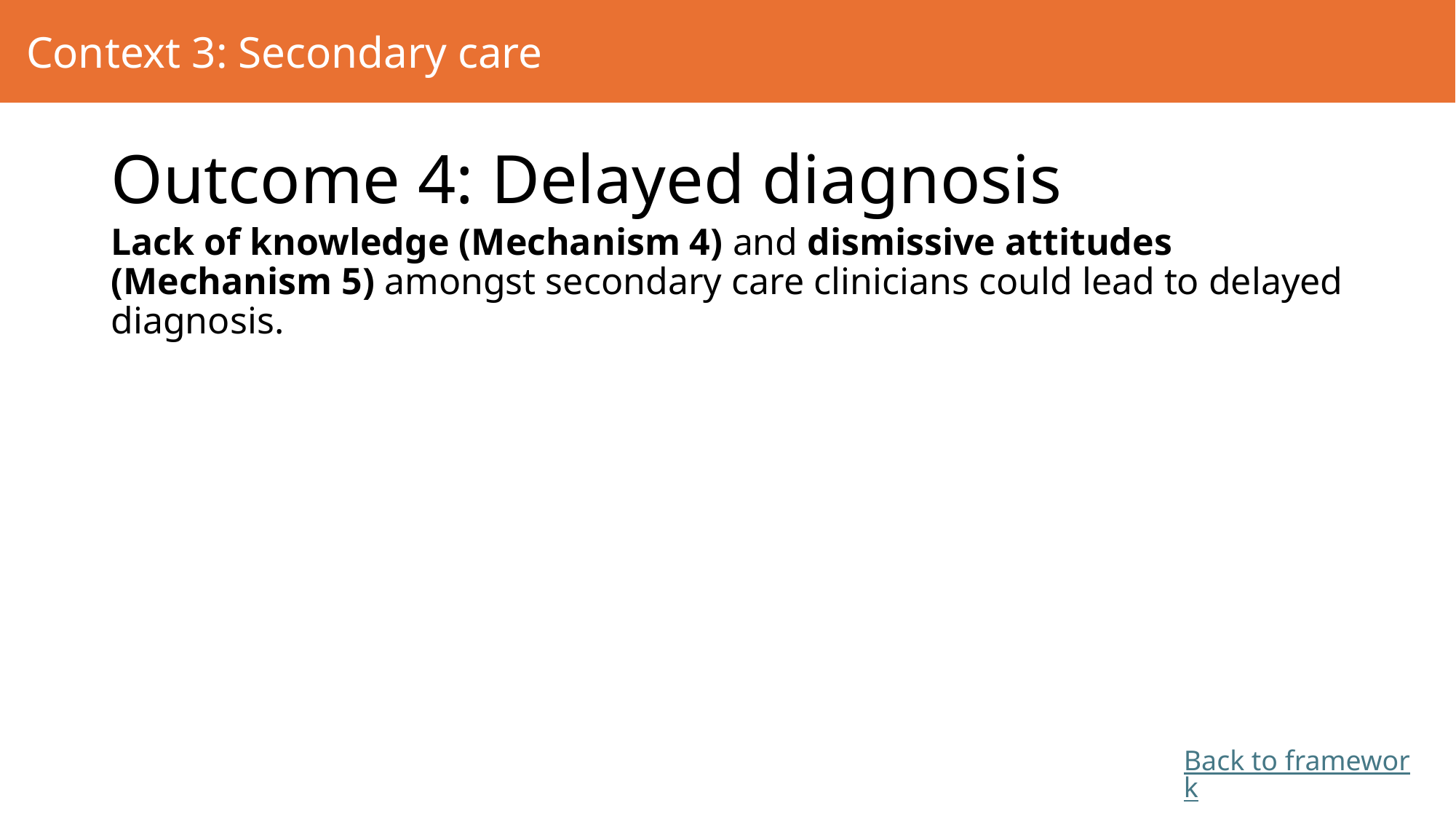

Context 3: Secondary care
# Outcome 4: Delayed diagnosis
Lack of knowledge (Mechanism 4) and dismissive attitudes (Mechanism 5) amongst secondary care clinicians could lead to delayed diagnosis.
Back to framework

## Slide 16
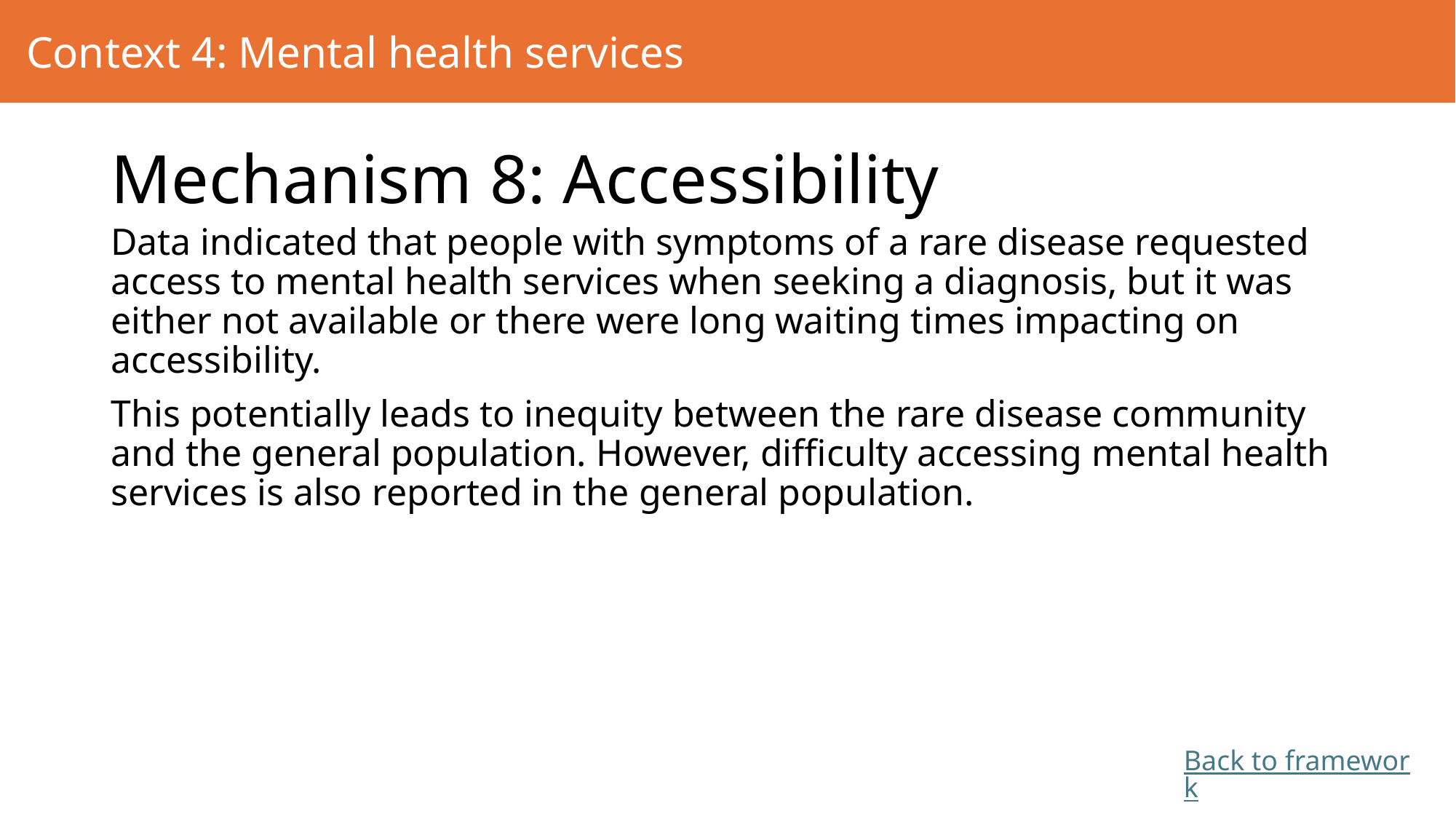

Context 4: Mental health services
# Mechanism 8: Accessibility
Data indicated that people with symptoms of a rare disease requested access to mental health services when seeking a diagnosis, but it was either not available or there were long waiting times impacting on accessibility.
This potentially leads to inequity between the rare disease community and the general population. However, difficulty accessing mental health services is also reported in the general population.
Back to framework

## Slide 17
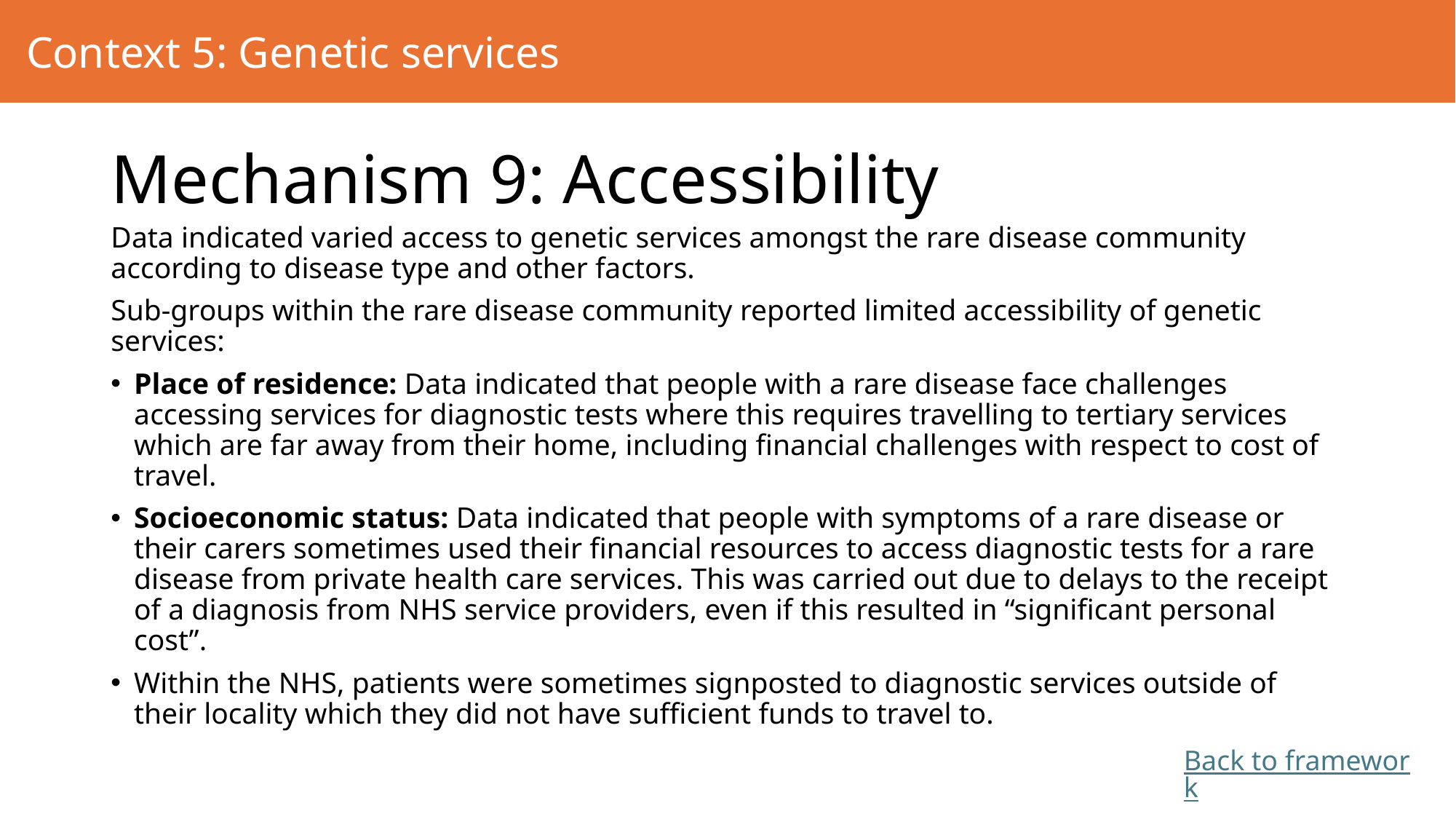

Context 5: Genetic services
# Mechanism 9: Accessibility
Data indicated varied access to genetic services amongst the rare disease community according to disease type and other factors.
Sub-groups within the rare disease community reported limited accessibility of genetic services:
Place of residence: Data indicated that people with a rare disease face challenges accessing services for diagnostic tests where this requires travelling to tertiary services which are far away from their home, including financial challenges with respect to cost of travel.
Socioeconomic status: Data indicated that people with symptoms of a rare disease or their carers sometimes used their financial resources to access diagnostic tests for a rare disease from private health care services. This was carried out due to delays to the receipt of a diagnosis from NHS service providers, even if this resulted in “significant personal cost”.
Within the NHS, patients were sometimes signposted to diagnostic services outside of their locality which they did not have sufficient funds to travel to.
Back to framework

## Slide 18
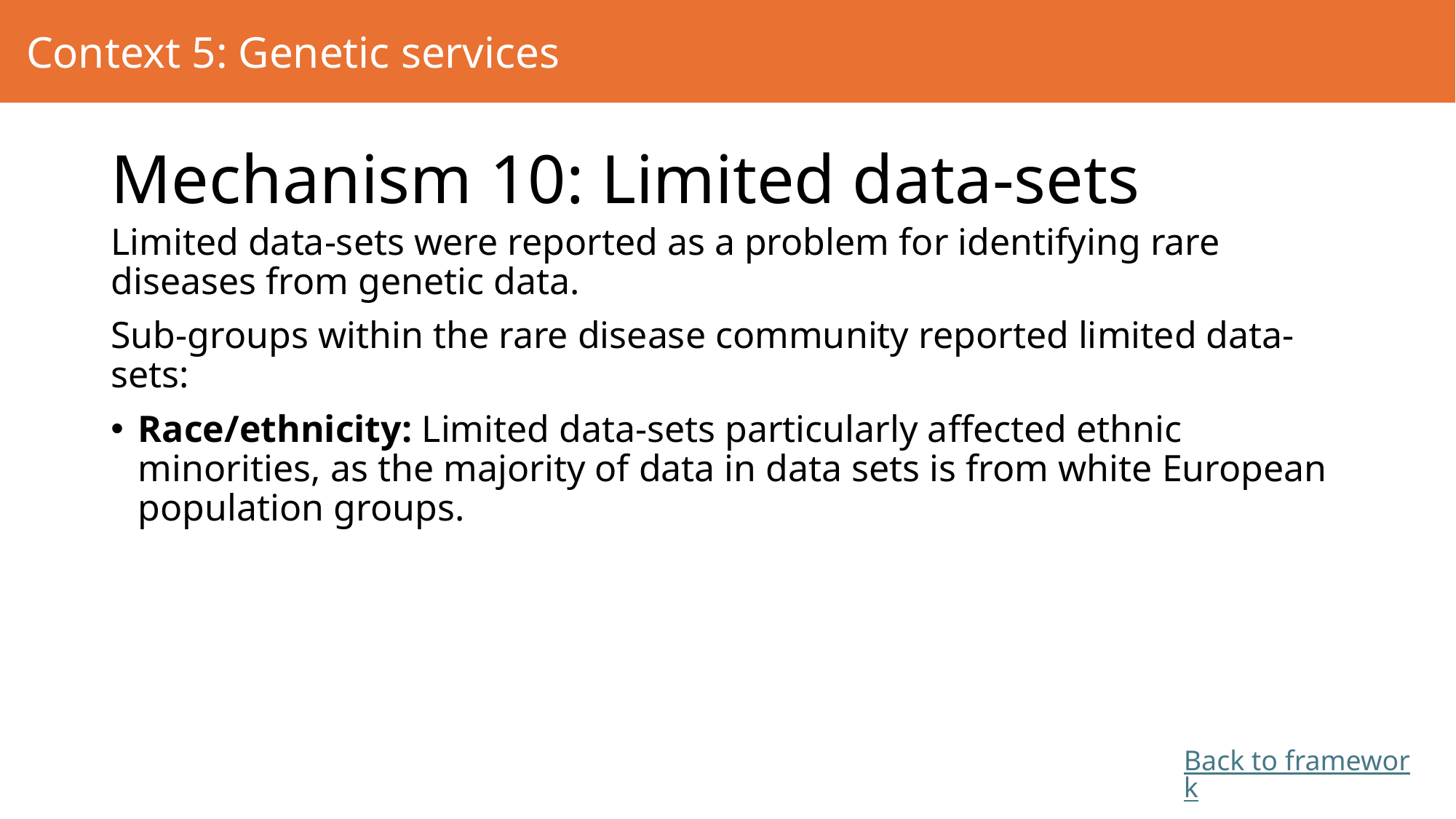

Context 5: Genetic services
# Mechanism 10: Limited data-sets
Limited data-sets were reported as a problem for identifying rare diseases from genetic data.
Sub-groups within the rare disease community reported limited data-sets:
Race/ethnicity: Limited data-sets particularly affected ethnic minorities, as the majority of data in data sets is from white European population groups.
Back to framework

## Slide 19
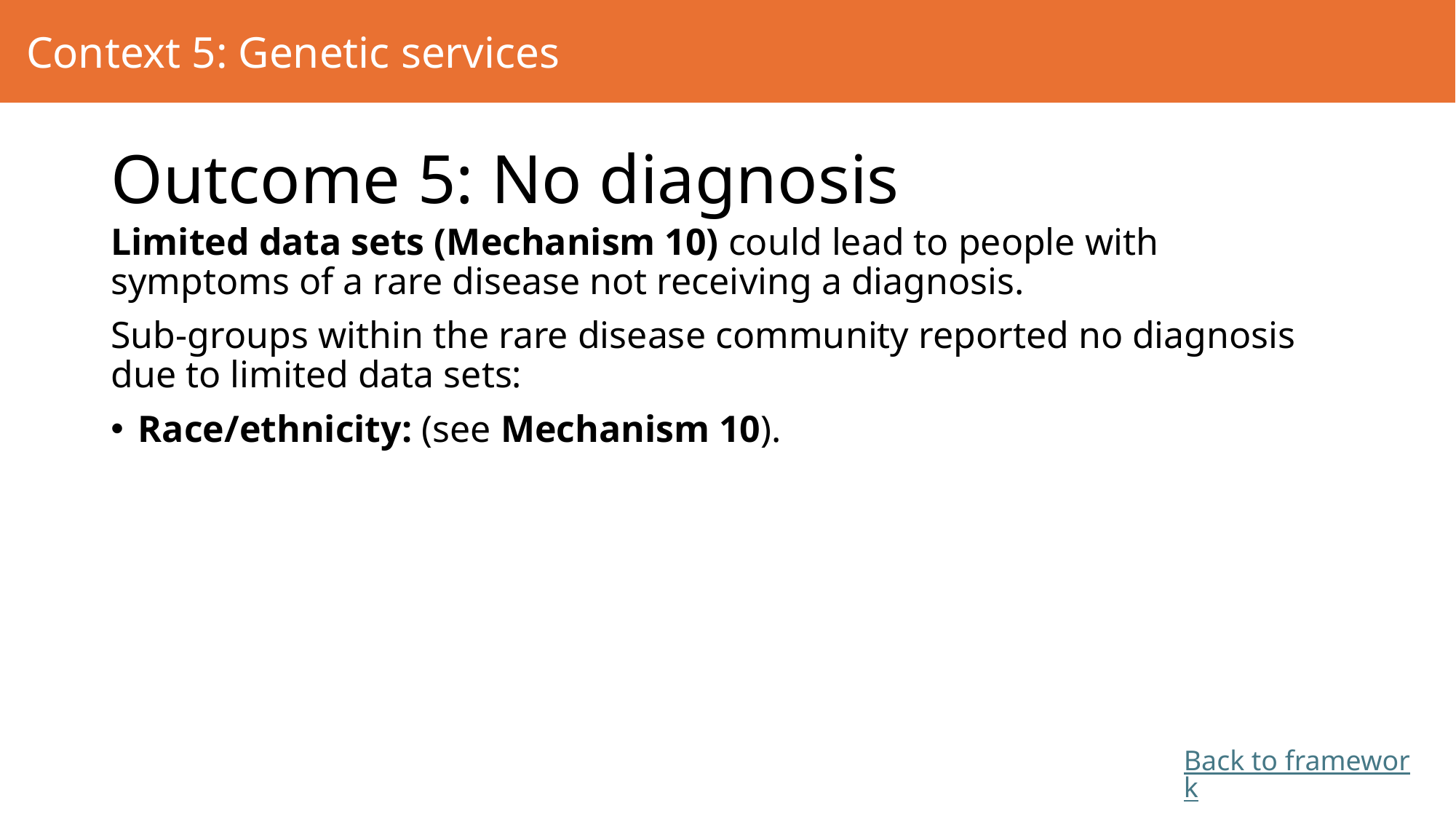

Context 5: Genetic services
# Outcome 5: No diagnosis
Limited data sets (Mechanism 10) could lead to people with symptoms of a rare disease not receiving a diagnosis.
Sub-groups within the rare disease community reported no diagnosis due to limited data sets:
Race/ethnicity: (see Mechanism 10).
Back to framework

## Slide 20
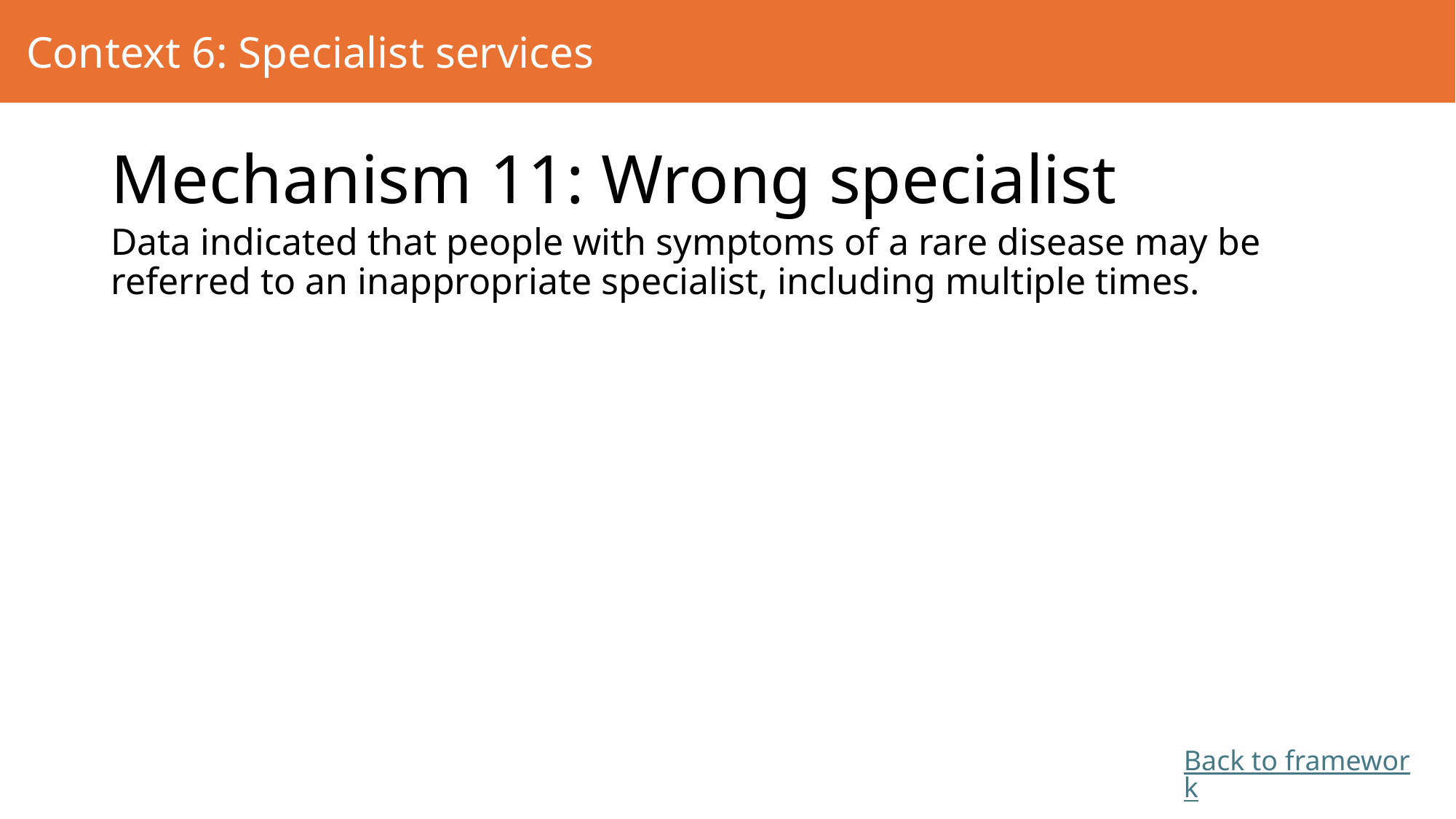

Context 6: Specialist services
# Mechanism 11: Wrong specialist
Data indicated that people with symptoms of a rare disease may be referred to an inappropriate specialist, including multiple times.
Back to framework

## Slide 21
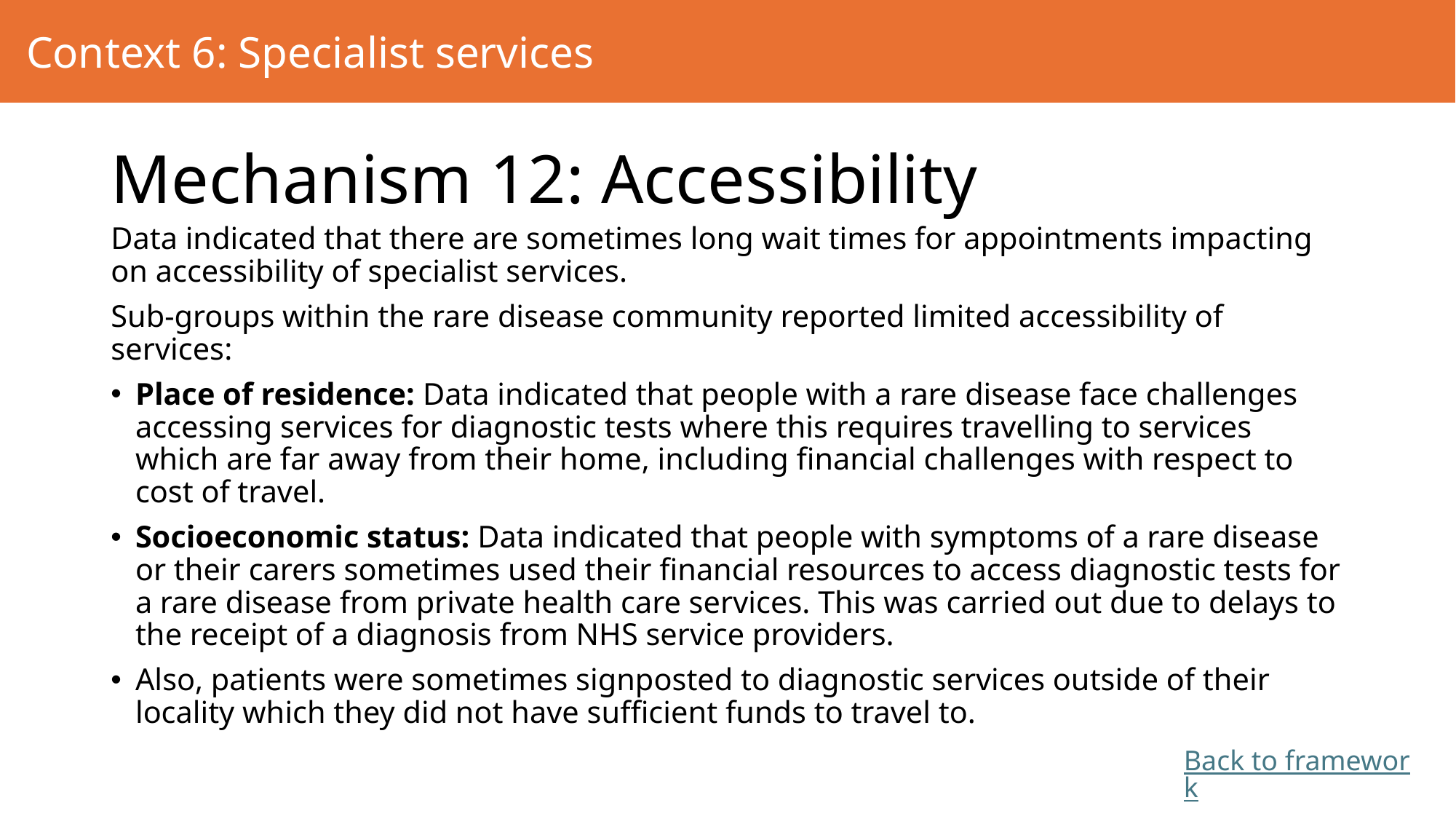

Context 6: Specialist services
# Mechanism 12: Accessibility
Data indicated that there are sometimes long wait times for appointments impacting on accessibility of specialist services.
Sub-groups within the rare disease community reported limited accessibility of services:
Place of residence: Data indicated that people with a rare disease face challenges accessing services for diagnostic tests where this requires travelling to services which are far away from their home, including financial challenges with respect to cost of travel.
Socioeconomic status: Data indicated that people with symptoms of a rare disease or their carers sometimes used their financial resources to access diagnostic tests for a rare disease from private health care services. This was carried out due to delays to the receipt of a diagnosis from NHS service providers.
Also, patients were sometimes signposted to diagnostic services outside of their locality which they did not have sufficient funds to travel to.
Back to framework

## Slide 22
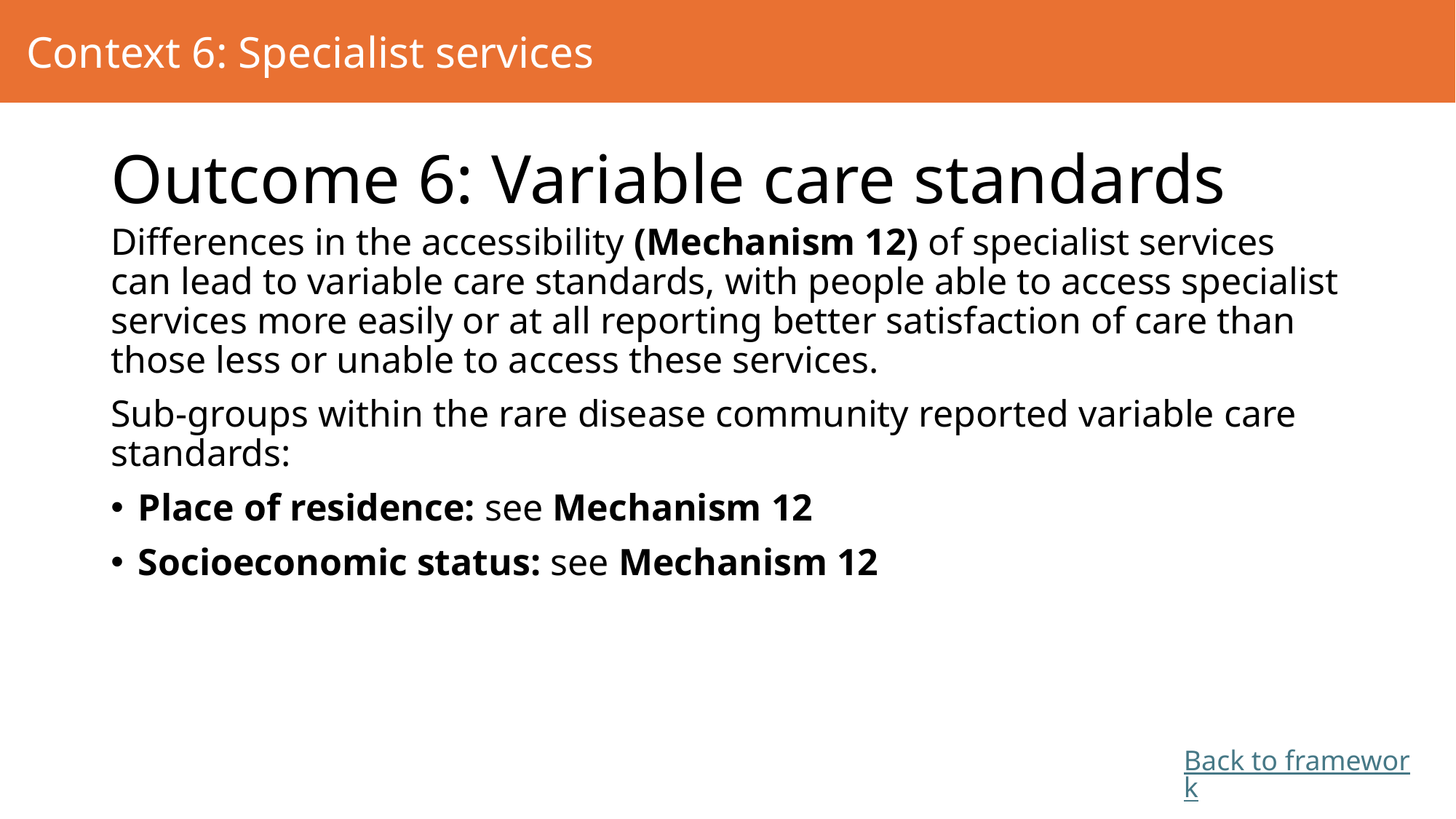

Context 6: Specialist services
# Outcome 6: Variable care standards
Differences in the accessibility (Mechanism 12) of specialist services can lead to variable care standards, with people able to access specialist services more easily or at all reporting better satisfaction of care than those less or unable to access these services.
Sub-groups within the rare disease community reported variable care standards:
Place of residence: see Mechanism 12
Socioeconomic status: see Mechanism 12
Back to framework

## Slide 23
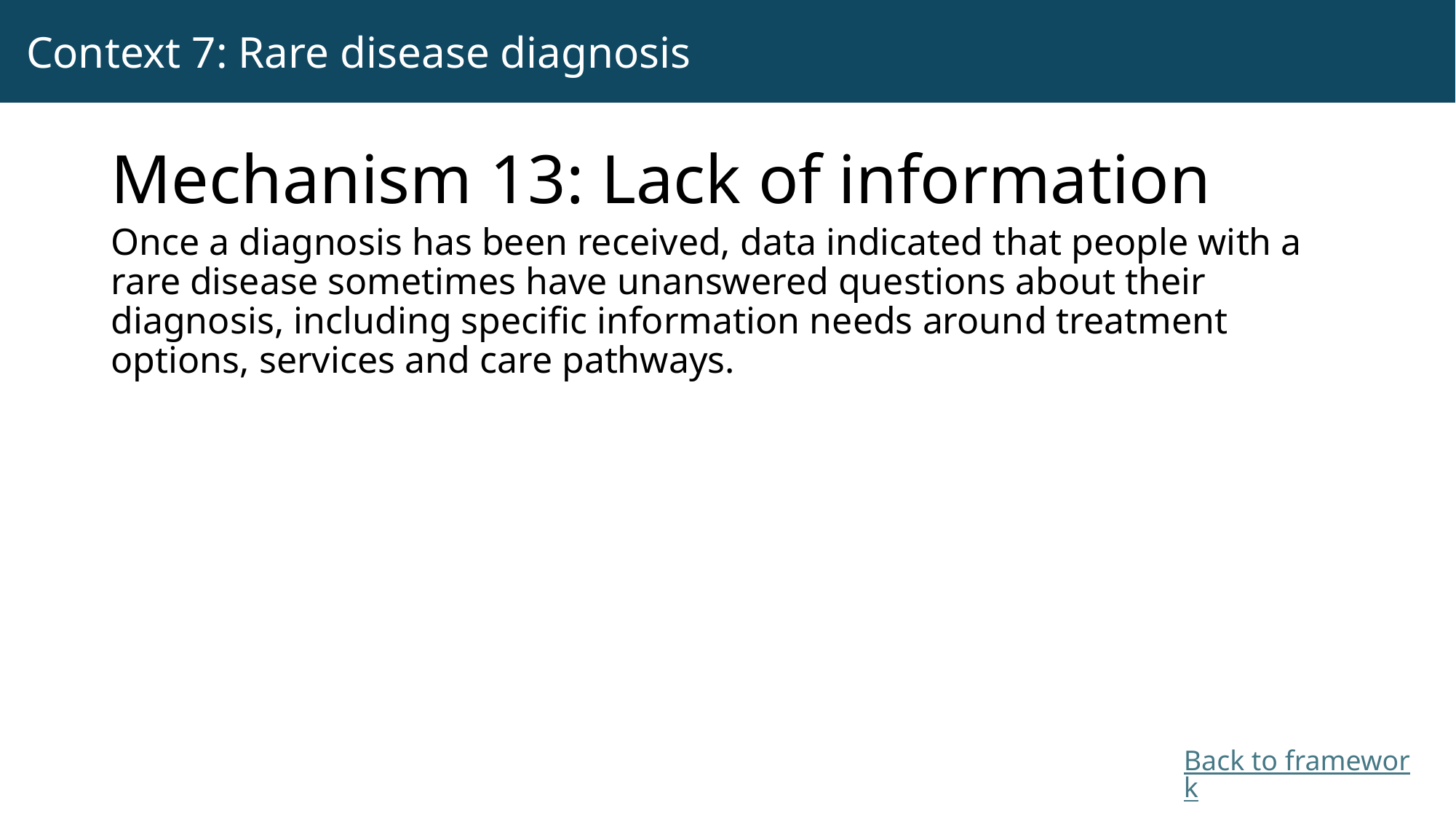

Context 7: Rare disease diagnosis
# Mechanism 13: Lack of information
Once a diagnosis has been received, data indicated that people with a rare disease sometimes have unanswered questions about their diagnosis, including specific information needs around treatment options, services and care pathways.
Back to framework

## Slide 24
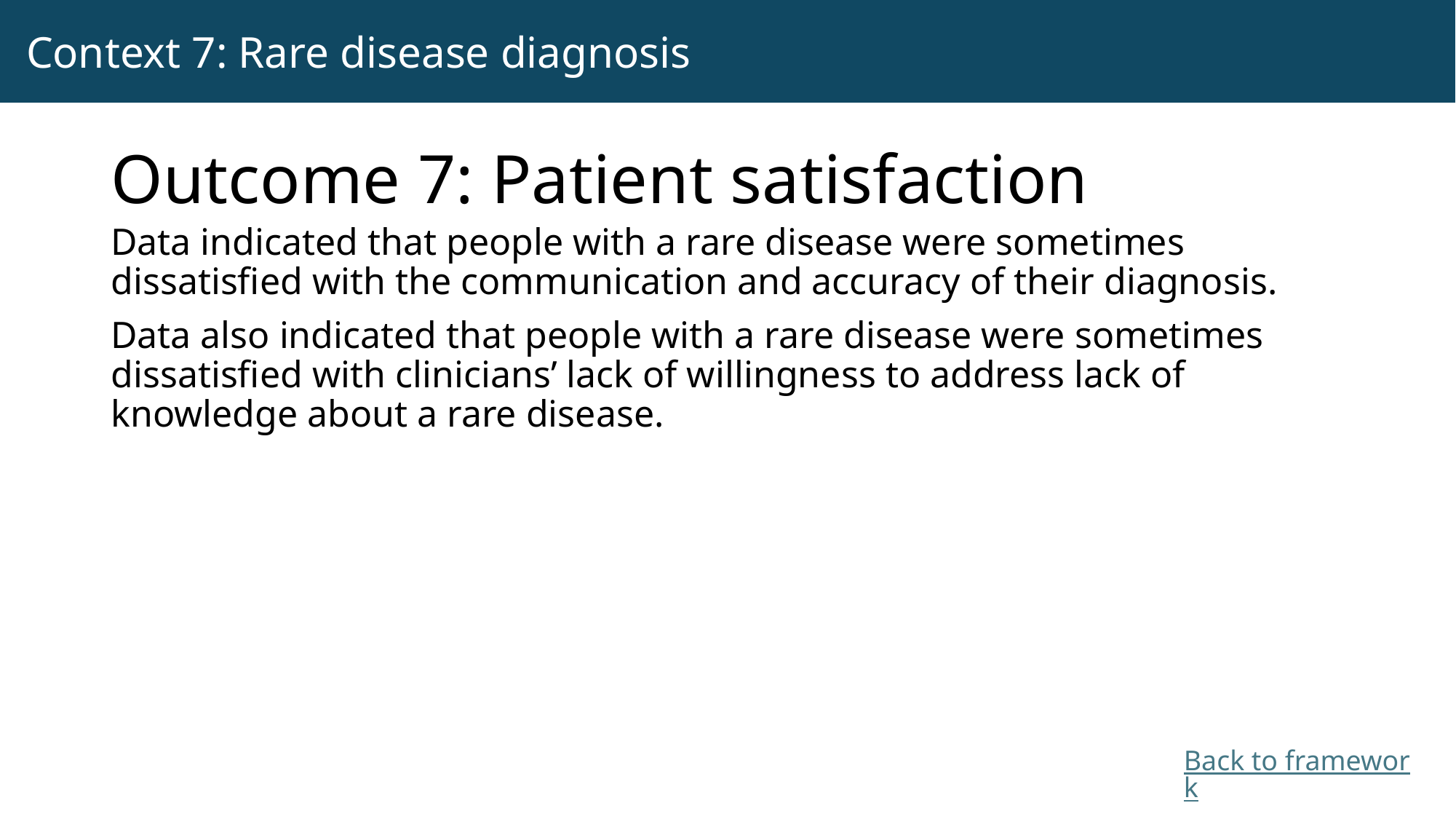

Context 7: Rare disease diagnosis
# Outcome 7: Patient satisfaction
Data indicated that people with a rare disease were sometimes dissatisfied with the communication and accuracy of their diagnosis.
Data also indicated that people with a rare disease were sometimes dissatisfied with clinicians’ lack of willingness to address lack of knowledge about a rare disease.
Back to framework

## Slide 25
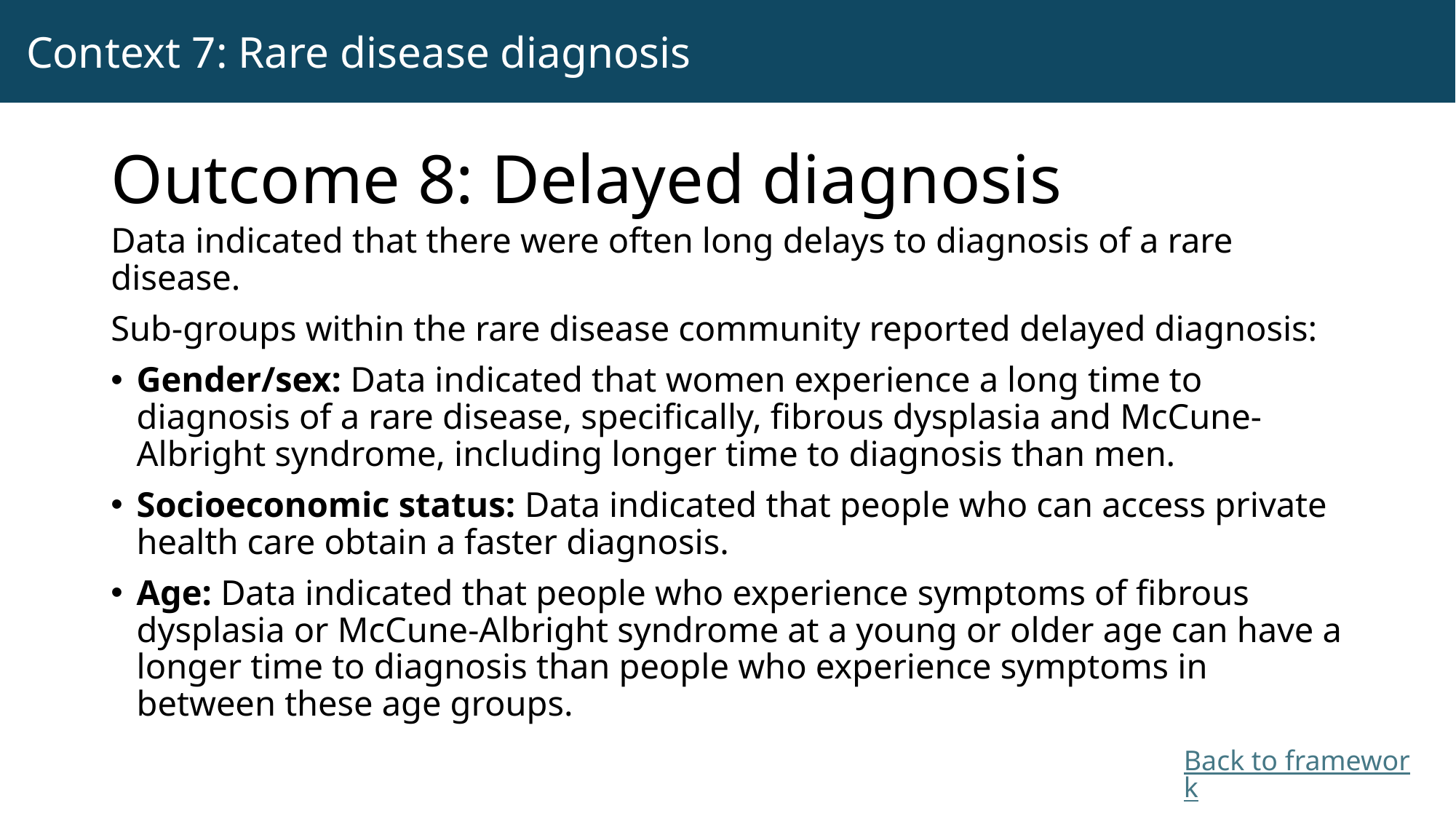

Context 7: Rare disease diagnosis
# Outcome 8: Delayed diagnosis
Data indicated that there were often long delays to diagnosis of a rare disease.
Sub-groups within the rare disease community reported delayed diagnosis:
Gender/sex: Data indicated that women experience a long time to diagnosis of a rare disease, specifically, fibrous dysplasia and McCune-Albright syndrome, including longer time to diagnosis than men.
Socioeconomic status: Data indicated that people who can access private health care obtain a faster diagnosis.
Age: Data indicated that people who experience symptoms of fibrous dysplasia or McCune-Albright syndrome at a young or older age can have a longer time to diagnosis than people who experience symptoms in between these age groups.
Back to framework

## Slide 26
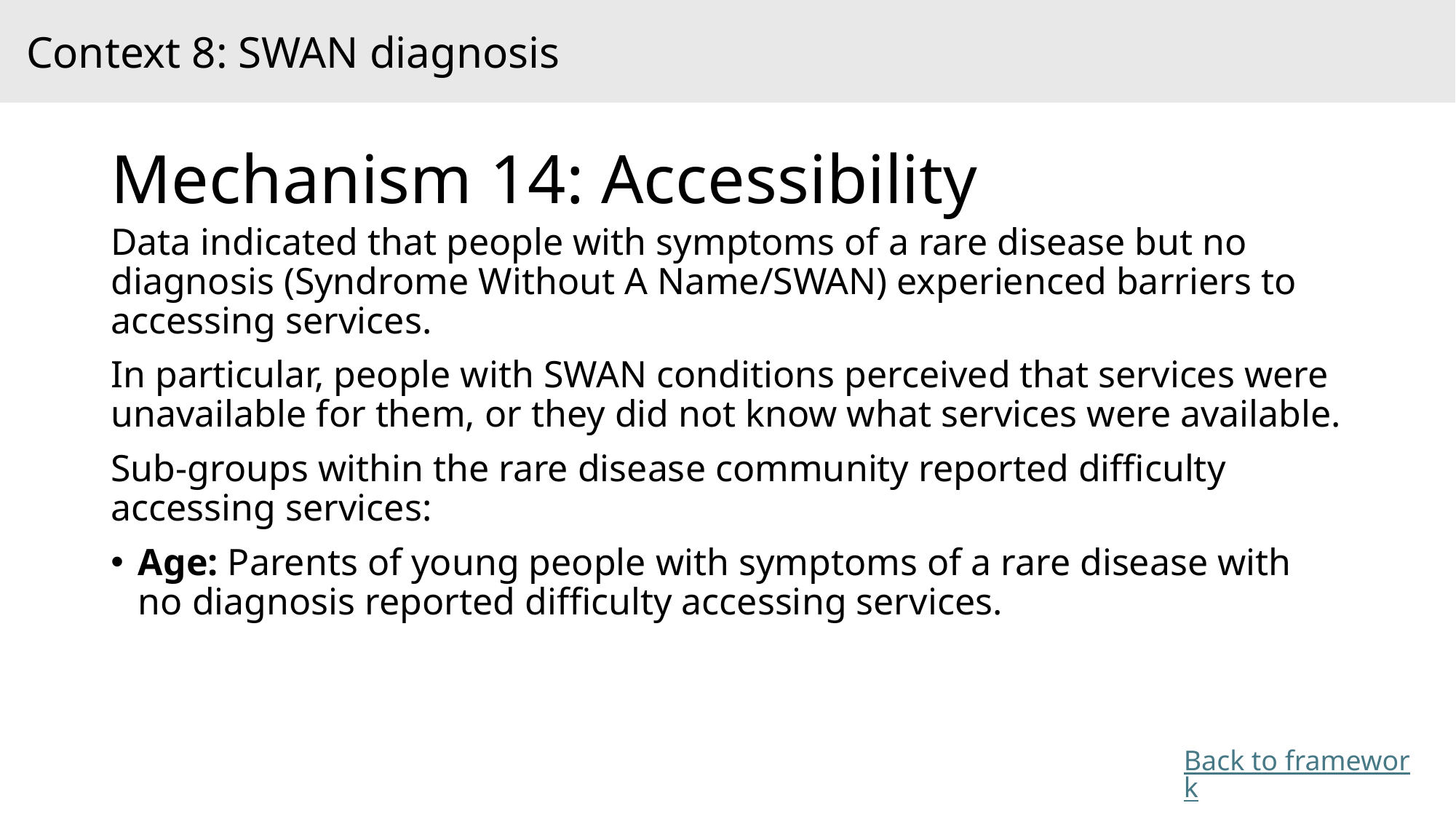

Context 8: SWAN diagnosis
# Mechanism 14: Accessibility
Data indicated that people with symptoms of a rare disease but no diagnosis (Syndrome Without A Name/SWAN) experienced barriers to accessing services.
In particular, people with SWAN conditions perceived that services were unavailable for them, or they did not know what services were available.
Sub-groups within the rare disease community reported difficulty accessing services:
Age: Parents of young people with symptoms of a rare disease with no diagnosis reported difficulty accessing services.
Back to framework

## Slide 27
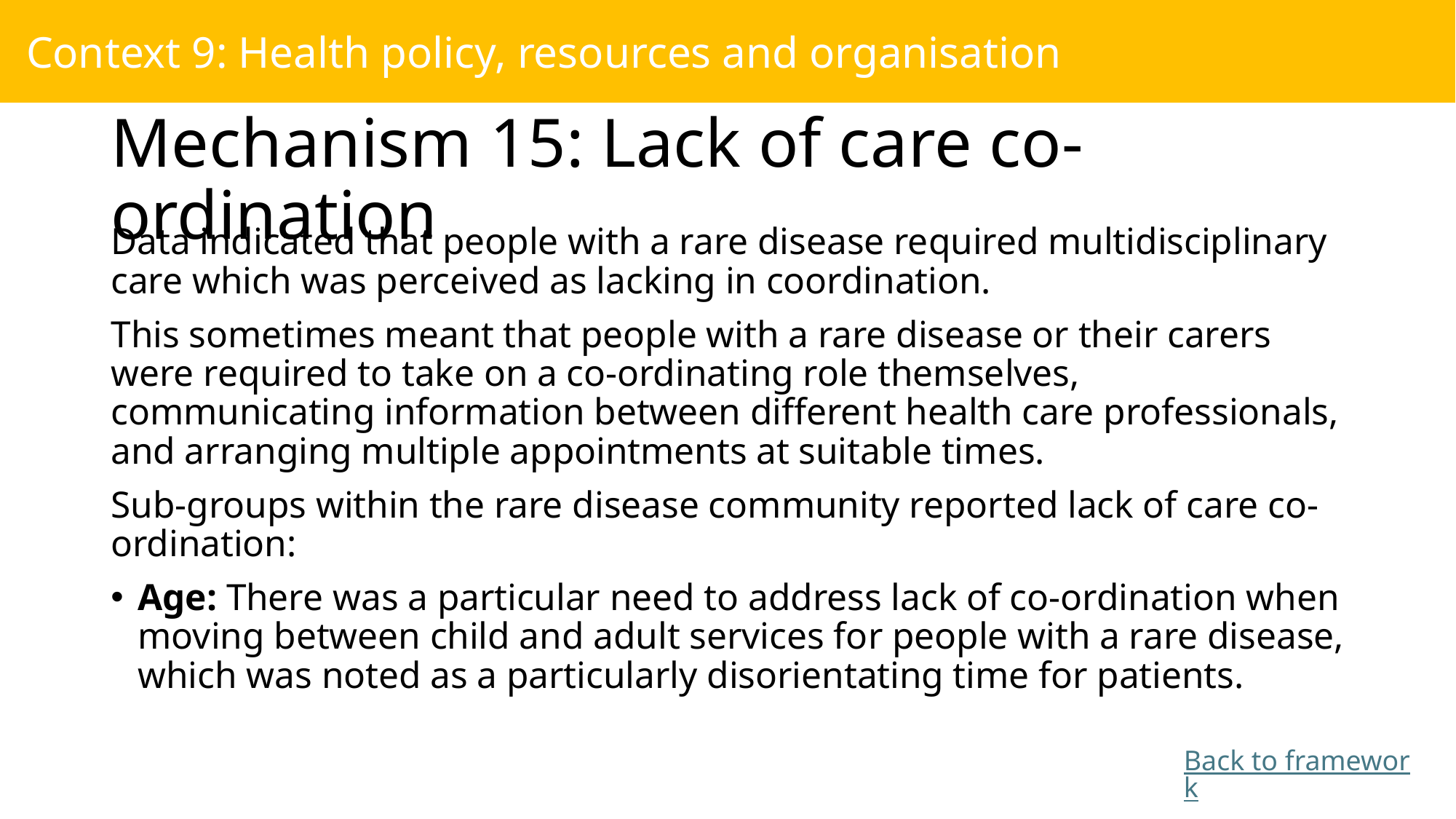

Context 9: Health policy, resources and organisation
# Mechanism 15: Lack of care co-ordination
Data indicated that people with a rare disease required multidisciplinary care which was perceived as lacking in coordination.
This sometimes meant that people with a rare disease or their carers were required to take on a co-ordinating role themselves, communicating information between different health care professionals, and arranging multiple appointments at suitable times.
Sub-groups within the rare disease community reported lack of care co-ordination:
Age: There was a particular need to address lack of co-ordination when moving between child and adult services for people with a rare disease, which was noted as a particularly disorientating time for patients.
Back to framework

## Slide 28
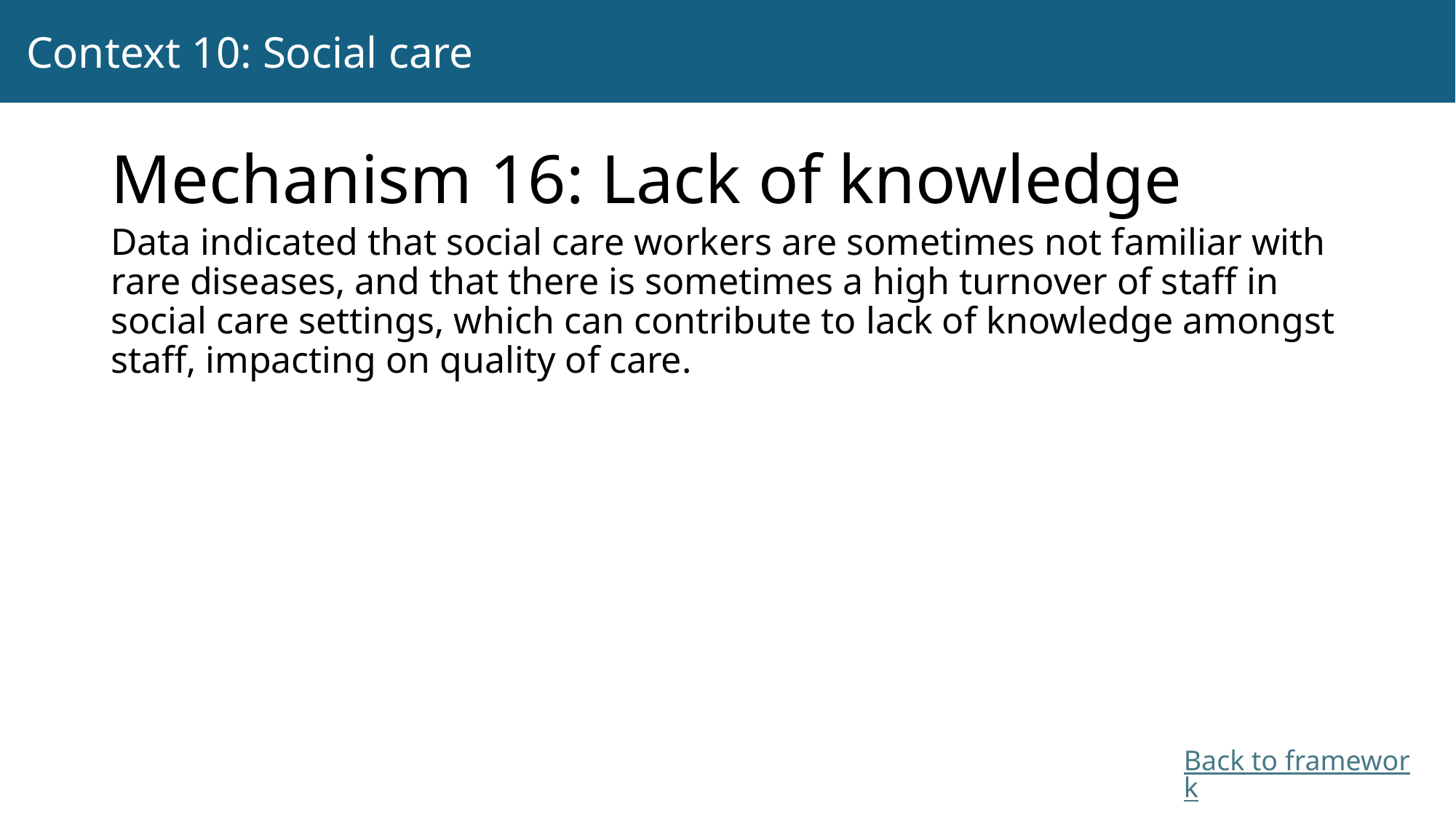

Context 10: Social care
# Mechanism 16: Lack of knowledge
Data indicated that social care workers are sometimes not familiar with rare diseases, and that there is sometimes a high turnover of staff in social care settings, which can contribute to lack of knowledge amongst staff, impacting on quality of care.
Back to framework

## Slide 29
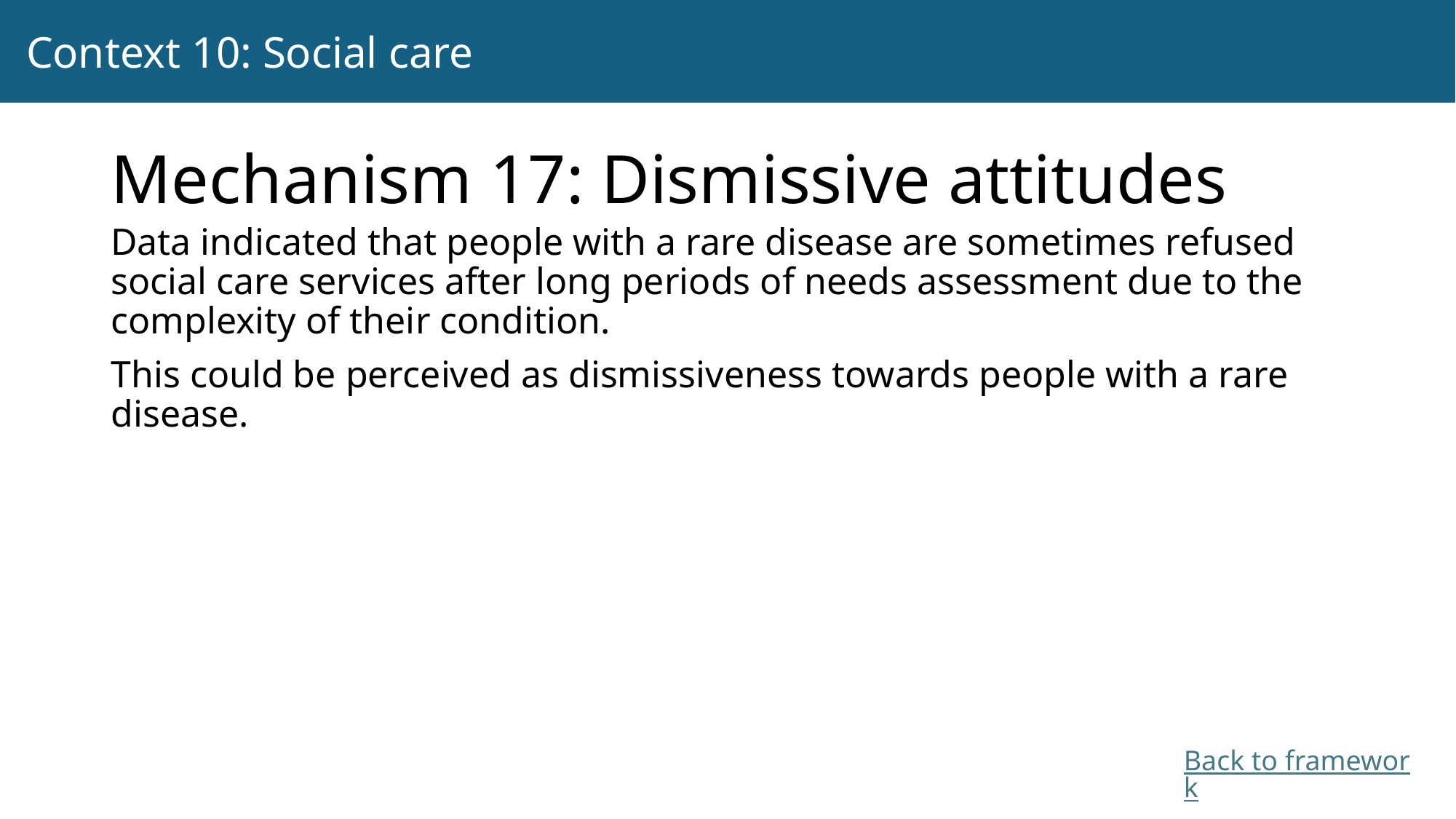

Context 10: Social care
# Mechanism 17: Dismissive attitudes
Data indicated that people with a rare disease are sometimes refused social care services after long periods of needs assessment due to the complexity of their condition.
This could be perceived as dismissiveness towards people with a rare disease.
Back to framework

## Slide 30
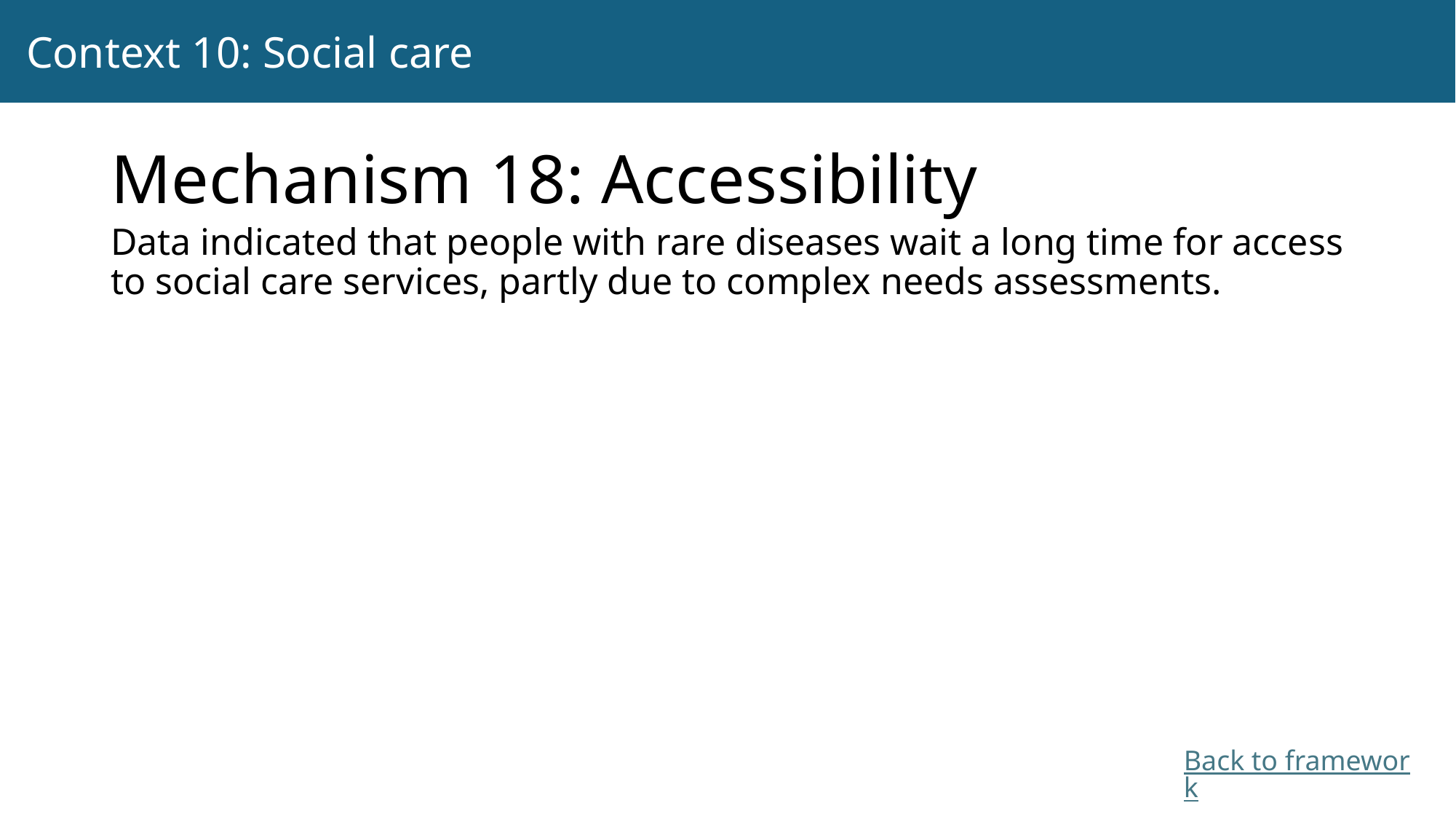

Context 10: Social care
# Mechanism 18: Accessibility
Data indicated that people with rare diseases wait a long time for access to social care services, partly due to complex needs assessments.
Back to framework

## Slide 31
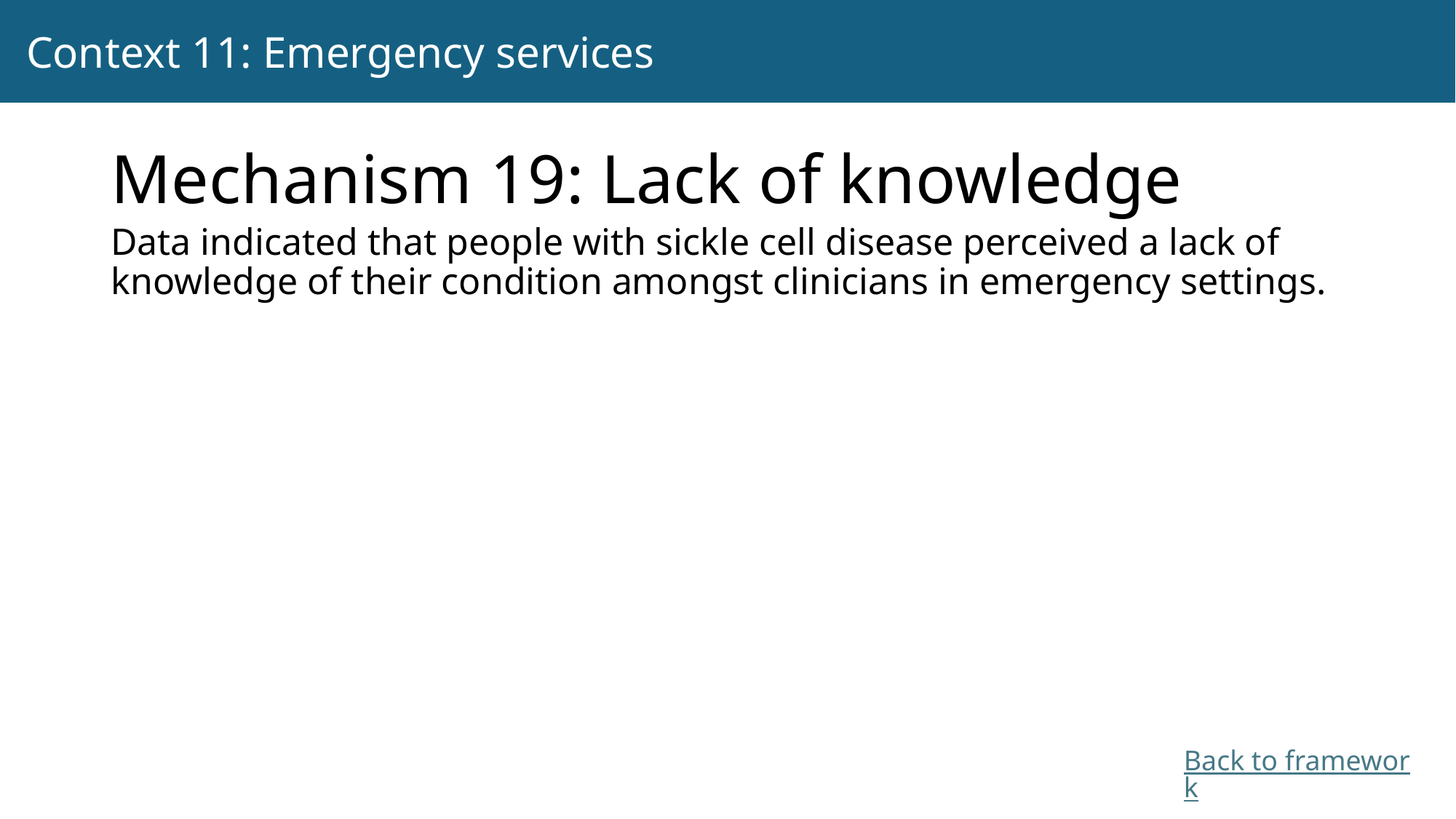

Context 11: Emergency services
# Mechanism 19: Lack of knowledge
Data indicated that people with sickle cell disease perceived a lack of knowledge of their condition amongst clinicians in emergency settings.
Back to framework

## Slide 32
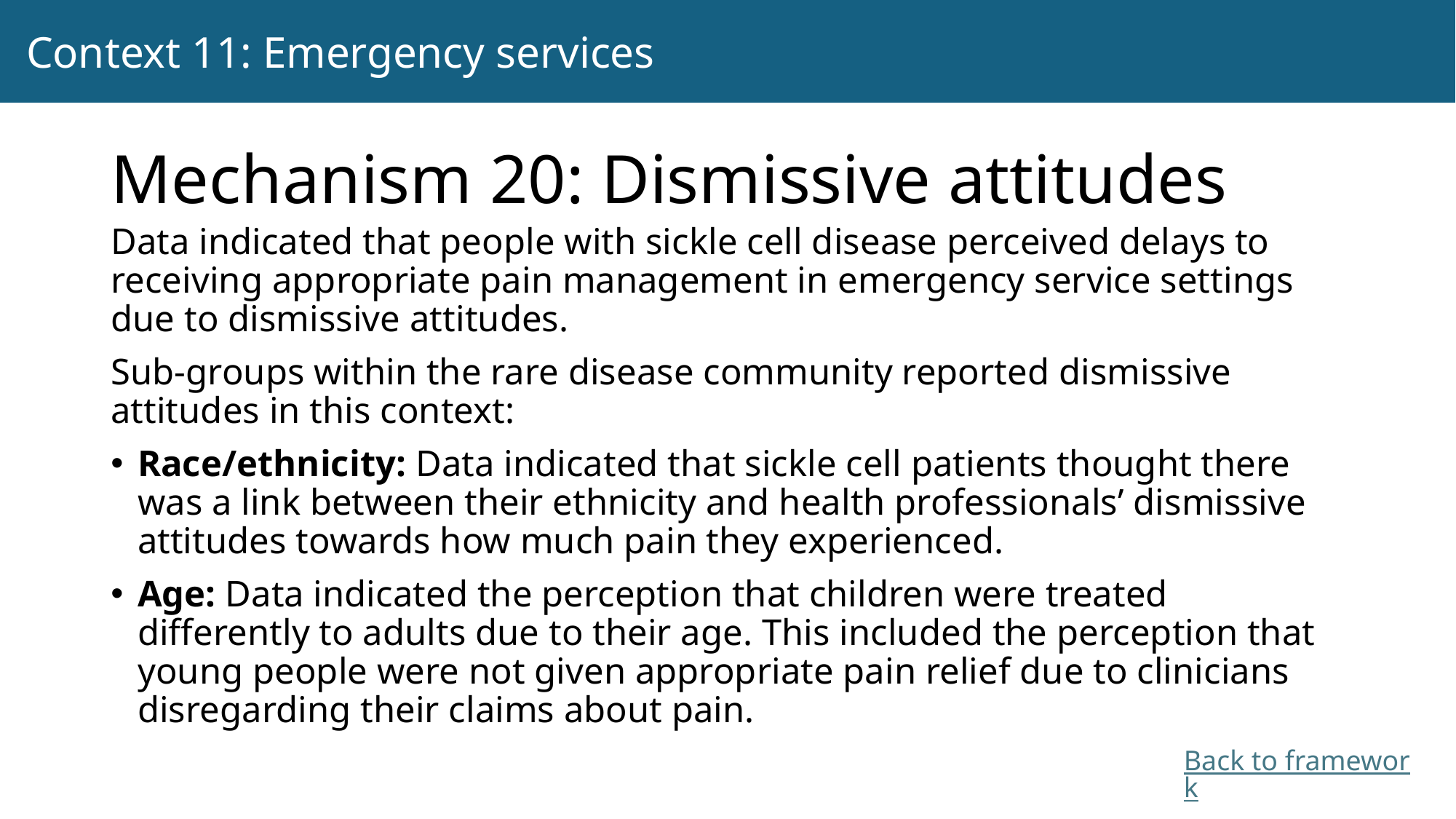

Context 11: Emergency services
# Mechanism 20: Dismissive attitudes
Data indicated that people with sickle cell disease perceived delays to receiving appropriate pain management in emergency service settings due to dismissive attitudes.
Sub-groups within the rare disease community reported dismissive attitudes in this context:
Race/ethnicity: Data indicated that sickle cell patients thought there was a link between their ethnicity and health professionals’ dismissive attitudes towards how much pain they experienced.
Age: Data indicated the perception that children were treated differently to adults due to their age. This included the perception that young people were not given appropriate pain relief due to clinicians disregarding their claims about pain.
Back to framework

## Slide 33
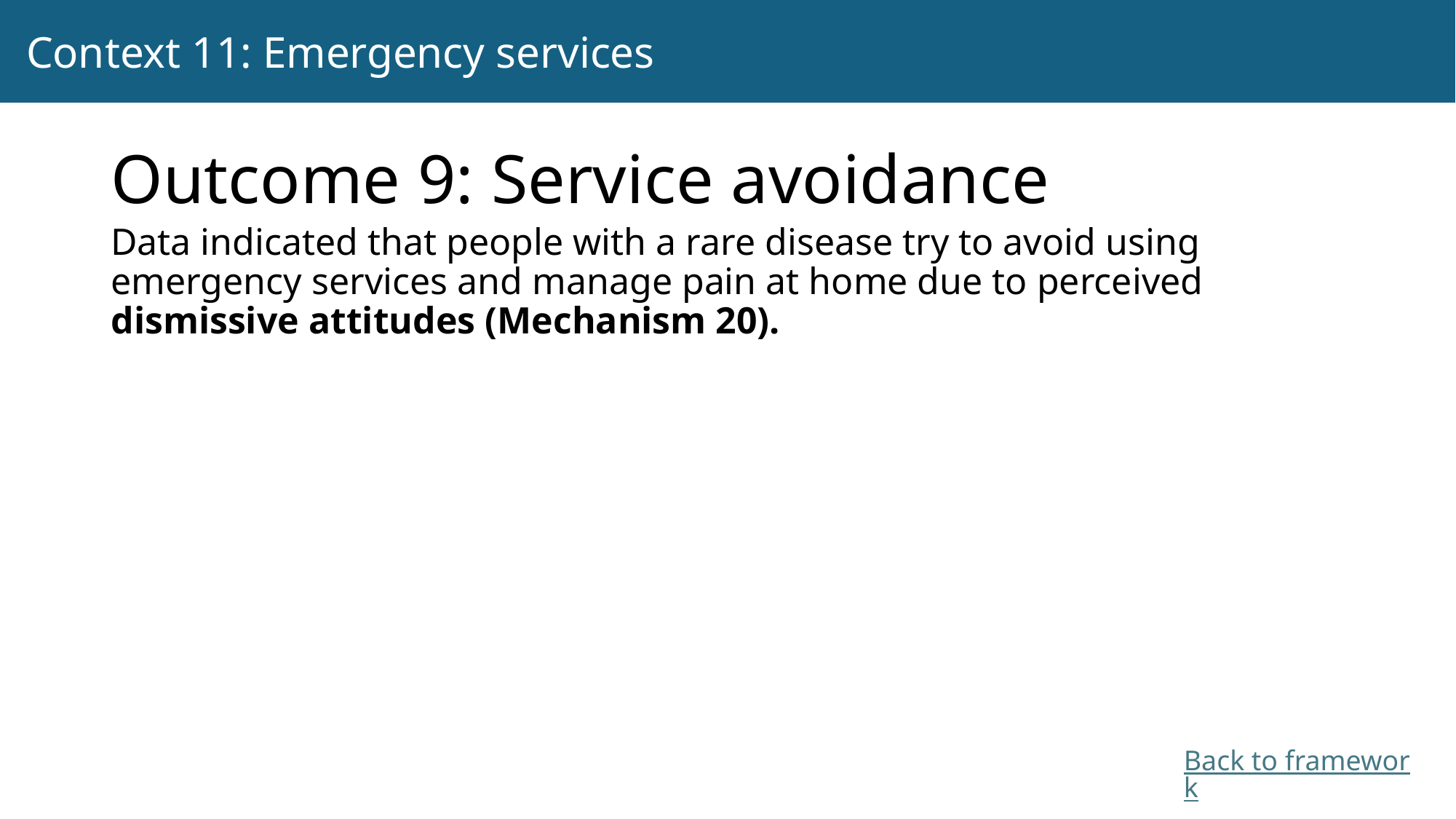

Context 11: Emergency services
# Outcome 9: Service avoidance
Data indicated that people with a rare disease try to avoid using emergency services and manage pain at home due to perceived dismissive attitudes (Mechanism 20).
Back to framework

## Slide 34
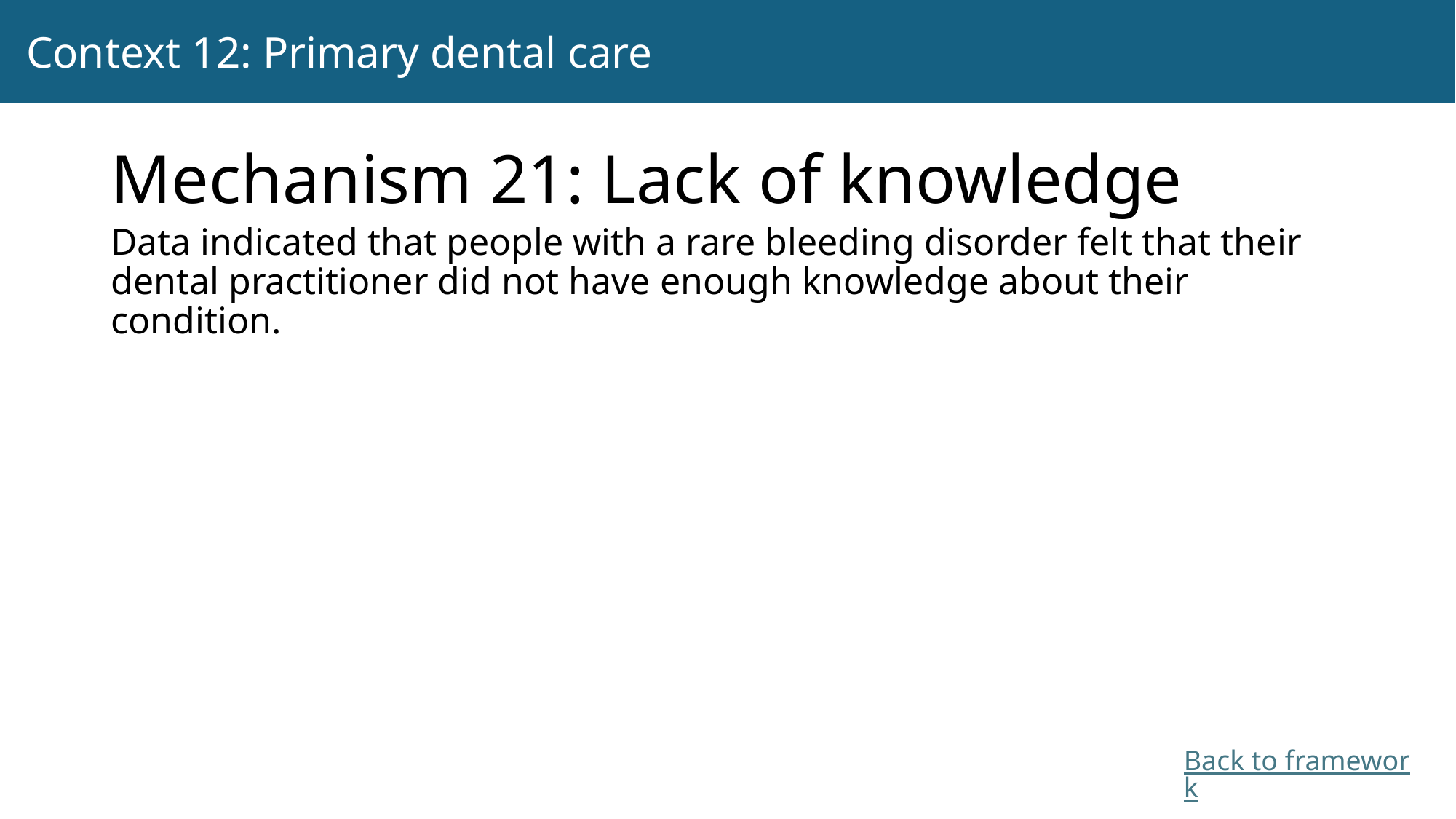

Context 12: Primary dental care
# Mechanism 21: Lack of knowledge
Data indicated that people with a rare bleeding disorder felt that their dental practitioner did not have enough knowledge about their condition.
Back to framework

## Slide 35
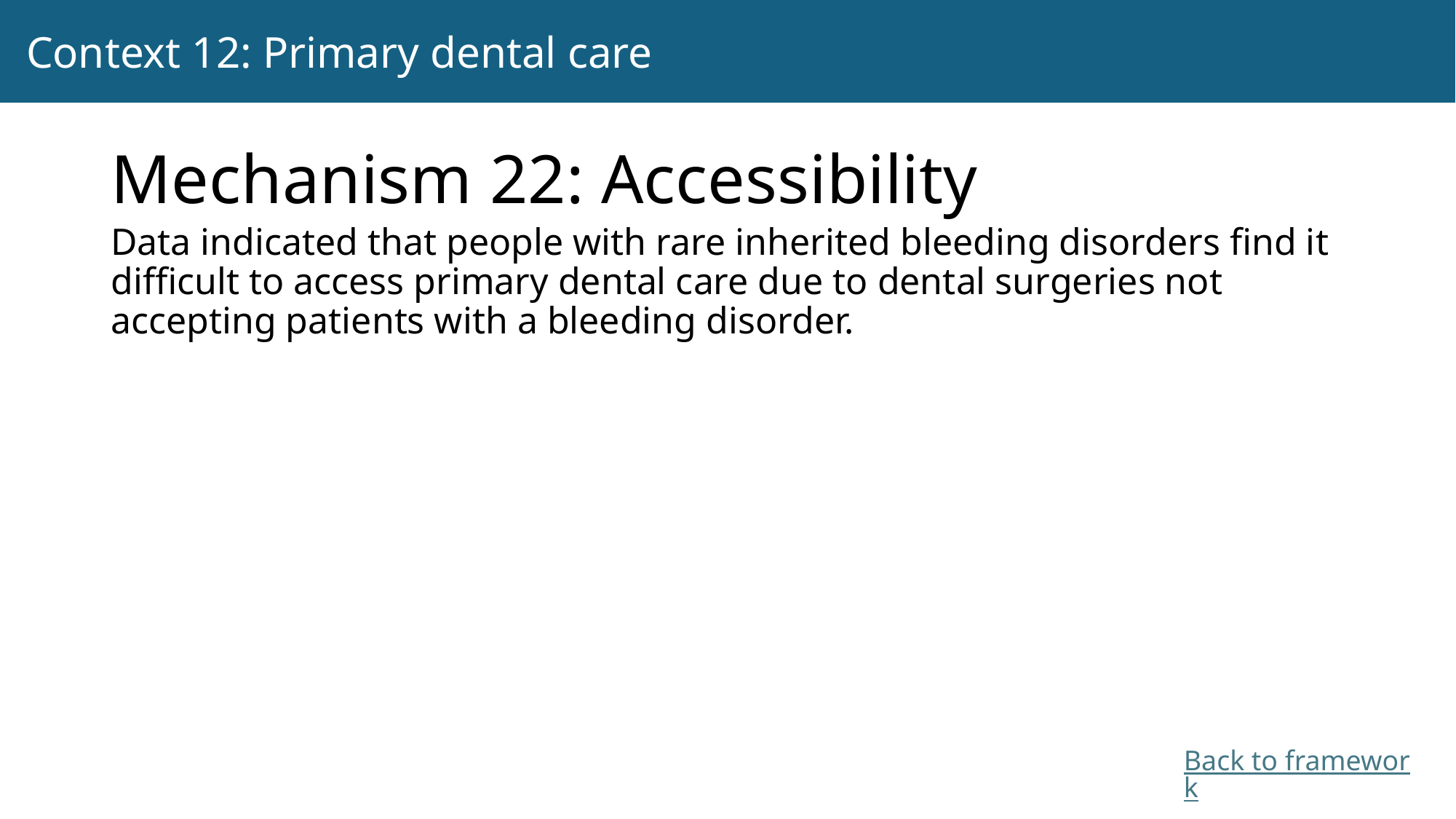

Context 12: Primary dental care
# Mechanism 22: Accessibility
Data indicated that people with rare inherited bleeding disorders find it difficult to access primary dental care due to dental surgeries not accepting patients with a bleeding disorder.
Back to framework

## Slide 36
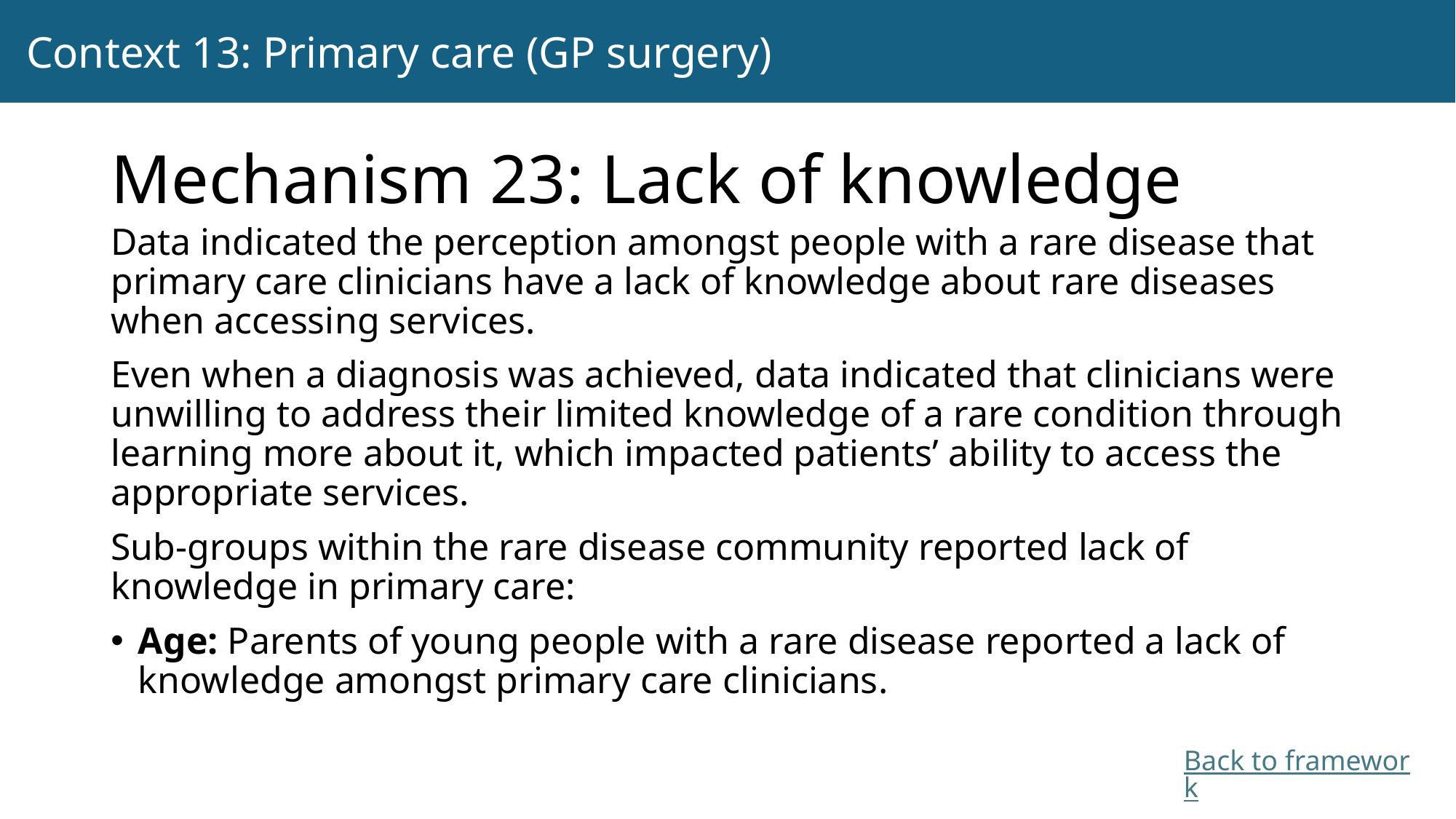

Context 13: Primary care (GP surgery)
# Mechanism 23: Lack of knowledge
Data indicated the perception amongst people with a rare disease that primary care clinicians have a lack of knowledge about rare diseases when accessing services.
Even when a diagnosis was achieved, data indicated that clinicians were unwilling to address their limited knowledge of a rare condition through learning more about it, which impacted patients’ ability to access the appropriate services.
Sub-groups within the rare disease community reported lack of knowledge in primary care:
Age: Parents of young people with a rare disease reported a lack of knowledge amongst primary care clinicians.
Back to framework

## Slide 37
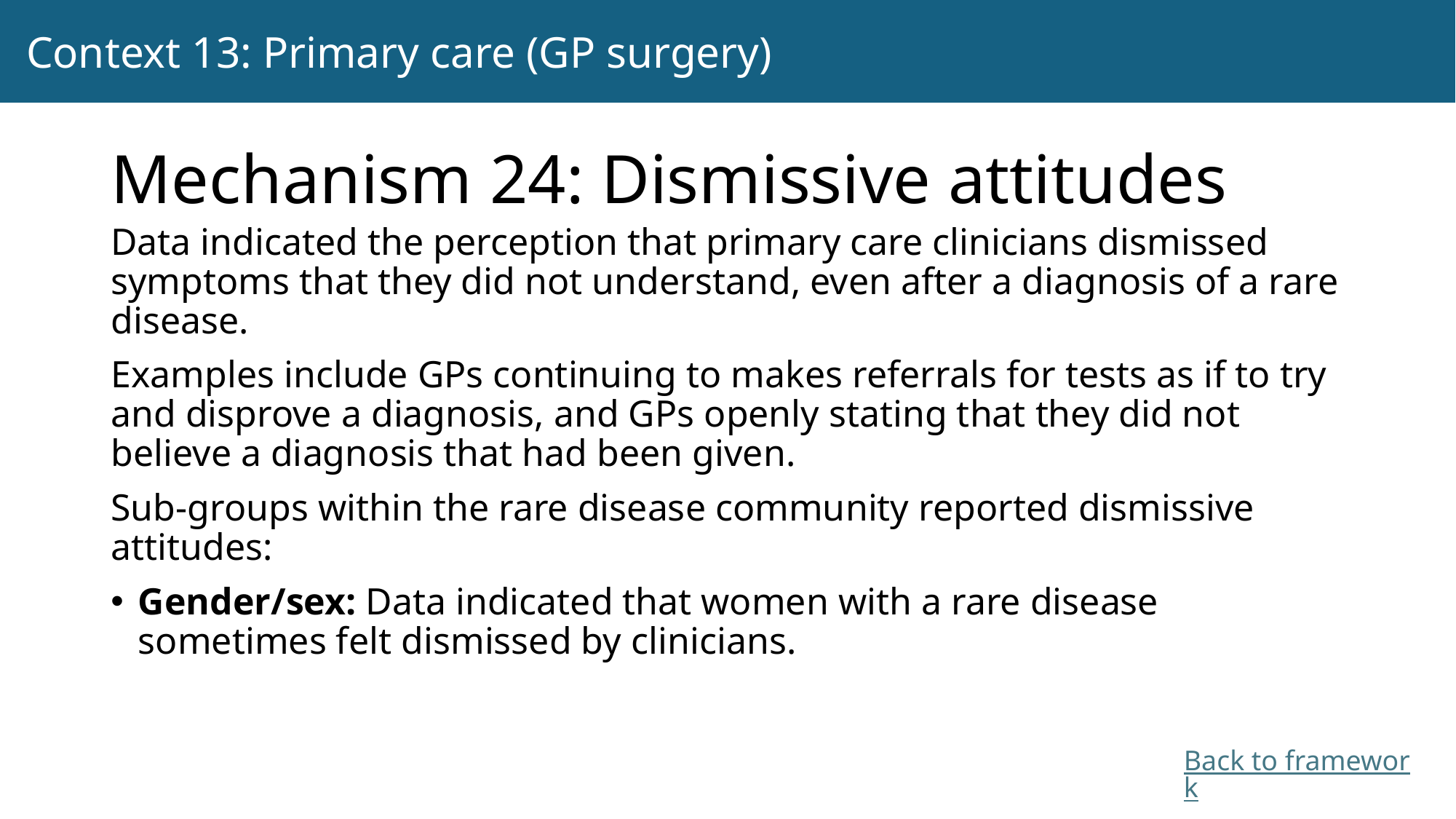

Context 13: Primary care (GP surgery)
# Mechanism 24: Dismissive attitudes
Data indicated the perception that primary care clinicians dismissed symptoms that they did not understand, even after a diagnosis of a rare disease.
Examples include GPs continuing to makes referrals for tests as if to try and disprove a diagnosis, and GPs openly stating that they did not believe a diagnosis that had been given.
Sub-groups within the rare disease community reported dismissive attitudes:
Gender/sex: Data indicated that women with a rare disease sometimes felt dismissed by clinicians.
Back to framework

## Slide 38
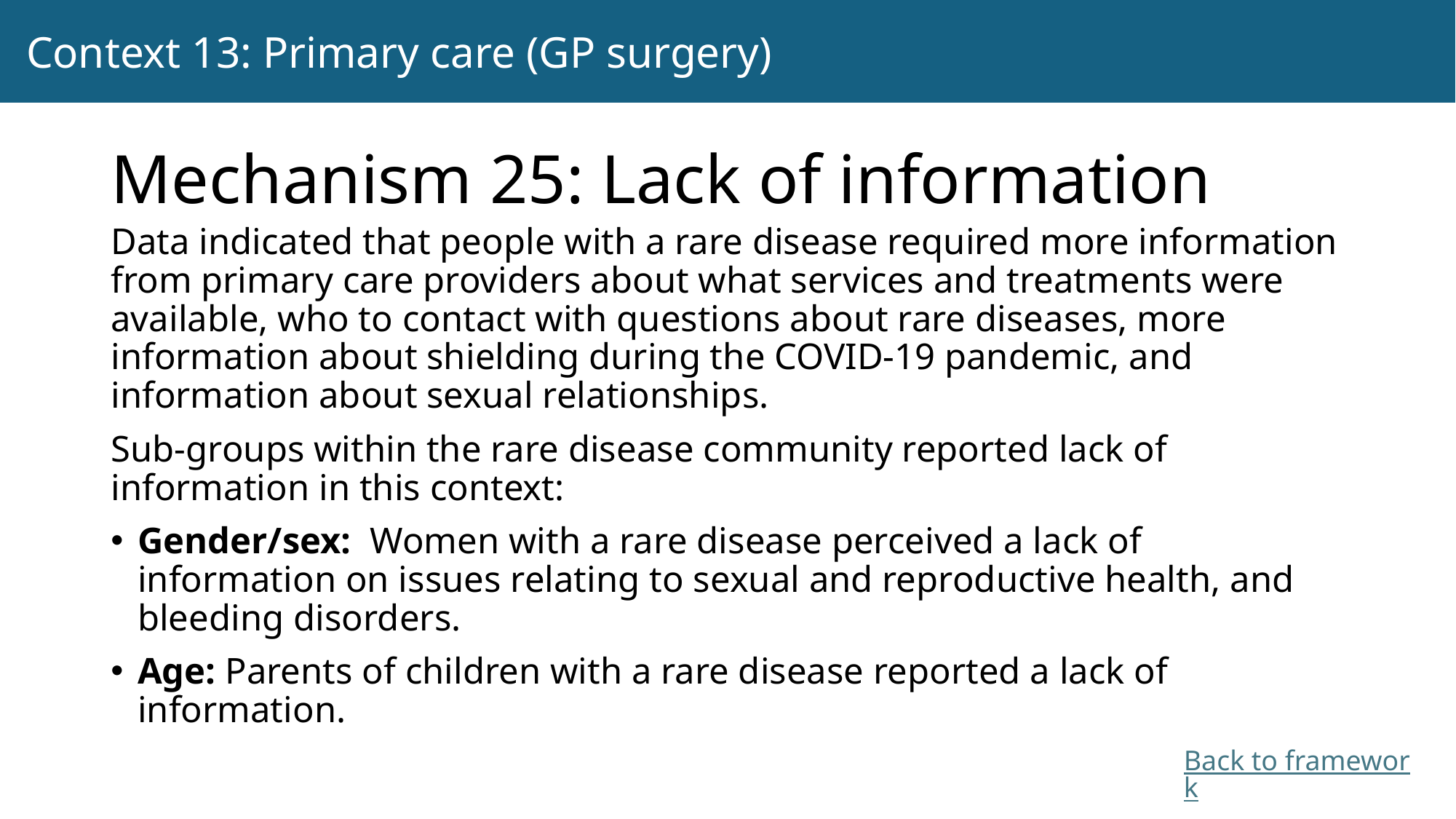

Context 13: Primary care (GP surgery)
# Mechanism 25: Lack of information
Data indicated that people with a rare disease required more information from primary care providers about what services and treatments were available, who to contact with questions about rare diseases, more information about shielding during the COVID-19 pandemic, and information about sexual relationships.
Sub-groups within the rare disease community reported lack of information in this context:
Gender/sex: Women with a rare disease perceived a lack of information on issues relating to sexual and reproductive health, and bleeding disorders.
Age: Parents of children with a rare disease reported a lack of information.
Back to framework

## Slide 39
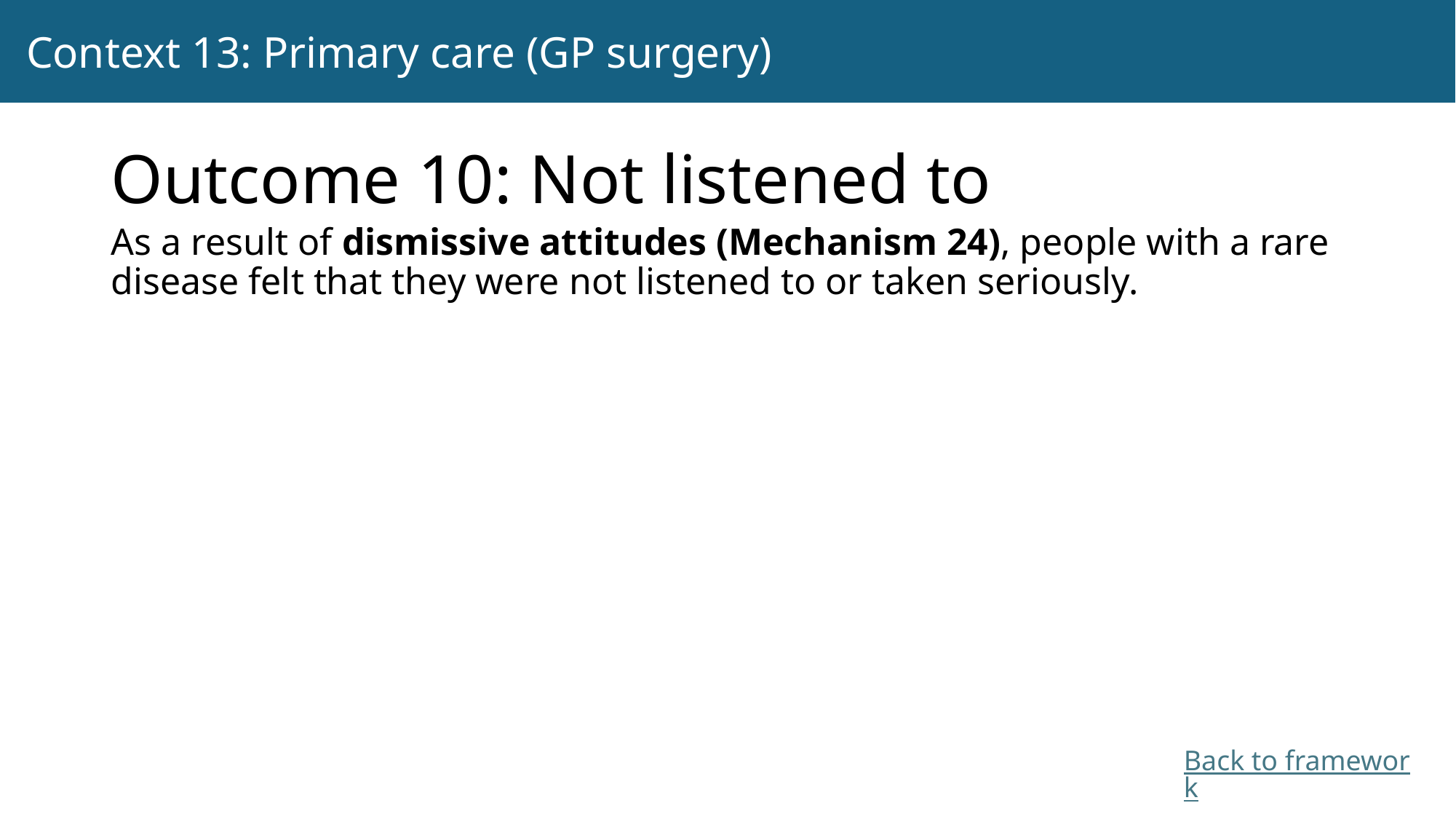

Context 13: Primary care (GP surgery)
# Outcome 10: Not listened to
As a result of dismissive attitudes (Mechanism 24), people with a rare disease felt that they were not listened to or taken seriously.
Back to framework

## Slide 40
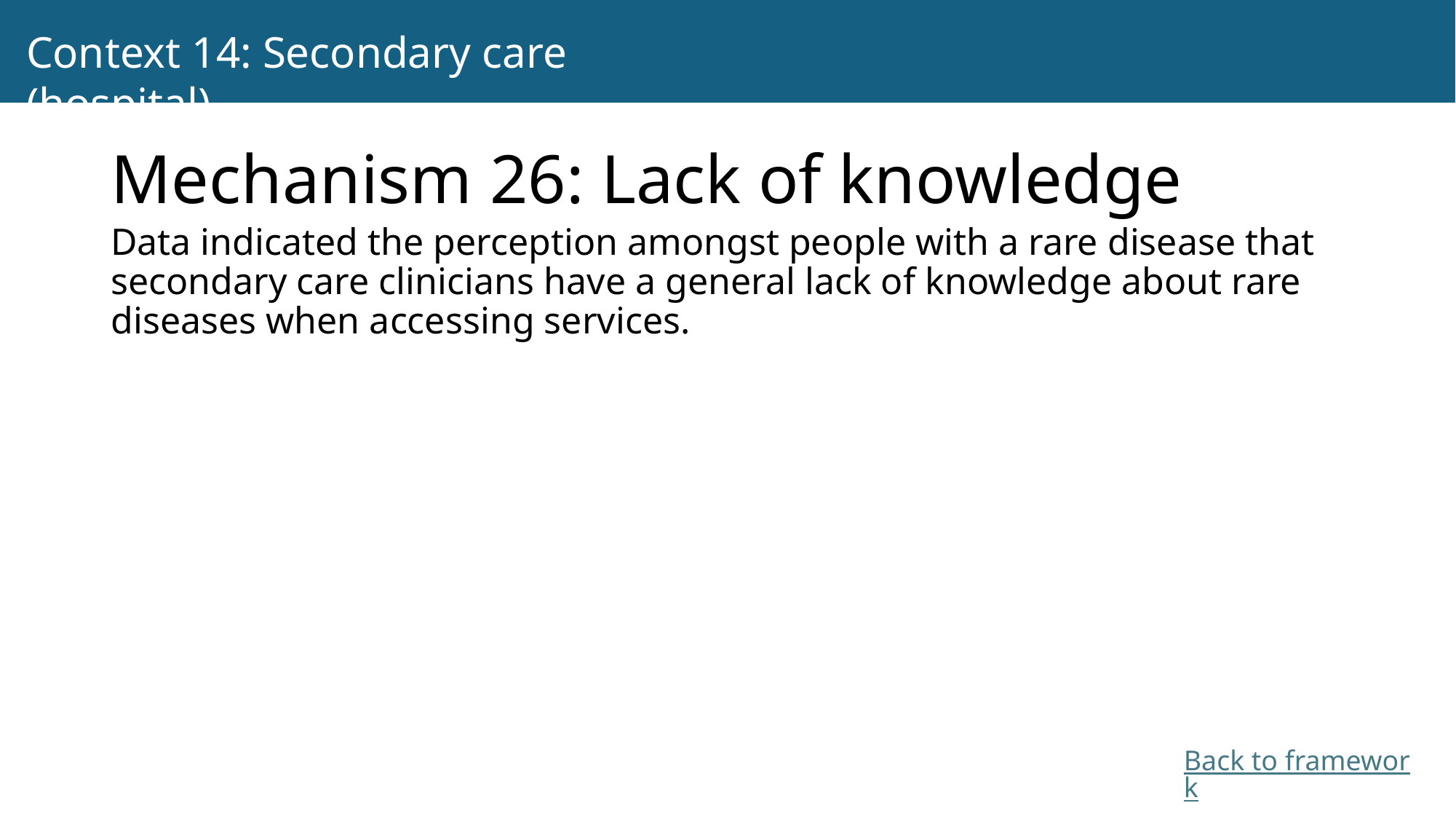

Context 14: Secondary care (hospital)
# Mechanism 26: Lack of knowledge
Data indicated the perception amongst people with a rare disease that secondary care clinicians have a general lack of knowledge about rare diseases when accessing services.
Back to framework

## Slide 41
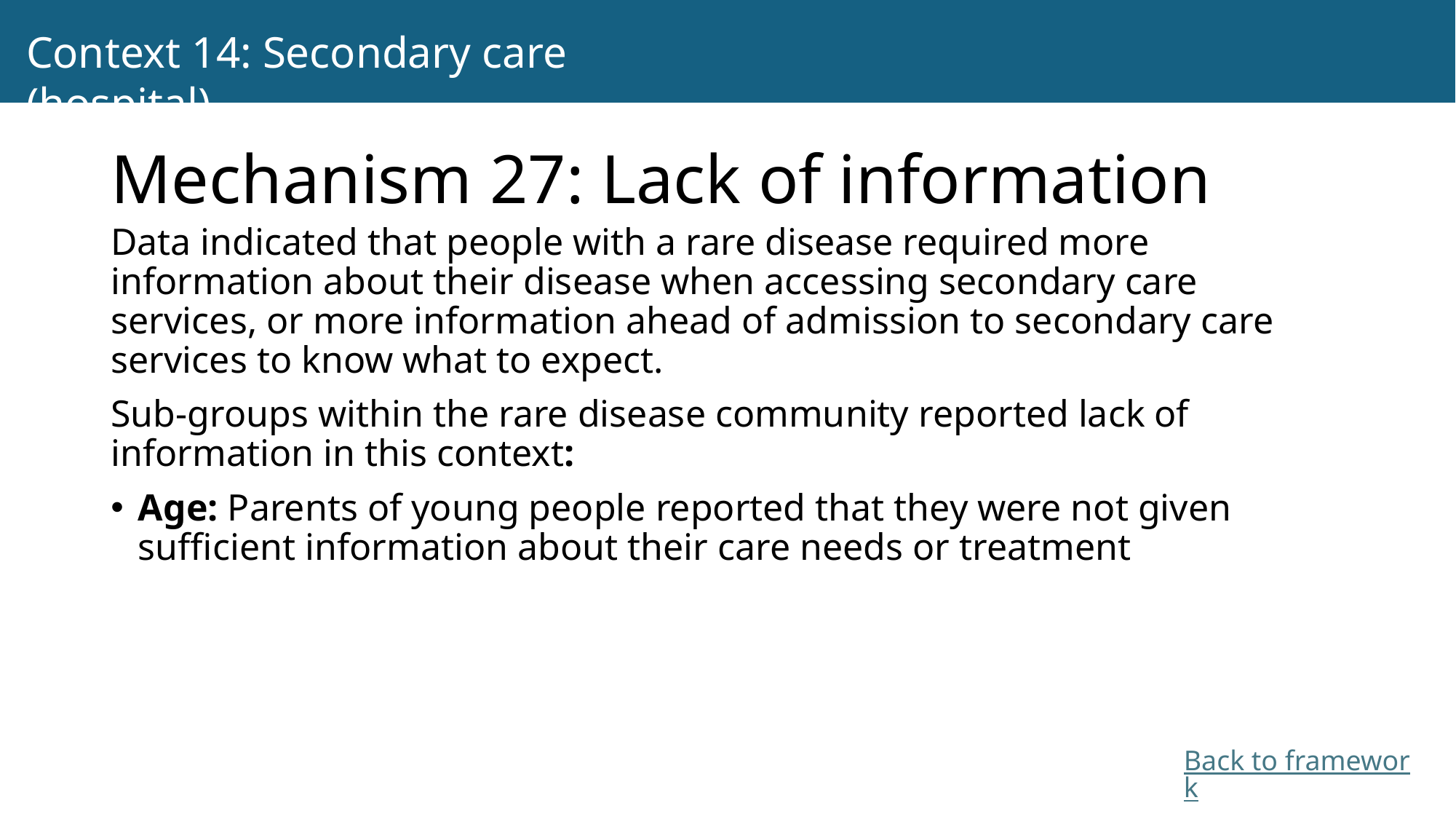

Context 14: Secondary care (hospital)
# Mechanism 27: Lack of information
Data indicated that people with a rare disease required more information about their disease when accessing secondary care services, or more information ahead of admission to secondary care services to know what to expect.
Sub-groups within the rare disease community reported lack of information in this context:
Age: Parents of young people reported that they were not given sufficient information about their care needs or treatment
Back to framework

## Slide 42
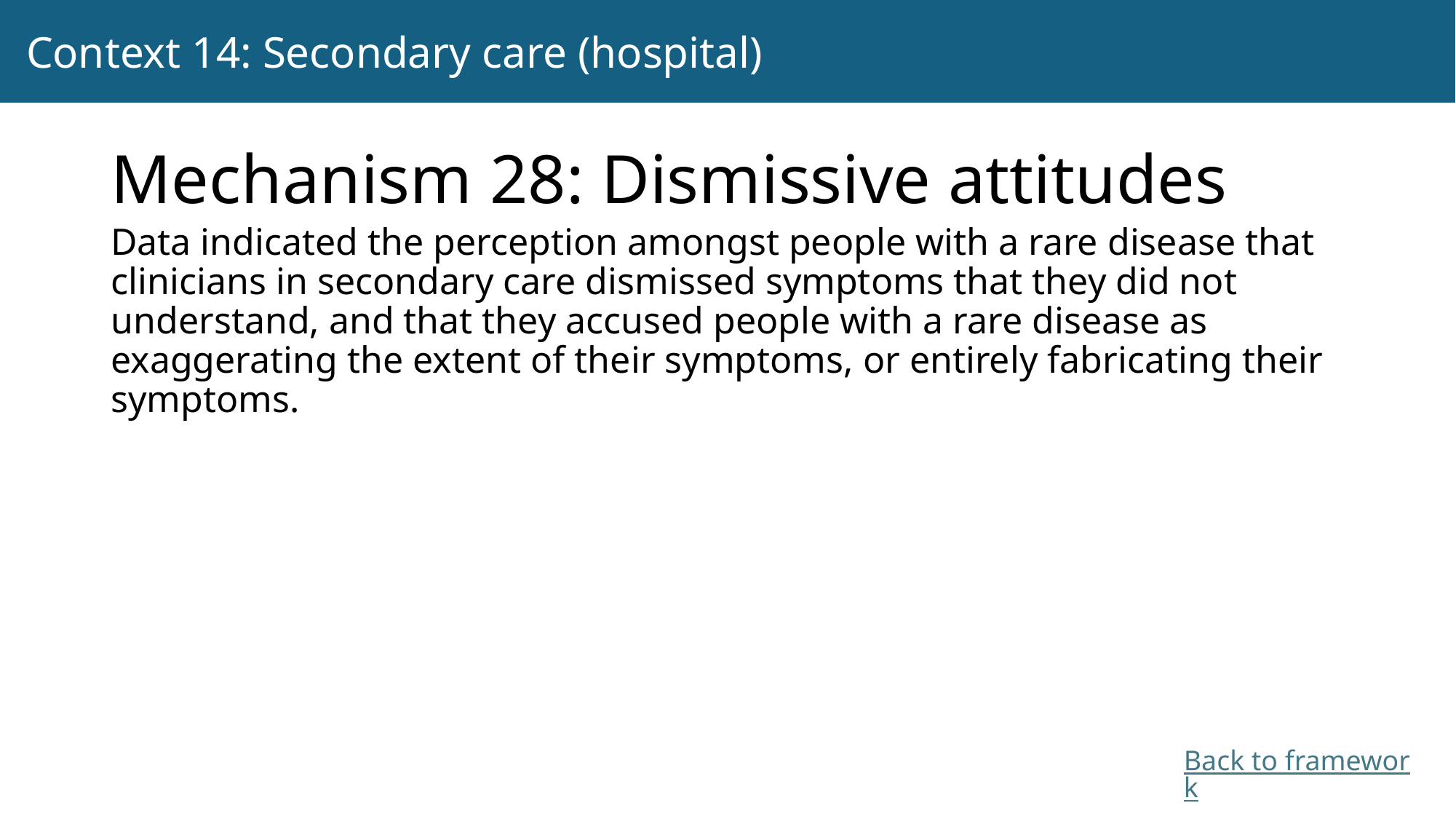

Context 14: Secondary care (hospital)
# Mechanism 28: Dismissive attitudes
Data indicated the perception amongst people with a rare disease that clinicians in secondary care dismissed symptoms that they did not understand, and that they accused people with a rare disease as exaggerating the extent of their symptoms, or entirely fabricating their symptoms.
Back to framework

## Slide 43
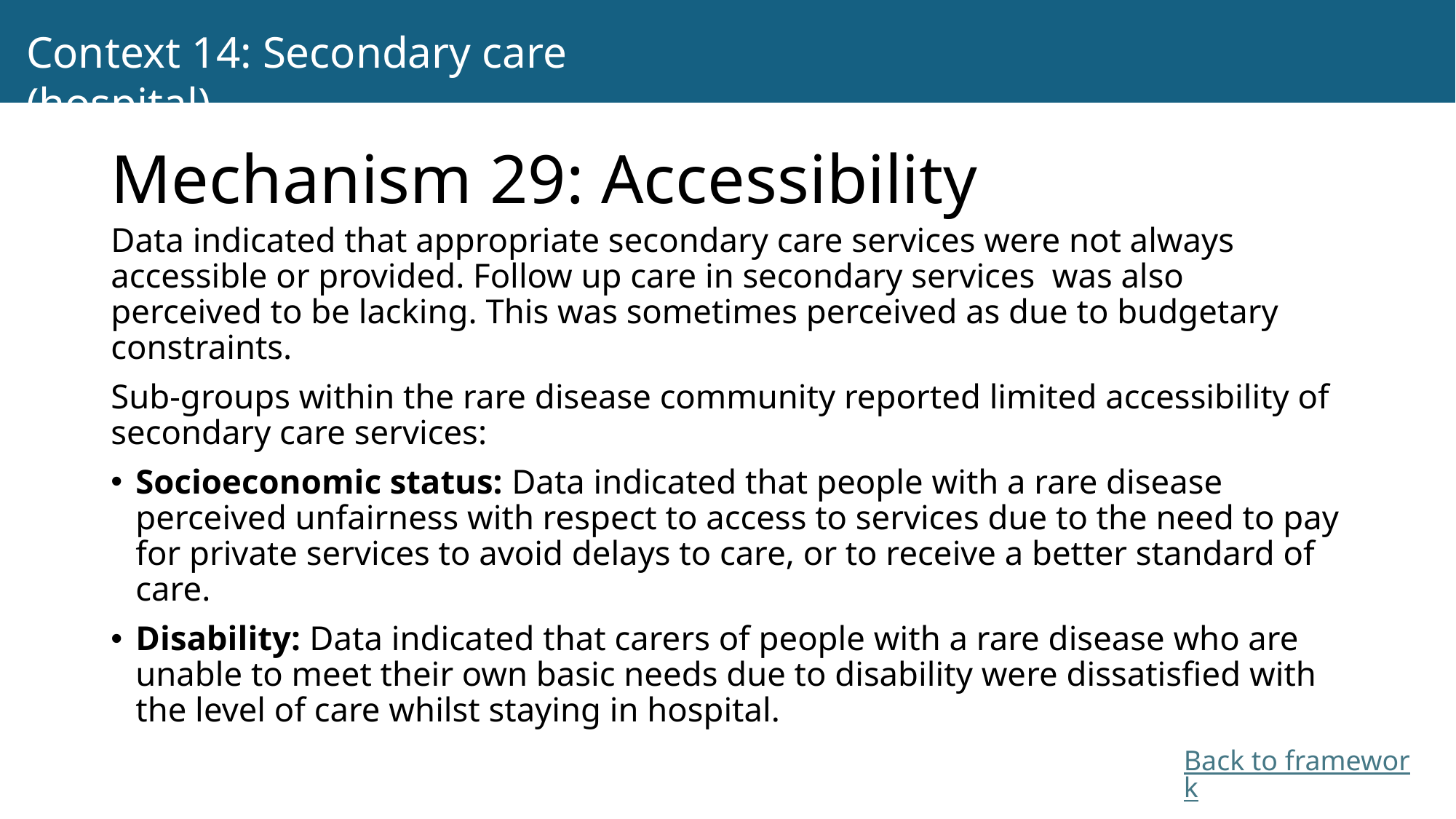

Context 14: Secondary care (hospital)
# Mechanism 29: Accessibility
Data indicated that appropriate secondary care services were not always accessible or provided. Follow up care in secondary services was also perceived to be lacking. This was sometimes perceived as due to budgetary constraints.
Sub-groups within the rare disease community reported limited accessibility of secondary care services:
Socioeconomic status: Data indicated that people with a rare disease perceived unfairness with respect to access to services due to the need to pay for private services to avoid delays to care, or to receive a better standard of care.
Disability: Data indicated that carers of people with a rare disease who are unable to meet their own basic needs due to disability were dissatisfied with the level of care whilst staying in hospital.
Back to framework

## Slide 44
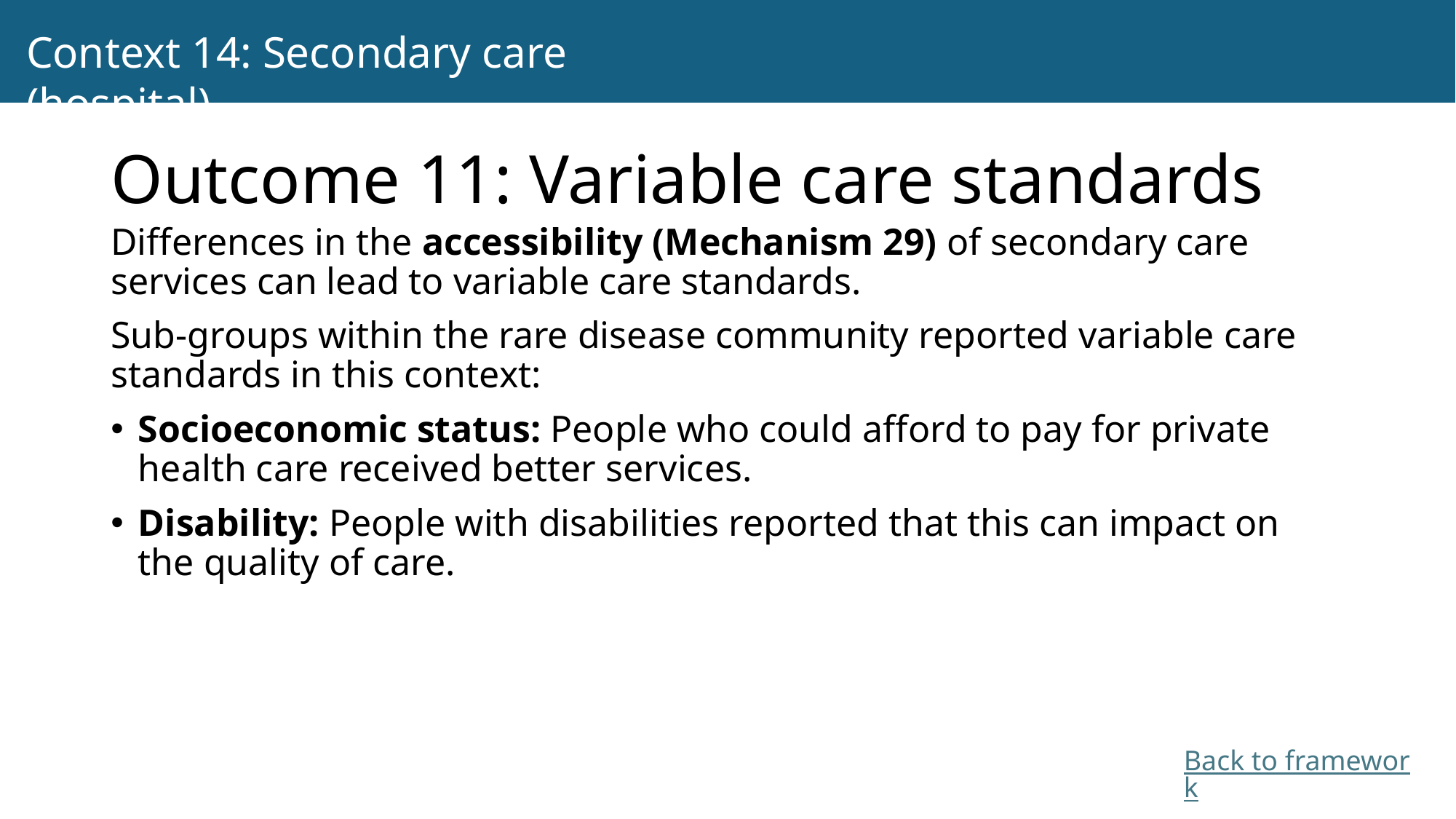

Context 14: Secondary care (hospital)
# Outcome 11: Variable care standards
Differences in the accessibility (Mechanism 29) of secondary care services can lead to variable care standards.
Sub-groups within the rare disease community reported variable care standards in this context:
Socioeconomic status: People who could afford to pay for private health care received better services.
Disability: People with disabilities reported that this can impact on the quality of care.
Back to framework

## Slide 45
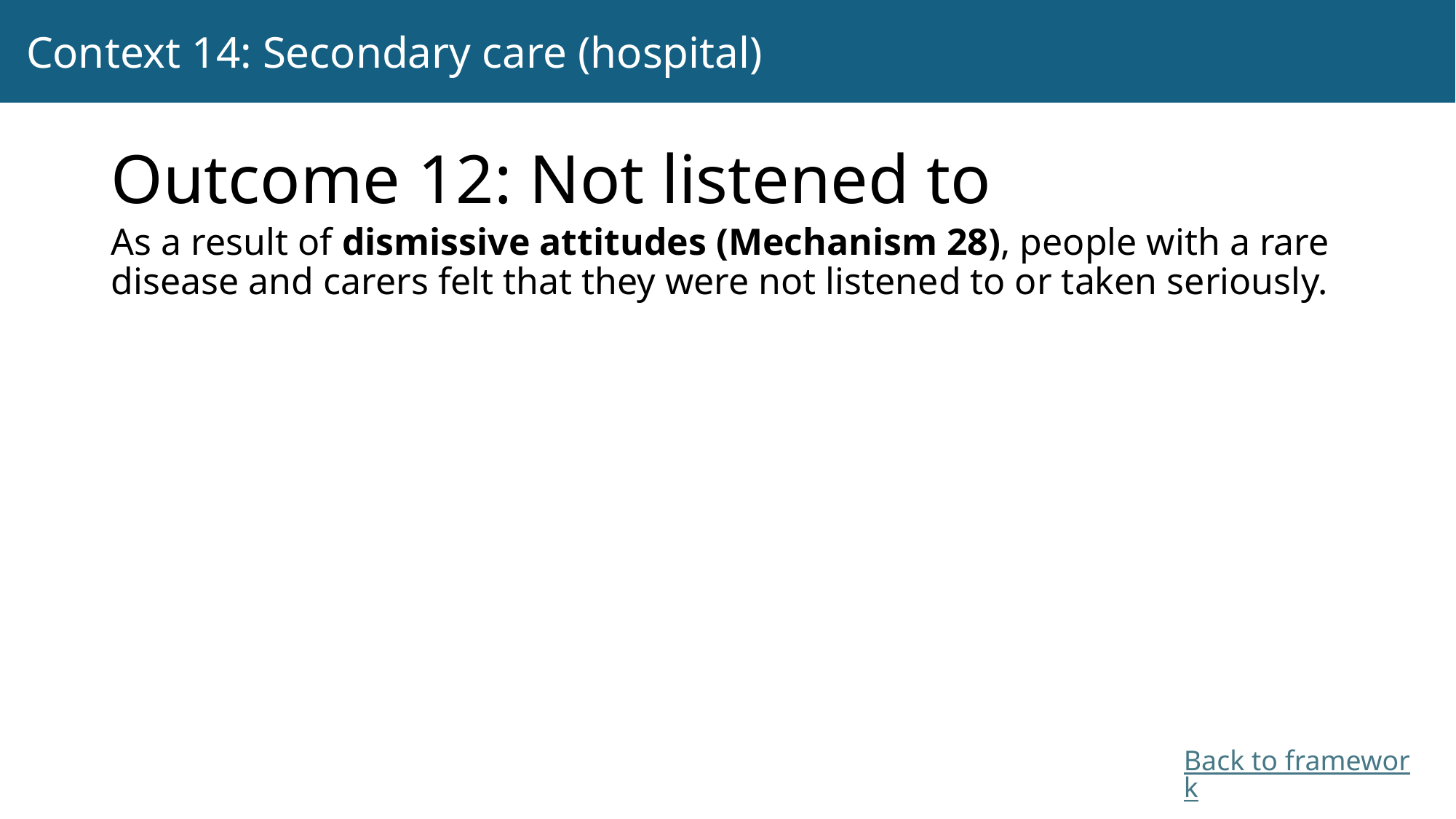

Context 14: Secondary care (hospital)
# Outcome 12: Not listened to
As a result of dismissive attitudes (Mechanism 28), people with a rare disease and carers felt that they were not listened to or taken seriously.
Back to framework

## Slide 46
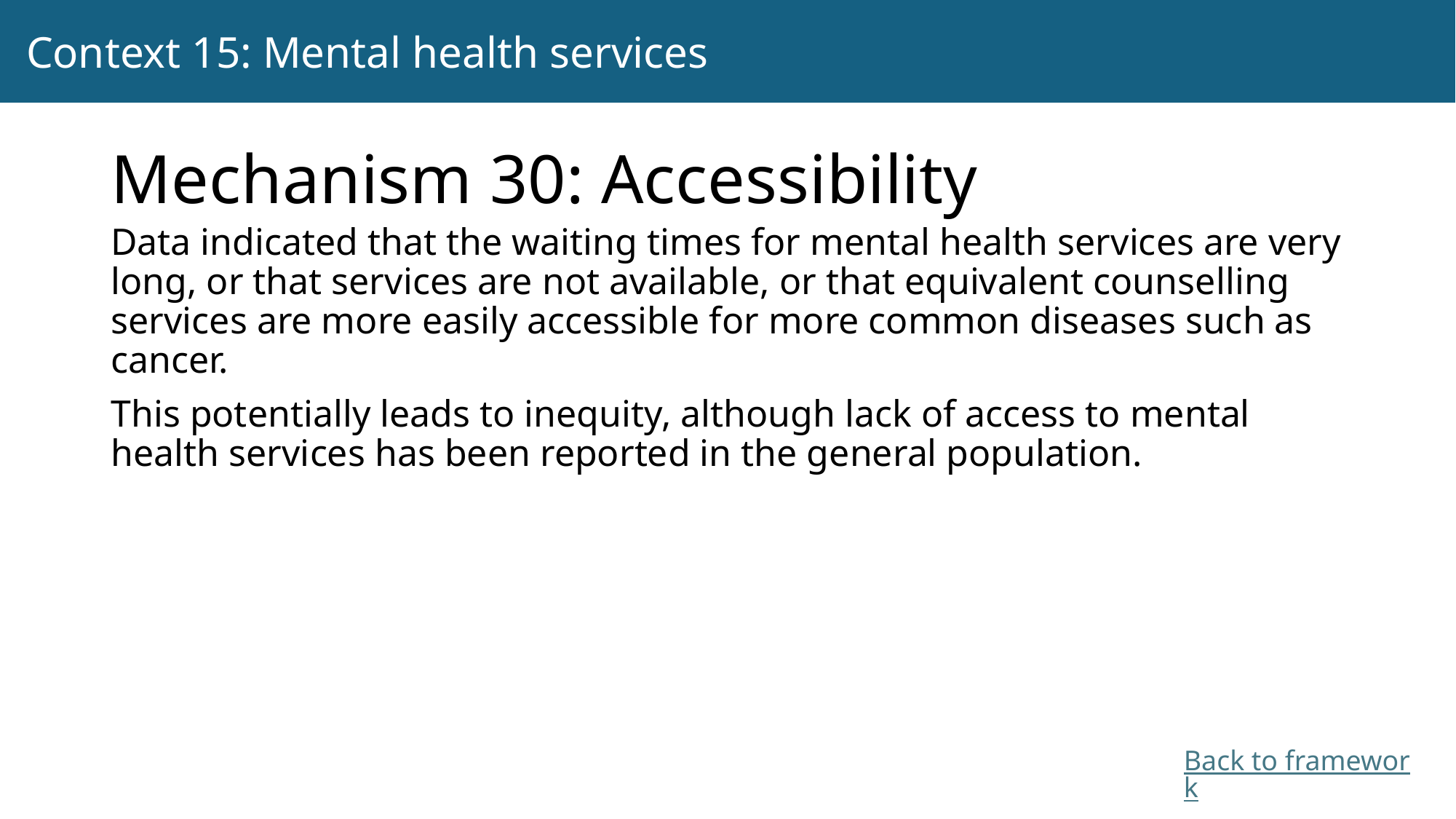

Context 15: Mental health services
# Mechanism 30: Accessibility
Data indicated that the waiting times for mental health services are very long, or that services are not available, or that equivalent counselling services are more easily accessible for more common diseases such as cancer.
This potentially leads to inequity, although lack of access to mental health services has been reported in the general population.
Back to framework

## Slide 47
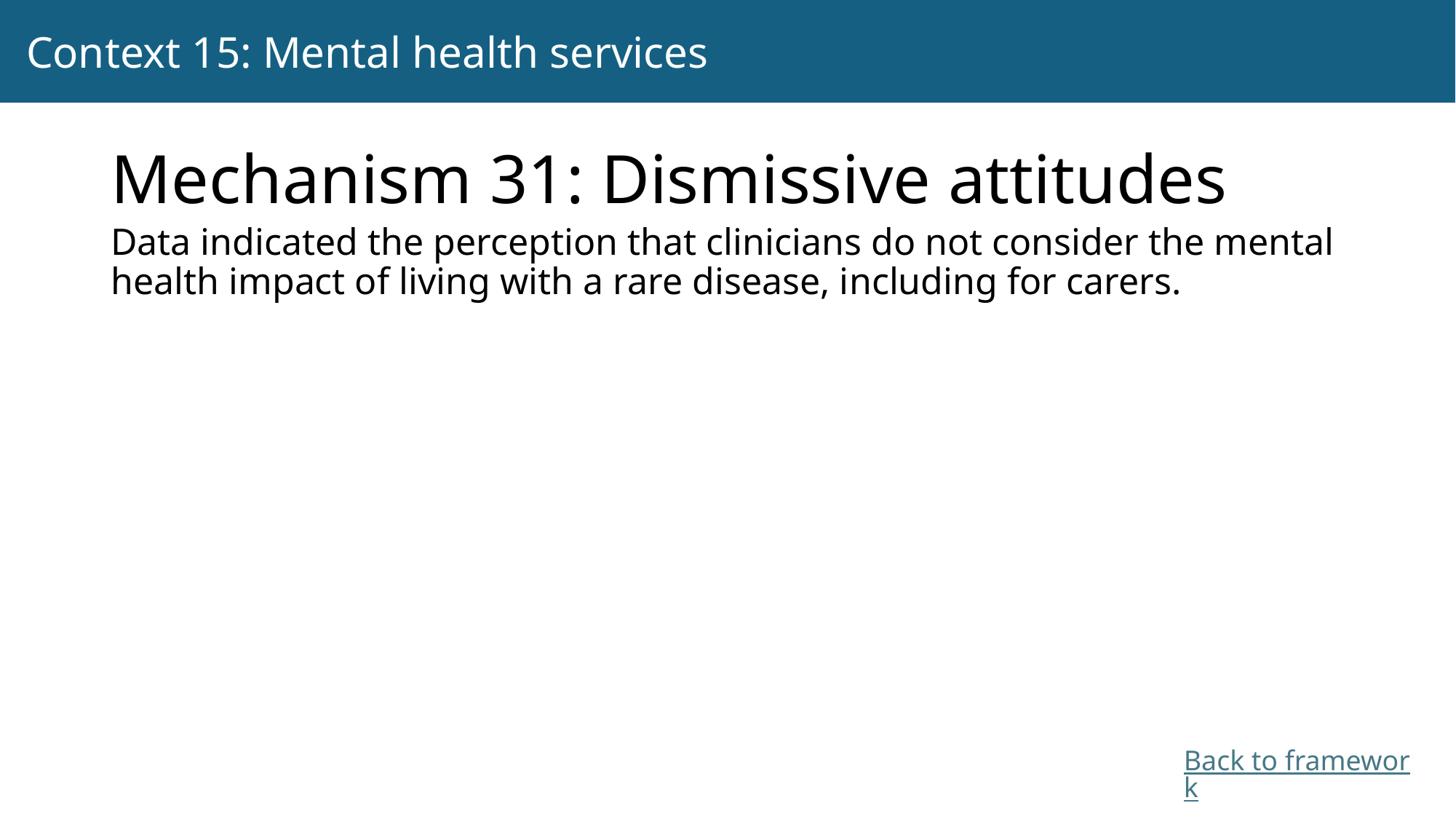

Context 15: Mental health services
# Mechanism 31: Dismissive attitudes
Data indicated the perception that clinicians do not consider the mental health impact of living with a rare disease, including for carers.
Back to framework

## Slide 48
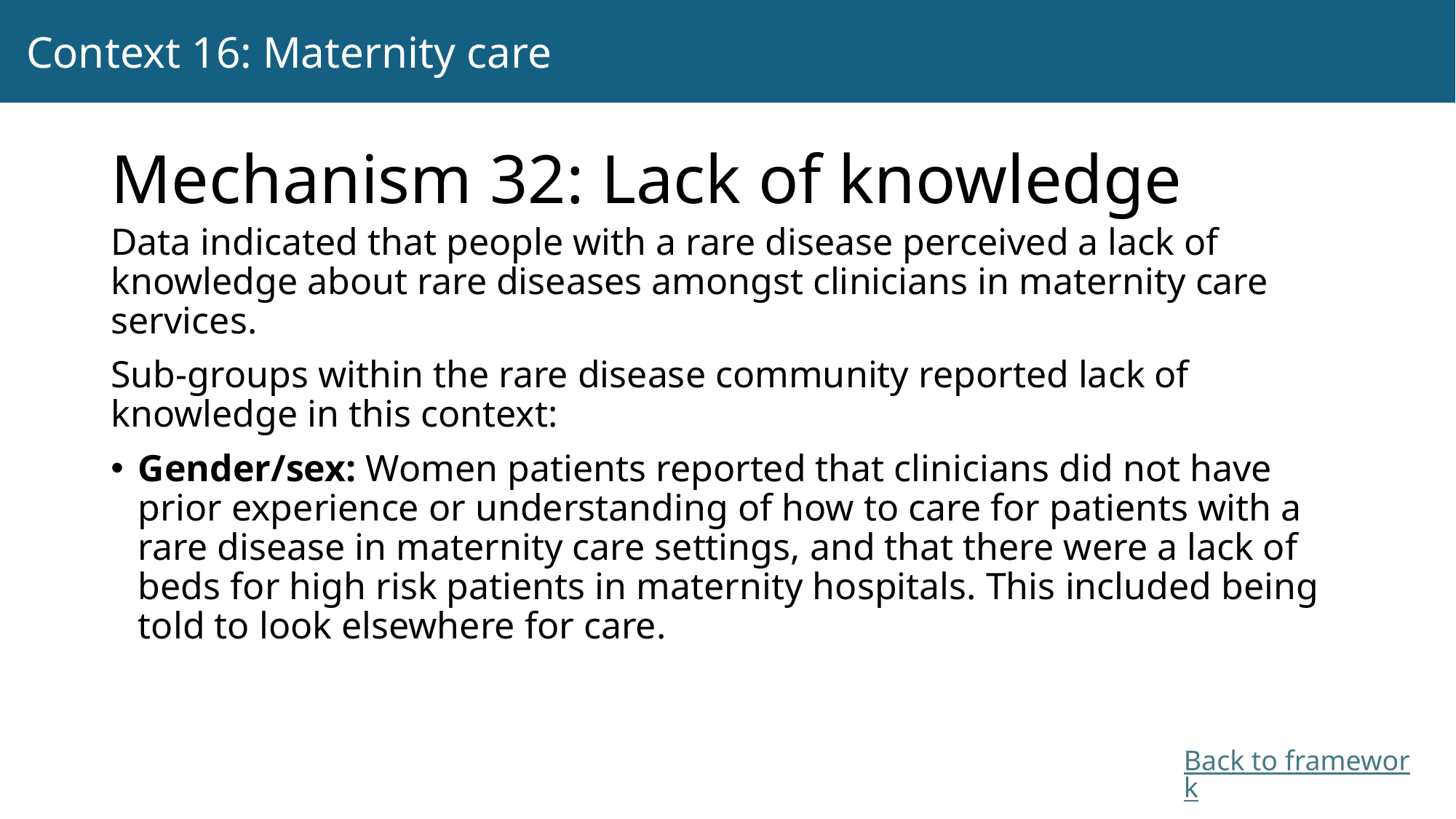

Context 16: Maternity care
# Mechanism 32: Lack of knowledge
Data indicated that people with a rare disease perceived a lack of knowledge about rare diseases amongst clinicians in maternity care services.
Sub-groups within the rare disease community reported lack of knowledge in this context:
Gender/sex: Women patients reported that clinicians did not have prior experience or understanding of how to care for patients with a rare disease in maternity care settings, and that there were a lack of beds for high risk patients in maternity hospitals. This included being told to look elsewhere for care.
Back to framework

## Slide 49
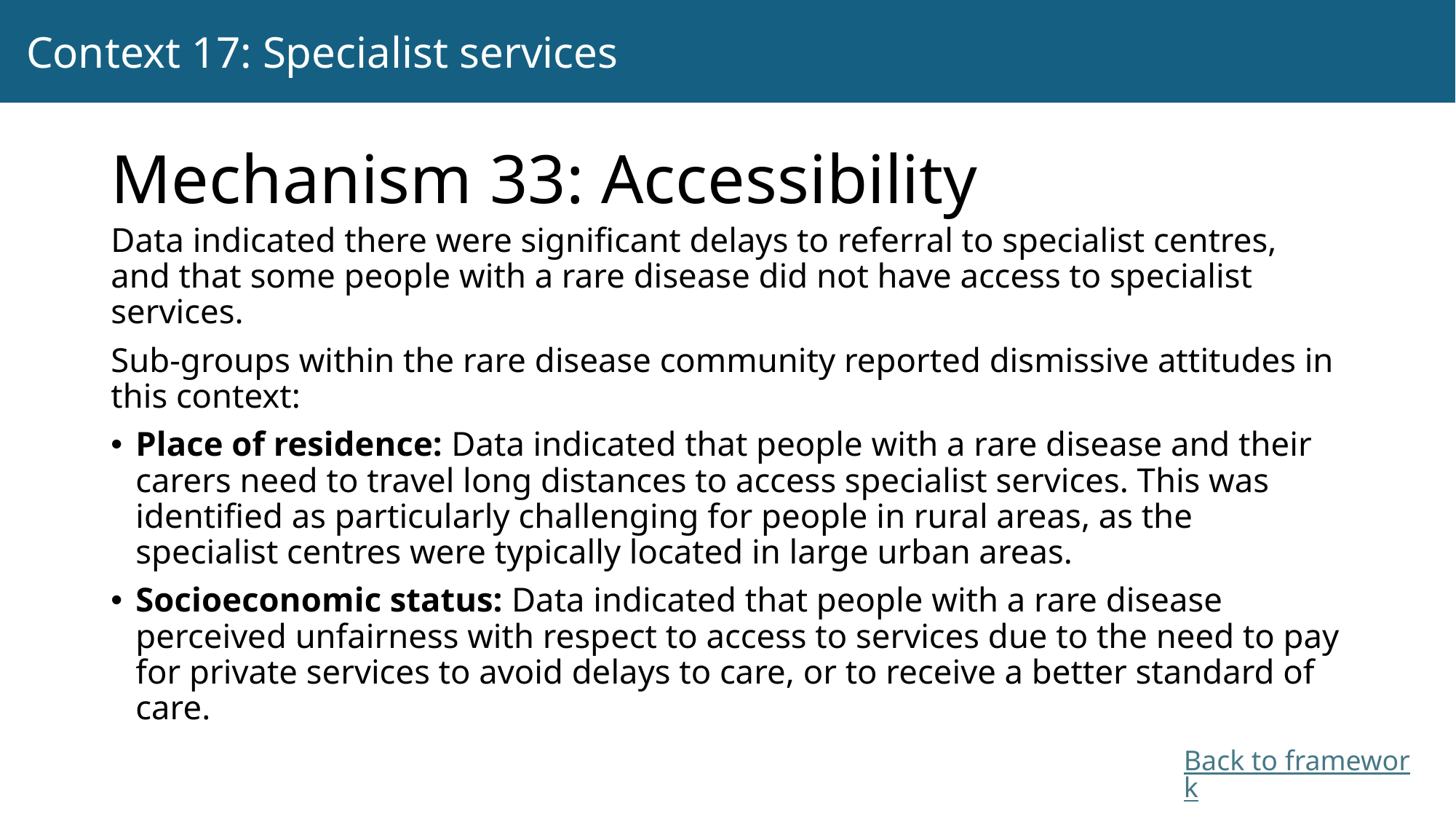

Context 17: Specialist services
# Mechanism 33: Accessibility
Data indicated there were significant delays to referral to specialist centres, and that some people with a rare disease did not have access to specialist services.
Sub-groups within the rare disease community reported dismissive attitudes in this context:
Place of residence: Data indicated that people with a rare disease and their carers need to travel long distances to access specialist services. This was identified as particularly challenging for people in rural areas, as the specialist centres were typically located in large urban areas.
Socioeconomic status: Data indicated that people with a rare disease perceived unfairness with respect to access to services due to the need to pay for private services to avoid delays to care, or to receive a better standard of care.
Back to framework

## Slide 50
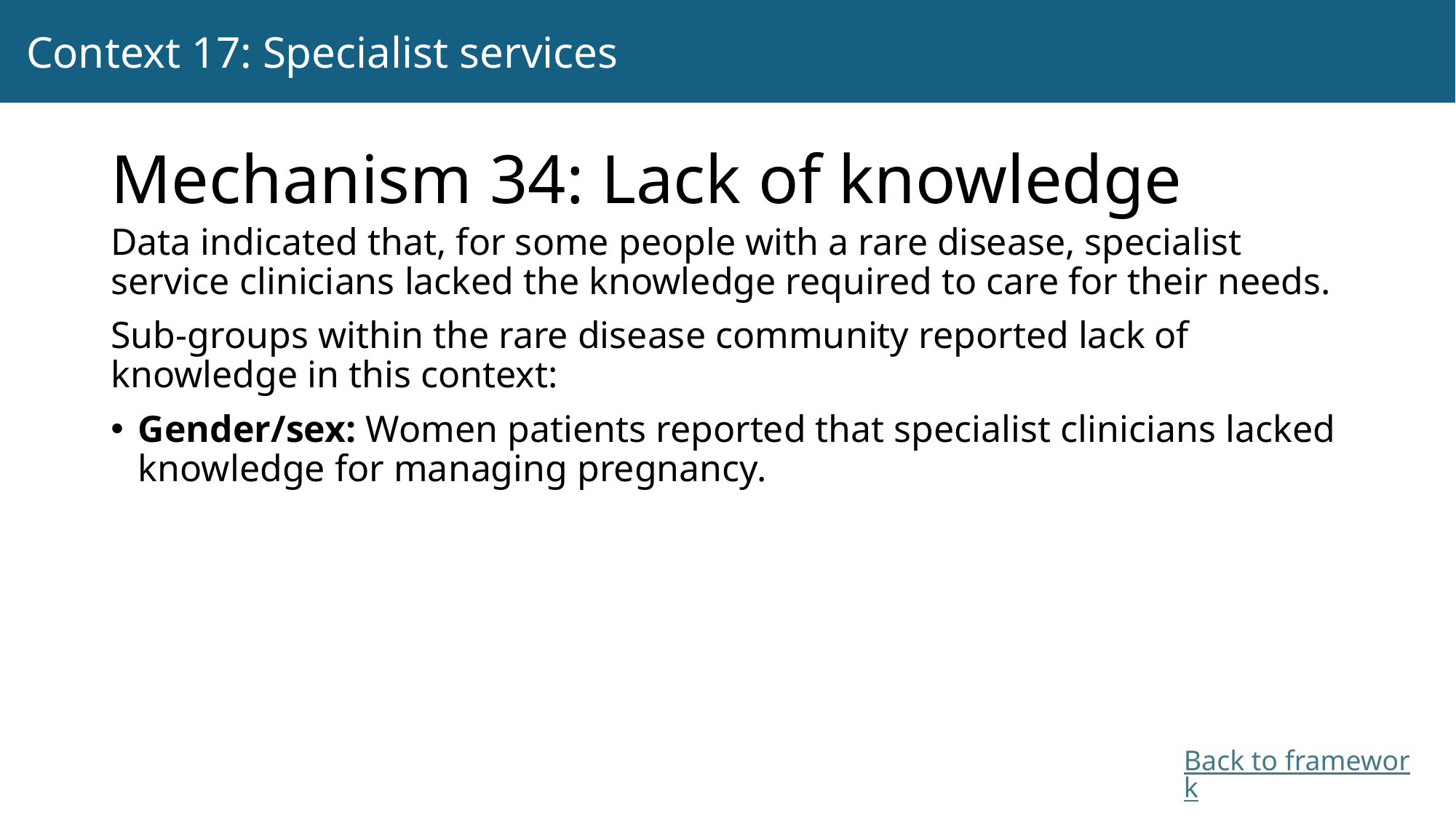

Context 17: Specialist services
# Mechanism 34: Lack of knowledge
Data indicated that, for some people with a rare disease, specialist service clinicians lacked the knowledge required to care for their needs.
Sub-groups within the rare disease community reported lack of knowledge in this context:
Gender/sex: Women patients reported that specialist clinicians lacked knowledge for managing pregnancy.
Back to framework

## Slide 51
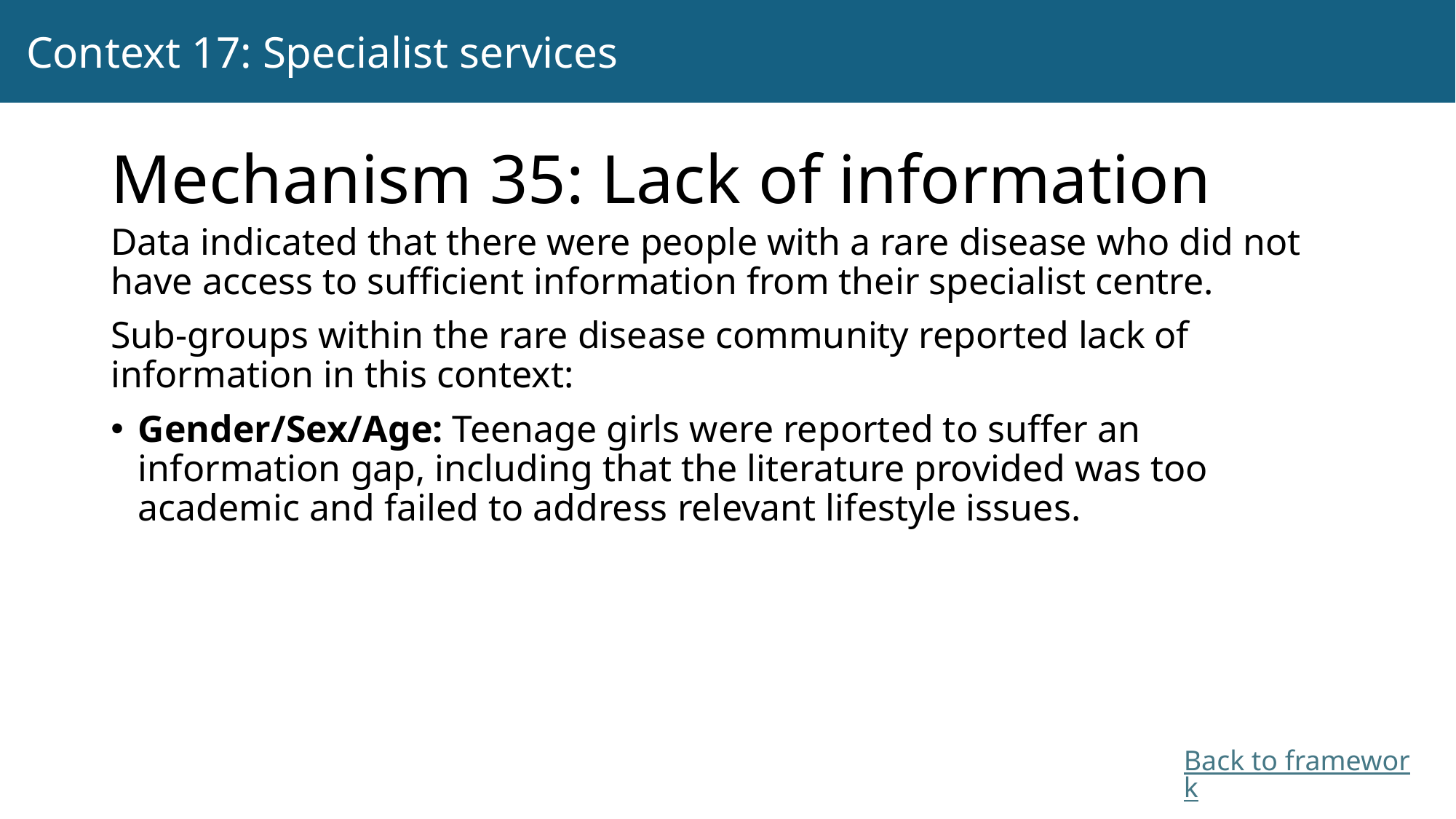

Context 17: Specialist services
# Mechanism 35: Lack of information
Data indicated that there were people with a rare disease who did not have access to sufficient information from their specialist centre.
Sub-groups within the rare disease community reported lack of information in this context:
Gender/Sex/Age: Teenage girls were reported to suffer an information gap, including that the literature provided was too academic and failed to address relevant lifestyle issues.
Back to framework

## Slide 52
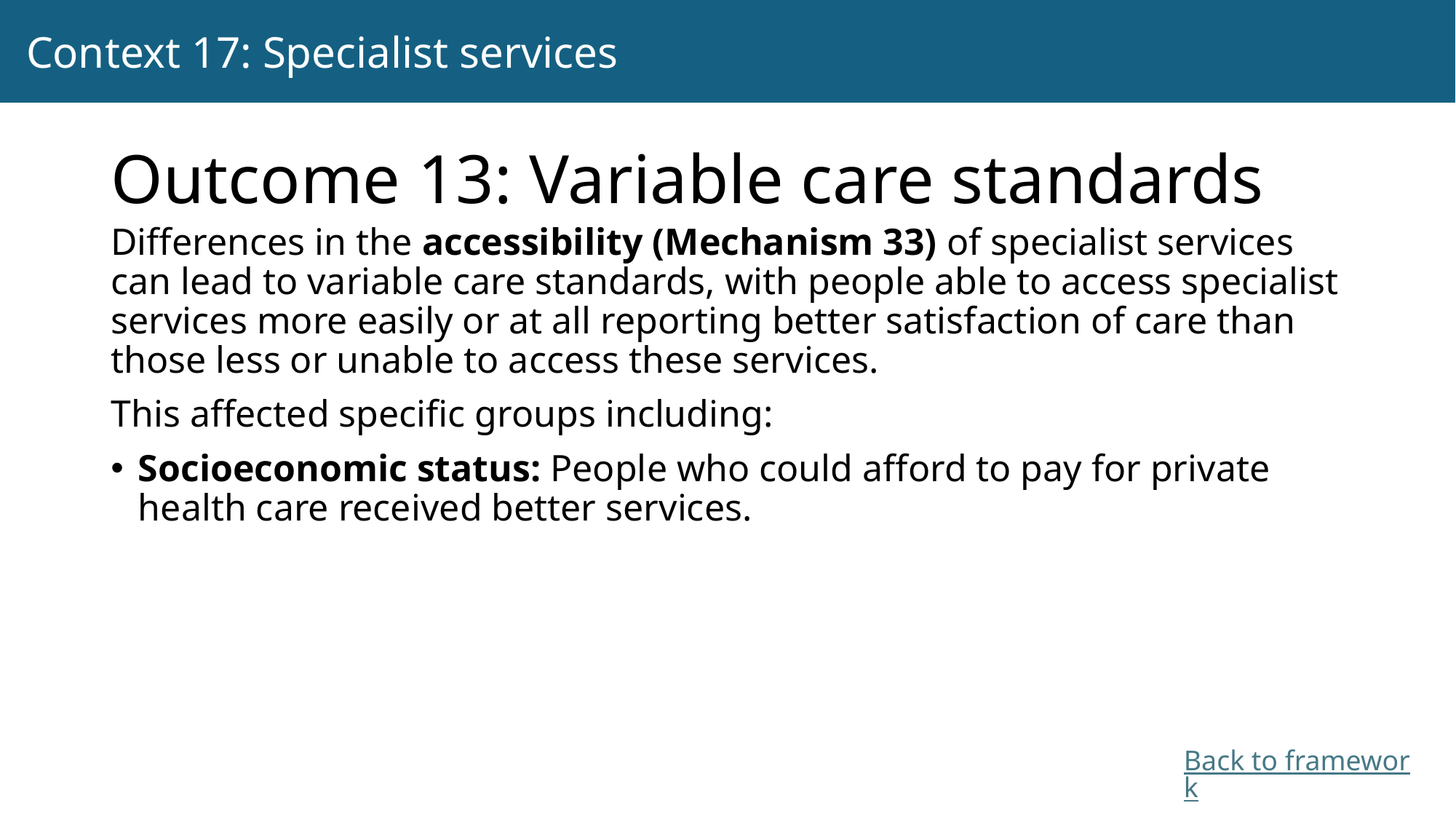

Context 17: Specialist services
# Outcome 13: Variable care standards
Differences in the accessibility (Mechanism 33) of specialist services can lead to variable care standards, with people able to access specialist services more easily or at all reporting better satisfaction of care than those less or unable to access these services.
This affected specific groups including:
Socioeconomic status: People who could afford to pay for private health care received better services.
Back to framework

## Slide 53
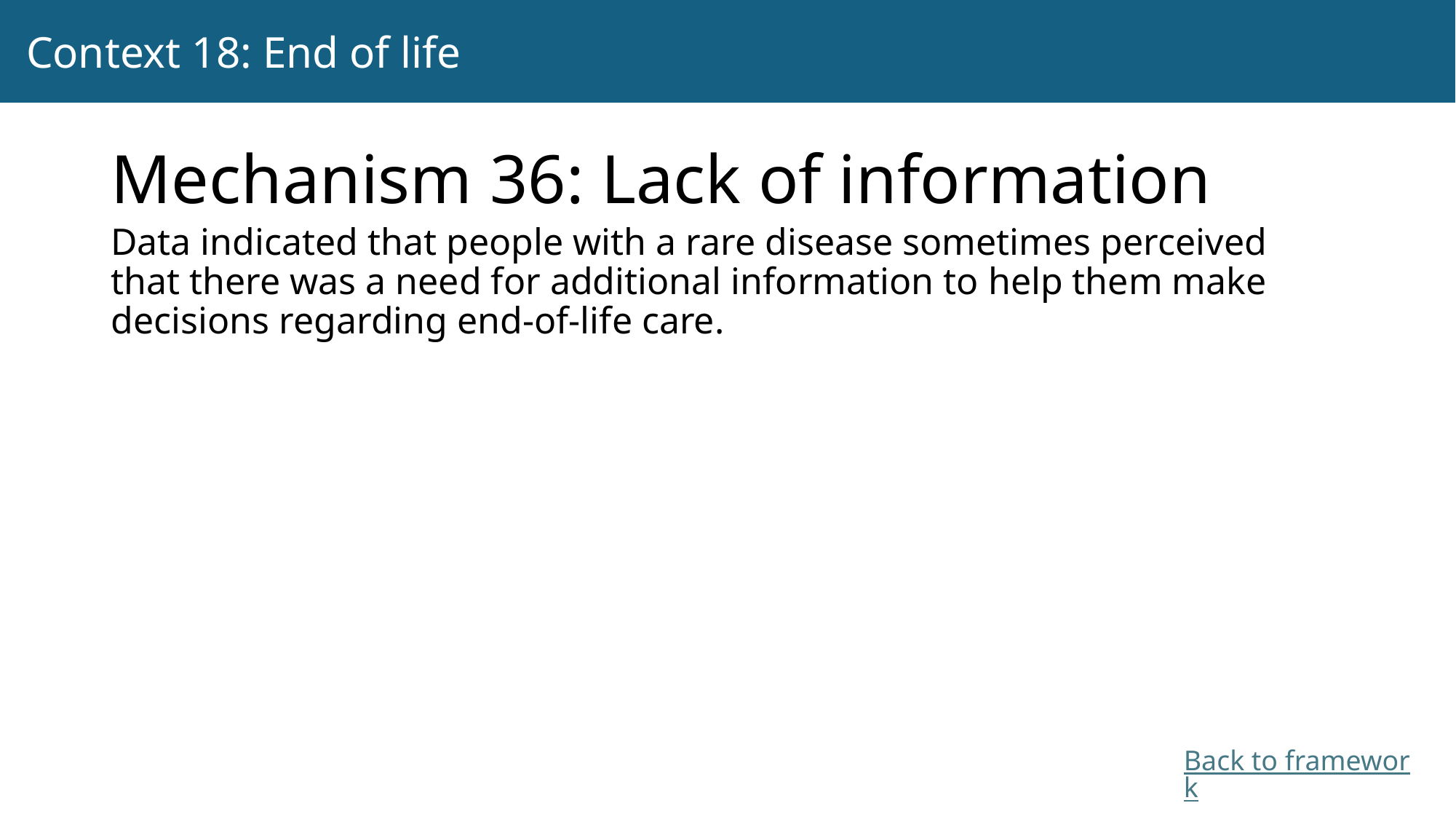

Context 18: End of life
# Mechanism 36: Lack of information
Data indicated that people with a rare disease sometimes perceived that there was a need for additional information to help them make decisions regarding end-of-life care.
Back to framework
